# Microbial and human transcriptional profiling of coronavirus disease 2019 patients: Potential predictors of disease severity

**DOI:** 10.3389/fmicb.2022.959433

**Published:** 2022-09-02

**Authors:** Hairun Gan, Jiumeng Min, Haoyu Long, Bing Li, Xinyan Hu, Zhongyi Zhu, Luting Li, Tiancheng Wang, Xiangyan He, Jianxun Cai, Yongyu Zhang, Jianan He, Luan Chen, Dashuai Wang, Jintao Su, Ni Zhao, Weile Huang, Jingjing Zhang, Ziqi Su, Hui Guo, Xiaojun Hu, Junjie Mao, Jinmin Ma, Pengfei Pang

**Affiliations:** ^1^Center for Interventional Medicine, The Fifth Affiliated Hospital, Sun Yat-sen University, Zhuhai, China; ^2^Guangdong Provincial Key Laboratory of Biomedical Imaging, The Fifth Affiliated Hospital, Sun Yat-sen University, Zhuhai, China; ^3^Guangdong Provincial Engineering Research Center of Molecular Imaging, The Fifth Affiliated Hospital, Sun Yat-sen University, Zhuhai, China; ^4^BGI PathoGenesis Pharmaceutical Technology, BGI-Shenzhen, Shenzhen, China; ^5^Clinical Laboratory of BGI Health, BGI-Shenzhen, Shenzhen, China; ^6^Department of Ophthalmology, The Fifth Affiliated Hospital, Sun Yat-sen University, Zhuhai, China

**Keywords:** SARS-CoV-2, COVID-19, microbiome, host response, diagnostic biomarkers, transcriptomic sequencing

## Abstract

The high morbidity of patients with coronavirus disease 2019 (COVID-19) brings on a panic around the world. COVID-19 is associated with sex bias, immune system, and preexisting chronic diseases. We analyzed the gene expression in patients with COVID-19 and in their microbiota in order to identify potential biomarkers to aid in disease management. A total of 129 RNA samples from nasopharyngeal, oropharyngeal, and anal swabs were collected and sequenced in a high-throughput manner. Several microbial strains differed in abundance between patients with mild or severe COVID-19. Microbial genera were more abundant in oropharyngeal swabs than in nasopharyngeal or anal swabs. Oropharyngeal swabs allowed more sensitive detection of the causative SARS-CoV-2. Microbial and human transcriptomes in swabs from patients with mild disease showed enrichment of genes involved in amino acid metabolism, or protein modification *via* small protein removal, and antibacterial defense responses, respectively, whereas swabs from patients with severe disease showed enrichment of genes involved in drug metabolism, or negative regulation of apoptosis execution, spermatogenesis, and immune system, respectively. Microbial abundance and diversity did not differ significantly between males and females. The expression of several host genes on the X chromosome correlated negatively with disease severity. In this way, our analyses identify host genes whose differential expression could aid in the diagnosis of COVID-19 and prediction of its severity *via* non-invasive assay.

## Introduction

As of 21 February 2022, more than 423 million people around the world had been infected by severe acute respiratory syndrome coronavirus 2 (SARS-CoV-2), causing coronavirus disease 2019 (COVID-19), which in turn had resulted in nearly 5.88 million deaths globally (World Health Organization).^1^ Reflecting the heterogeneity of patients, COVID-19 symptoms vary and may include fever, dry cough, fatigue, acute respiratory distress syndrome, and multiple organ failure (Ciaffi et al., 2020; Guan et al., 2020; Huang et al., 2020; Li et al., 2021). Disease severity also varies substantially and is categorized as mild, moderate, severe, or critical (Wei, 2020). Numerous therapeutic approaches against COVID-19 are now in different stages of investigation and application, but SARS-CoV-2 infection still poses a significant threat to global health.

Deepening our understanding of how SARS-CoV-2 affects the host and their microbiota and identifying the predictors of disease progression could substantially improve disease management and allocation of often limited healthcare resources. Studies have linked worse COVID-19 severity to older age (Brodin, 2020), smoking (Smith et al., 2020), male sex (Chakravarty et al., 2020; Conti and Younes, 2020; Gebhard et al., 2020), and comorbidities such as hypertensive disease (Huang et al., 2020). According to the data from Global Health 50/50 (date accessed: 24 February 2022) in the United States, the male/female ratio is 0.89 among those diagnosed with SARS-CoV-2 infection but much higher at 1.27 in cases that eventually lead to death. Association of the male sex with more severe COVID-19 and mortality has also been reported in other countries, including France and China (accessed: 24 August 2021).^2^

A male bias toward more severe disease also occurs in severe acute respiratory syndrome (SARS) and Middle East respiratory syndrome (MERS) (Agrawal et al., 2021). Identifying the molecular basis for this sex bias may help personalize prognosis. The bias in COVID-19 has also been linked to smoking, levels of testosterone and estrogen, and the expression of certain immune-response genes on the X chromosome (Xiao et al., 2020).

To identify the biomarkers of COVID-19 severity, and particularly, the molecular basis of the sex bias of severe disease, we performed meta-transcriptome profiling of nasopharyngeal, oropharyngeal, and anal swabs in a group of COVID-19 patients receiving treatment at our hospital and compared the findings in men vs. women and in patients with varying disease severity.

## Materials and methods

### Patients and data collection

Patients receiving treatment for COVID-19 during a period from 17 January 2020 to 21 March 2020 at our hospital (Zhuhai, China) were recruited into the study, after the study had been approved by the Medical Ethics Committee of the Fifth Affiliated Hospital of Sun Yat-sen University (approval [2020] L019-1). Diagnosis of COVID-19 was based on Chinese National Guidelines (Wei, 2020) and RT-PCR testing using oropharyngeal swab, nasopharyngeal swab, or anal swab. Patients with one or more of the following conditions were excluded from the analysis: (1) aged 80 years or older; (2) uncontrolled preexisting major chronic disorder (e.g., diabetes mellitus, chronic respiratory disease, or cardiovascular diseases); and (3) other active infection in addition to SARS-CoV-2 infection. The samples used for RT-PCR testing and transcriptome profiling were both collected at admission, and the average duration from positive RT-PCR and disease onset was 3.36 days (range: 0–19 days).

Data extracted from electronic medical records included demographic data, medical diagnoses, clinical symptoms, antibiotics exposure, routine blood examination, arterial blood gas analysis, cardiac function, and chest computed tomography. Viral load was determined based on the cycle threshold (Ct) value.

### RNA library construction and sequencing

Microbial RNA was extracted from the 129 samples collected from 125 COVID-19 patients using a QIAamp Viral RNA Mini Kit (Qiagen, 250, Germany) according to the manufacturer’s recommendations. RNA was fragmented, reverse-transcribed, end-repaired, ligated to adapters, and amplified by PCR. The quality of the DNA libraries was checked using an Agilent 2100 system, and libraries of sufficient quality were sequenced in a high-throughput manner on an MGISEQ-2000 platform.

### Sequencing data analysis

Raw sequencing data were filtered using fastp (Chen et al., 2018), and reads that were mapped to the human genome GRCh38 using hisat2 (2.2.1 release) with the default parameters and the mapped reads were removed (Kim et al., 2019). Microbial species were identified based on the analysis of the clean reads by Kraken2 with the self-defined database constructed using the Refseq data^3^ (Wood et al., 2019).

Correlations between microbial taxa and clinical characteristics were tested using Spearman’s correlation analysis. Spearman’s correlation analysis between species was performed using the “hmisc” package in R (v4.0.3). Differences between two taxa were assessed for significance using the Wilcoxon rank sum test (Dutta and Datta, 2016), while differences among three taxa were assessed using the Kruskal-Wallis test (Guo et al., 2013). The Shannon index for microbial α-diversity and Bray-Curtis dissimilarity for β-diversity were calculated using the “vegan” package in R (v4.0.3). Group differences were tested by pairwise permutational multivariate analysis of variance (PERMANOVA). Simple linear regression model and polynomial regression model [using library of “DAAG”in R (v4.0.3)] were used to analyze the relationship between PC1/PC2 factors and the symptoms of mild, moderate, severe, and critical conditions.

Sequences from each sample were assembled *de novo* using SPAdes (version 3.13.0) with only contigs longer than 150 bp. Various k-mer parameters were tested in parallel to identify the optimal one, and the corresponding genome assembly was retained for further analysis. Open reading frames in the assembled genomes were predicted using MetaGeneMark (version 2.10) with default parameters (Zhu et al., 2010). Predicted genes were searched against the Kyoto Encyclopedia of Genes and Genomes (KEGG) database (version 89.1) using DIAMOND (v0.9.21.122) with “diamond blastp, —evalue 0.0000001” and other default parameters in order to predict potential gene function (Kanehisa and Goto, 2000). The ReporterScore method conducted a statistical test on all KOs involved in a certain pathway and used the overall cumulative trend to reflect the changes in the pathway (Patil and Nielsen, 2005). ReporterScore was calculated as follows: the *p*-value of KO was obtained by rank sum test, and the *Z*-value corresponding to the *p*-value was obtained using the inverse normal distribution. The calculation formula is as follows:


$$\mathrm{Zi}=\theta^{\wedge }\,\left(-1\right)\,\left(1-\mathrm{pi}\right)$$

*i* represents the *i*-th KO of a certain pathway, and pi represents the test *p*-value of the *i*-th KO in the group.

*Z*-value calculation of the pathway is as follows:


Zm=Σ⁢Zi/sqrt⁢(k)

Zm represents the *Z*-value of a certain pathway, and *K* represents that there are *K*-th KO annotations to the pathway in this test.

In total, 1,000 permutations of a certain pathway were performed to obtain a random Z-value and correct the Z-value and the true Z-value. The correction formula is as follows:


Zcorrected=(zm-μ⁢k)/σ⁢k

μk is the mean value of 1,000 random passes, and σk is the standard deviation of 1,000 random data.

It is corrected to obey (0,1) standard normal distribution. The corrected *Z*-value is the ReporterScore value. When *Z* < 1.65 or *Z* > 1.65 corresponding to *p* < *0.05*. Not only the difference but also the direction of the difference.

Microbial or human genes differentially expressed between different types of patients were identified using RStudio (version 1.3.959) within the “DEseq2” in R (v4.0.3) (Anders and Huber, 2010), and then, they were depicted on volcano plots using the “ggplot2” package in R (v4.0.3). The gene function enrichment analysis of differentially expressed genes (DEGs) in the host was performed on the Metascape website.^4^ Potential prognostic biomarkers were identified using a trained random forest model and then assessed using receiver operating characteristic curves generated using the library of “randomForest” in R (v4.0.3).

### Other statistical analyses

Continuous data about patient characteristics were reported as mean ± SD and compared between patient groups using the Student’s *t*-test. Categorical data were reported as *n* (%) and compared between groups using Fisher’s exact test. Forward stepwise logistic regression was used to identify the best predictors of mild or severe disease based on univariate and multivariate analyses. All these analyses were performed in SPSS (version 25.0).

## Results

### Patient characteristics associated with coronavirus disease 2019 severity

Our study involved 129 RNA samples from 125 patients diagnosed with coronavirus disease 2019 (COVID-19) during a period from 17 January 2020 to 21 March 2020 at our hospital, of whom 102 (82%) were classified as having mild disease (including 21 or 81 patients with mild or moderate symptoms, respectively) and the remaining 23 (18%) as having severe disease (including 17 or 6 patients with severe or critical symptoms, respectively) due to few samples after categorizing as mild, moderate, severe, or critical, based on Chinese National Guidelines (Wei, 2020). Severe disease occurred more often among men; people aged 52–78 years (mean age 61.91 ± 5.52 years); and patients who experienced fever, shortness of breath, and fatigue (Table 1).

**TABLE 1:** Clinicodemographic characteristics of COVID-19 patients, stratified by disease severity.

Characteristic	Mild disease (*n* = 102)	Severe disease (*n* = 23)	*p*
**Sex**			
Male	46 (45.10)	17 (73.91)	0.013
Female	56 (54.90)	6 (26.09)	
Age at date of first positive test for SARS-CoV-2, yr	37.72 ± 19.57	61.91 ± 5.52	<0.0001
** Symptoms at COVID-19 diagnosis**			
Fever	14 (13.73)	8 (34.78)	0.036
Cough	34 (33.33)	8 (34.78)	0.894
Expectoration	17 (16.67)	3 (13.04)	0.910
Shortness of breath	3 (2.94)	5 (21.74)	0.004
Gastrointestinal symptoms	5 (4.90)	2 (8.70)	0.831
Headache	4 (3.92)	0	1.000
Fatigue	2 (1.96)	4 (17.39)	0.010
Anosmia	4 (3.92)	0	1.000
**Antibiotics exposure preceding admission**			
With	43 (42.16)	23 (100)	<0.0001
Without	59 (57.84)	0	

Values are n (%) or mean ± SD, unless otherwise noted.

Univariate analysis indicated that severe COVID-19 was associated with significantly higher levels of C-reactive protein and D-dimer in blood, as well as the lower arterial partial pressure of oxygen. The two groups of patients did not, however, differ significantly in the other clinical variables analyzed. Forward stepwise multivariate analysis identified only D-dimer concentration as an independent predictor of COVID-19 severity (OR 14.248, 95% CI 2.985–68.005; Supplementary Table 1).

Severe disease was associated with ground-glass opacity on computed tomography of the lungs, which was observed in 63 patients (61.76%) with mild disease but in all 23 (100%) with severe disease. Severe disease was also associated with lymphadenopathy and pleural effusion (Supplementary Table 2).

### Microbiota in three types of swabs from coronavirus disease 2019 patients

Shotgun microbial metagenomic sequencing was performed on 129 RNA samples from 25 anal, 29 nasopharyngeal, and 75 oropharyngeal swabs. The total number of raw RNA sequencing reads was 17,203,683,422, with an average of 124,664,372 reads per sample. After removing low-quality reads, the remaining 17,140,618,365 clean RNA sequencing reads were included, with an average of 124,207,379 reads per sample (Supplementary Table 3).

**FIGURE 1:**
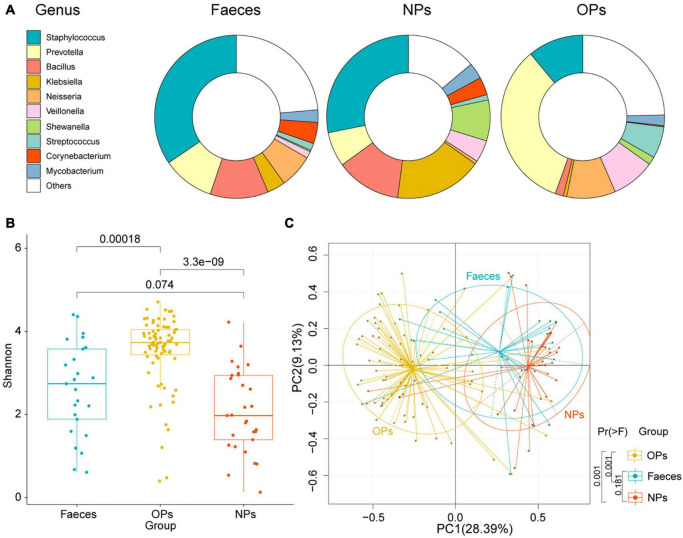
Microbial communities sampled from different tissues of COVID-19 patients. **(A)** Bacterial genera detected in anal (Feces), nasopharyngeal (NPs), and oropharyngeal (OPs) swabs. Only the 10 most abundant genera are shown individually. **(B)** Boxplot of α-diversities in microbial communities detected in anal (blue), nasopharyngeal (red), and oropharyngeal (yellow) swabs. Diversity was measured at the species level based on the Shannon index. N_*Faeces*_ = 25, N_*NPs*_ = 29, N_*OPs*_ = 75. **(C)** Principal component analysis of species-level β-diversity of microbial communities in anal (blue), nasopharyngeal (red), and oropharyngeal (yellow) samples. Group differences were assessed for significance using pairwise permutational multivariate analysis of variance.

The number of detected microbial genera was 393 in anal swabs, 330 in nasopharyngeal swabs, and 1,253 in oropharyngeal swabs; the 10 most abundant genera crosslinked from these swabs were *Staphylococcus, Prevotella, Bacillus*, *Klebsiella*, *Neisseria*, *Veillonella*, *Shewanella*, *Streptococcus*, *Corynebacterium*, and *Mycobacterium* (Figure 1A). These genera together accounted for 76, 86, and 75% of all classified reads in anal, nasopharyngeal, and oropharyngeal swabs, respectively.

Based on the Shannon index of α-diversity, species richness was significantly higher in oropharyngeal swabs than that in the other two types of swabs (*P<0.01* by Shannon-Wiener). The Shannon index of α-diversity did not differ significantly between anal and nasopharyngeal swabs (*P* = 0.074 by Shannon-Wiener) (Figure 1B). Principal component analysis (PCA) detected a significant difference in microbial β-diversity in oropharyngeal than that in the nasopharyngeal and anal swabs (*P* = 0.001 by pairwise permutational multivariate analysis of variance), but no significant difference between nasopharyngeal and anal swabs (*P* = 0.181 by pairwise permutational multivariate analysis of variance; Figure 1C). The data imply that microbial genera were more abundant in oropharyngeal swabs than that in the nasopharyngeal and anal swabs.

Next, we compared the positive rate of detection of severe acute respiratory syndrome coronavirus 2 (SARS-CoV-2) RNA among the three types of swabs. The rates of detection were 0.60 in oropharyngeal swabs, 0.44 in anal swabs, and 0.38 in nasopharyngeal swabs (Supplementary Table 4). Higher number of RNA reads correlated with lower cycle threshold (Ct) in all three swab types from the quantitative RT-PCR (Pearson’s coefficient –0.55733; Supplementary Figure 1 and Supplementary Table 5). This indicates a positive correlation between the number of SARS-CoV-2 reads and viral load.

### Differences in microbial communities between patients with mild or severe coronavirus disease 2019

**FIGURE 2:**
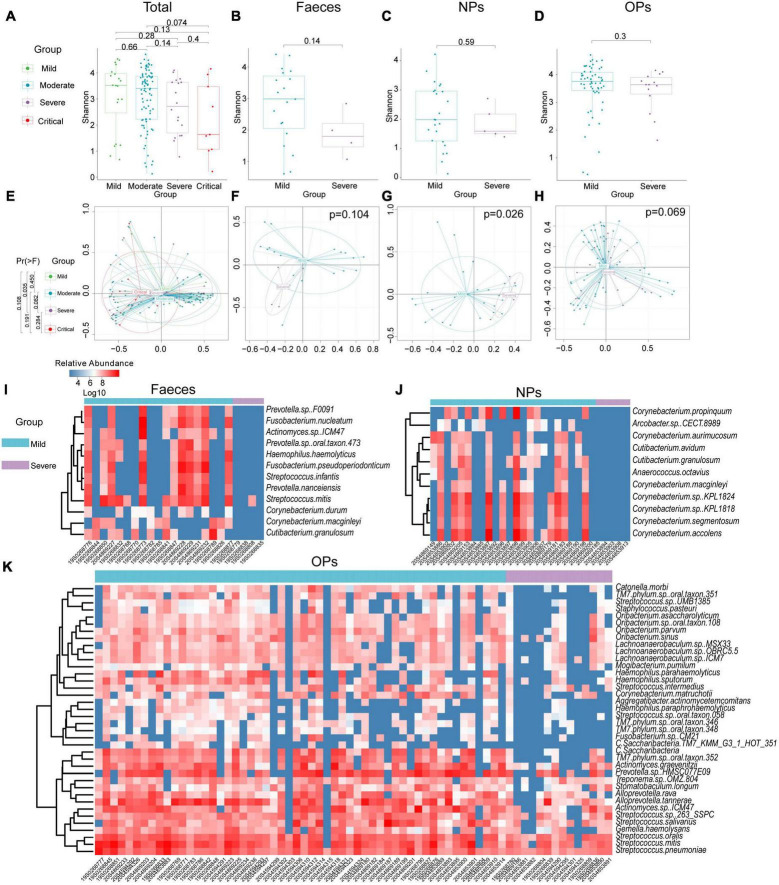
Diversity and composition of microbial communities from patients with COVID-19 of differing severity. **(A–D)** Boxplot of α-diversity of microbial communities from **(A)** all types of swabs (N_*mild*_ = 20, N_*moderate*_ = 81, N_*severe*_ = 17, N_*critical*_ = 6), **(B)** anal swabs (Feces; N_*mild*_ = 19, N_*severe*_ = 4), **(C)** nasopharyngeal swabs (NPs; N_*mild*_ = 24, N_*severe*_ = 5), or **(D)** oropharyngeal swabs (OPs; N_*mild*_ = 58, N_*severe*_ = 14). **(E–H)** Principal component analysis of β-diversity of microbial communities from **(E)** all types of swabs (N_*mild*_ = 20, N_*moderate*_ = 81, N_*severe*_ = 17, N_*critical*_ = 6), **(F)** anal swabs (N_*mild*_ = 19, N_*severe*_ = 4), **(G)** nasopharyngeal swabs (N_*mild*_ = 24, N_*severe*_ = 5), or **(H)** oropharyngeal swabs (N_*mild*_ = 58, N_*severe*_ = 14). Group differences were assessed for significance using pairwise permutational multivariate analysis of variance. **(I–K)** Differences in microbial composition between patients with mild or severe disease based on **(I)** anal swabs (N_*mild*_ = 19, N_*severe*_ = 4), **(J)** nasopharyngeal swabs (N_*mild*_ = 24, N_*severe*_ = 5) or **(K)** oropharyngeal swabs (N_*mild*_ = 58, N_*severe*_ = 14).

After excluding 4 patients who were asymptomatic, of whom were classified as having mild disease, the remaining 124 RNA samples of 121 patients were categorized as having mild (*n* = 20), moderate (81), severe (17), or critical (6) disease (Supplementary Table 6). The species-level α-diversity of microbial communities was lower in patients with increasing disease severity (Figure 2A). Additionally, this difference in the diversity of microbial communities was confirmed by PCA, and microbial diversity ordered changed slowly with the aggravation of symptoms in COVID-19 patients (polynomial regression model, PC1 *P* = 0.0564; Figure 2E and Supplementary Figure 2). To confirm the findings of the above analyses, patients with mild or moderate symptoms were grouped together (mild disease group), as were patients with severe or critical symptoms (severe disease group). Similarly, we found that species-level α-diversity declined with increasing disease severity for all three types of swabs (Figures 2B–D and Supplementary Table 7). According to PCA, the difference in microbial diversity with more severe disease was significant only for nasopharyngeal swabs (*P* = 0.026; Figures 2F–H).

In the analysis using anal swabs, severe disease was associated with a reduced abundance of 12 bacterial species across four genera, namely, *Corynebacterium*, *Fusobacterium*, *Prevotella*, and *Streptococcus*, as well as an increased abundance of 19 bacterial species across three genera, namely, *Bacillus*, *Chryseobacterium*, and *Enhydrobacter* (Figure 2I).

In the analysis using nasopharyngeal swabs, severe disease was associated with a reduced abundance of 11 bacterial species in either the *Corynebacterium* or *Cutibacterium* genus and with an increased abundance of 20 species in either the *Bacillus* or *Vibrio* genus (Figure 2J).

In the analysis using oropharyngeal swabs, severe disease was associated with a reduced abundance of 38 bacterial species across seven genera, namely, *Actinomyces*, *Alloprevotella*, *Haemophilus*, *Lachnoanaerobaculum*, *Oribacterium*, *Streptococcus*, and *Candidatus*, and an increased abundance of 38 bacterial species across four genera, namely, *Campylobacter*, *Chitinophaga*, *Mucilaginibacter*, and *Prevotella* (Figure 2K).

### Differences in the expression of patient genes between mild and severe coronavirus disease 2019

First, we profiled the expression of human genes based on RNA that we were able to map to the reference human genome, and we compared the profiles between patients with mild, moderate, severe, or critical disease, as well as across the three swab types. Spearman’s correlation analysis was performed between host gene expression and severity of COVID-19 patients. When the Spearman’s correlation coefficient was more than 0.4, the expression of many genes (*P* < 0.05 by Fisher’s test) involved in transport and catabolism (endocytosis), sensory system (olfactory transduction), transcription (RNA polymerase), and immune system (cytosolic DNA-sensing pathway) was correlated negatively with disease severity in anal swabs (Supplementary Table 8.1). Conversely, the expression of many genes (*P* < 0.05 by Fisher’s test) involved in human diseases of the endocrine and metabolic diseases (Cushing syndrome), infectious diseases (bacterial: *staphylococcus aureus* infection), and diseases of the endocrine system (aldosterone synthesis and secretion, and cortisol synthesis and secretion) was correlated positively with disease severity (Supplementary Table 8.2). In nasopharyngeal swabs, severe disease correlated with the downregulation of many genes (*P* < 0.05 by Fisher’s test) involved in the immune system, responses to infectious disease (bacterial, viral), and metabolism (carbohydrate, cofactors, vitamins, and nucleotide; Supplementary Table 8.3). However, gene expression profiles derived from oropharyngeal swabs did not correlate with disease severity.

**FIGURE 3:**
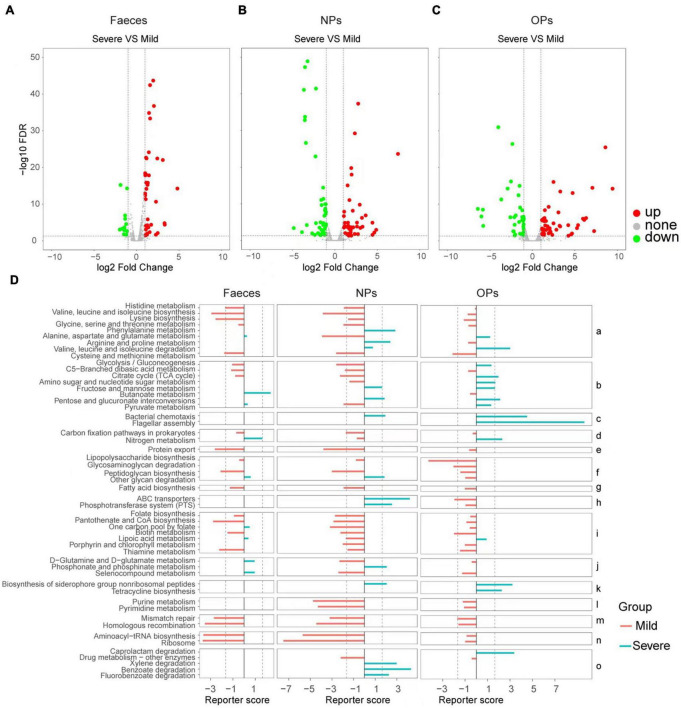
Comparison of human and microbial gene expression in patients with mild or severe COVID-19 and potential functional implications. **(A–C)** Volcano plots of differences in human gene expression between patients with severe vs. mild disease, based on **(A)** anal swabs (feces; N_*mild*_ = 19, N_*severe*_ = 4), **(B)** nasopharyngeal swabs (NPs; N_*mild*_ = 24, N_*severe*_ = 5) or **(C)** oropharyngeal swabs (OPs; N_*mild*_ = 58, N_*severe*_ = 14). Genes whose expression did not differ significantly between the two patient groups are shown in gray; genes with significant upregulation are shown in red and downregulation are shown in green. The dotted lines of X axes indicate fold change = ± 1.2x, the dotted lines of Y axes indicate FDR > 0.05. **(D)** Functional enrichment of microbial genes differentially expressed between patients with mild (red) or severe disease (cyan), based on Kyoto Encyclopedia of Genes and Genomes pathways. Results are shown separately for the three swab types. Pathways are grouped according to the following functional categories: (a) amino acid metabolism; (b) carbohydrate metabolism; (c) cell motility; (d) energy metabolism; (e) protein folding, sorting, and degradation; (f) glycan biosynthesis and metabolism; (g) lipid metabolism; (h) membrane transport; (i) metabolism of cofactors and vitamins; (j) metabolism of other amino acids; (k) metabolism of terpenoids and polyketides; (l) nucleotide metabolism; (m) replication and repair; (n) translation; and (o) xenobiotic biodegradation and metabolism. The dotted lines indicate fold change = ± 1.65.

Next, we compared human gene expression between mild (patients with mild or moderate symptoms) and severe (patients with severe or critical symptoms) groups in order to identify functional pathways, based on Gene Ontology terms, that were upregulated or downregulated in severe COVID-19. In anal swabs, 36 genes were upregulated, and 48 genes were downregulated in the severe group. The Gene Ontology analysis enriched upregulated genes in the negative regulation of apoptosis execution and in spermatogenesis and downregulated genes in protein modification by small protein removal (Figure 3A and Supplementary Table 9.1). In nasopharyngeal swabs, 43 genes were upregulated and 42 genes were downregulated in the severe group. The Gene Ontology analysis enriched upregulated genes in negative regulation of apoptosis execution and in spermatogenesis, and downregulated genes in negative regulation of cellular catabolism, nucleosome assembly, and defense responses to the bacterium (Figure 3B and Supplementary Table 9.2). In oropharyngeal swabs, 35 genes were upregulated in severe disease, and they were enriched in protein modification by small protein removal, estrogen-dependent gene expression, and cytokine signaling in the immune system (Figure 3C and Supplementary Table 9.3).

### Differences in the expression of microbial genes between mild and severe coronavirus disease 2019

Analogously as with patient genes, we profiled the expression of microbial genes in the three types of swabs, and we compared the profiles and potential functional implications, based on Kyoto Encyclopedia of Genes and Genomes pathways enriched by Reporter Score analysis, respectively, between patients with mild or severe COVID-19. Microbes detected in patients with severe disease were enriched for genes involved in the metabolism of phenylalanine, arginine, and proline, while microbes from patients with mild disease were enriched for genes involved in the metabolism of other amino acids, such as cysteine, methionine, histidine, valine, leucine, isoleucine, lysine, alanine, aspartate, and glutamate.

Additionally, mild disease was associated with significant enrichment of genes involved in the following pathways: protein folding, sorting, and degradation (export); metabolism of cofactors and vitamins, including folate, pantothenate, CoA, biotin, and thiamine; metabolism of purines and pyrimidines; DNA mismatch repair and homologous recombination; and aminoacyl-tRNA biosynthesis and ribosomes. This suggests that the microbiome in patients with mild COVID-19 shows higher levels of translation initiation, protein folding, and protein modification than the microbiome in patients with severe disease.

Conversely, severe disease was associated with significant enrichment of genes involved in chemotaxis and flagellar assembly; metabolism of terpenoids and polyketides, including biosynthesis of siderophore groups; and xenobiotic biodegradation and metabolism, including degradation of caprolactam, xylene, benzoate, and fluorobenzoate. This suggests that the microbiome in patients with severe COVID-19 shows more active drug metabolism than the microbiome in patients with mild disease (Figure 3D and Supplementary Figure 3).

### Sex differences in microbial diversity and human gene expression between mild and severe coronavirus disease 2019

**FIGURE 4:**
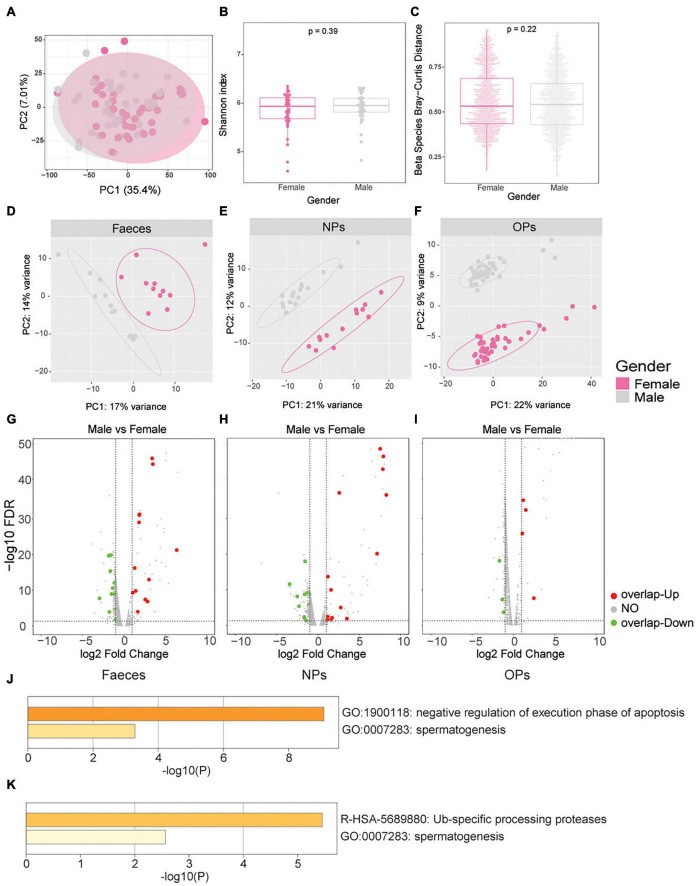
Sex differences in microbiome characteristics and human gene expression, and association with COVID-19 severity. **(A–C)** Differences in microbial gene expression between male patients of any disease severity (gray) and female patients of any disease severity (pink). **(A)** Principal component analysis of microbial β-diversity. **(B)** Boxplot of α-diversity, based on the Shannon index. **(C)** Boxplot of β-diversity, based on the Bray-Curtis distance. **(D–F)** Principal component analysis of human gene expression between males (gray) and females (pink) based on the three swab types, including **(D)** anal swabs (feces), **(E)** nasopharyngeal swabs (NPs), **(F)** oropharyngeal swabs (OPs). **(G–I)** Volcano plots of differences in human gene expression between males and females based on the three swab types, including **(G)** anal swabs, **(H)** nasopharyngeal swabs, and **(I)** oropharyngeal swabs. Genes whose expression did not differ significantly between the groups of mild vs. female or no overlap genes of severe vs. mild are shown in gray; genes with significant upregulation in male vs. female overlap genes of severe vs. mild are shown in red and genes with significant downregulation in male vs. female overlap genes of severe vs. mild are shown in green. The dotted lines of X axes indicate fold change = ± 1.2x, the dotted lines of Y axes indicate FDR > 0.05. **(J,K)** These genes highly expressed in male patients and in patients with severe disease were enriched by Metascape, including **(J)** anal swabs and **(K)** nasopharyngeal swabs.

We examined whether microbiome characteristics or disease severity differed with sex in our patient samples. No significant differences in microbial composition were identified between male and female patients in either PCA (Figure 4A) or α-diversity and β-diversity testing (*P_α_* = 0.39, *P_β_* = 0.22; Figures 4B,C). As expected, the human transcriptome differs significantly between the male and female patients in all three swab types (Figures 4D–F). Among the top 200 genes differentially expressed between the male and female patients, 178 (54%) patients were detected in at least two of the three types of swabs, and 94 (28.66%) patients were detected in all three types of swabs (Supplementary Figure 4). The top 200 genes differentially expressed between the male and female patients were enriched in the Gene Ontology processes of gamete generation and gonadal mesoderm development (Supplementary Figure 5 and Supplementary Tables 9.1–9.3), associated with the sex hormones. Meanwhile, the sex bias toward more severe disease occurred in SARS-CoV-2 infection according to published reports (Chakravarty et al., 2020; Conti and Younes, 2020). Hence, we hypothesize that these differentially expressed genes (DEGs) of sex differences might be correlated with COVID-19 severity.

Among the genes differentially expressed between the male and female patients, we next identified several genes that were also differentially expressed between patients with mild or severe disease. In anal swabs, 21 genes were highly expressed both in male patients and in patients with severe disease; these genes were enriched in processes involving negative regulation of apoptosis execution and spermatogenesis (Figures 4G,J and Supplementary Table 10.1). In nasopharyngeal swabs, 16 genes were highly expressed both in male patients and in patients with severe disease; these genes were enriched in processes involving ubiquitin-specific processing proteases and spermatogenesis (Figures 4H,K and Supplementary Table 10.2). In oropharyngeal swabs, seven genes were highly expressed in both males and patients with severe disease, but none were significantly enriched in any specific Gene Ontology term (Figure 4I and Supplementary Table 10.3).

### Biomarker signatures predict the severity of coronavirus disease 2019

**FIGURE 5:**
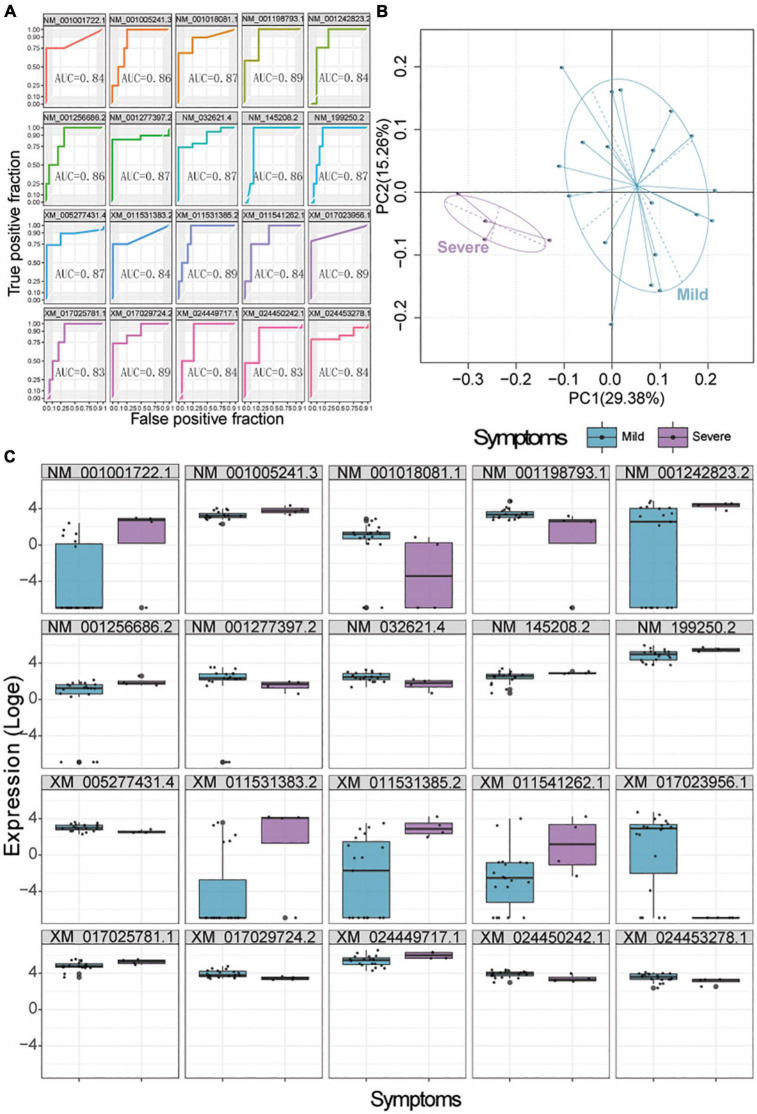
Biomarker signatures predict COVID-19 severity in anal swabs. **(A)** Assessment of the ability of the top 20 genes selected by a random forest model to predict mild or severe disease. AUC, area under the receiver operating characteristic curve. **(B)** Principle component analysis showing the ability of the selected 20 genes to cluster patients with mild (blue) or severe (purple) disease. **(C)** Expression of the selected 20 genes in patients with mild (blue) or severe (purple) disease.

We used a random forest model to identify which genes, among those DEGs between patients with mild or severe disease in all three types of swabs, might predict the severity of COVID-19. Top 20 DEGs associated with disease severity were selected as candidate biomarkers in each type of swab (Supplementary Tables 11.1–11.3). Then, the predictive ability of these biomarkers was assessed using receiver operating characteristic curves and PCA. In anal swabs, the area under receiver operating curves of these markers ranged from 0.83 to 0.89 (Figure 5A), and clustering based on gene expression or PCA showed complete separation of patients with mild vs. severe disease (Figures 5B,C). In nasopharyngeal swabs, the area under receiver operating curves of these markers ranged from 0.79 to 0.92, and clustering based on gene expression or PCA showed complete separation of patients with mild vs. severe disease (Supplementary Figures 6A–C). Similar results were observed for biomarkers in oropharyngeal swabs, which showed areas under the curve ranging from 0.70 to 0.78 (Supplementary Figures 7A–C). Altogether, we identified 9 genes in anal swabs, 20 genes in oropharyngeal swabs, and 19 genes in nasopharyngeal swabs—or 19 genes across all three types of swabs—whose expression negatively correlated with disease severity (Supplementary Table 11.4).

To benchmark these biomarkers, we used the D-dimer concentration identified to be an independent predictor of severity disease in our samples by the forward stepwise logistic regression as a standard of measurement. We found that the area under the curve for D-dimer, 0.881 (Supplementary Figure 8), was comparable to the areas for our biomarkers.

The number of biomarkers on the X chromosome for severe disease was 4 in anal swabs, 14 in oropharyngeal swabs, 4 in nasopharyngeal swabs, and 9 across all three types of swabs. In anal swabs, we found that two genes located on the X chromosome were positively correlated with disease severity, namely, CT47A8 and CT47A4, both of which belong to the cancer/testis antigen family (Supplementary Table 11.1). In oropharyngeal swabs, majority of the biomarkers (14/20) located on the X chromosome were negatively correlated with the severity of symptoms, including TCP11 × 2 (t-complex 11 family member), which is a potential determinant of the sperm morphology (Liu et al., 2011), and two immune-related genes, namely, GAGE12G (G antigen) and MAGED2 (MAGE family member) (Supplementary Table 11.2; Sang et al., 2011). Meanwhile, we found that XAGE1A, a member of the X antigen family member, was negatively correlated with disease severity in nasopharyngeal swabs (Supplementary Table 11.3). In altogether of three types of swabs, we found that some immunity-related genes located on the X chromosome were negatively correlated with the severity of symptoms, such as GAGE12F (G antigen 12F), TNMD (tenomodulin), MAGEA6 (MAGE family member), and MAGED2 (MAGE family member) (Supplementary Table 11.4; Tanzarella et al., 1999; Tolppanen et al., 2008; Vujanovic et al., 2014). This provides further evidence that COVID-19 severity shows a sex bias.

## Discussion

Results from this study suggest that the severity of COVID-19 correlates negatively with the diversity of microbiota in the three most frequently used types of swabs in the diagnosis. Part of the reason for this finding may be earlier/preventive use of antibiotics among patients with severe disease, who would have been given such drugs to prevent worsening of symptoms. Consistent with this finding, many genes involved in drug metabolism were upregulated by the microbiome on the swab samples from patients with severe disease, whereas the microbiome on swabs from patients with mild disease strongly expressed many genes involved in other types of metabolism.

The most abundant microbial genera in oropharyngeal swabs from COVID-19 patients were *Prevotella* (33.51%), *Staphylococcus* (11.02%), *Neisseria* (9.66%), *Veillonella* (8.63%), *Streptococcus* (6.29%), *Haemophilus* (3.93%), *Fusobacterium* (3.06%), *Mycobacterium* (2.11%), and *Actinomyces* (1.78%) by RNA sequencing. These findings differed from the results of previous published studies based on DNA sequencing in COVID-19 patients [*Veillonella* (22.7%), *Streptococcus* (20.3%), *Prevotella* (7.1%), *Acinetobacter* (5%), *Megasphaera* (4.21%), *Actinomyces* (4.19%), *Atopobium* (3.65%), *Klebsiella* (3.25%), and *Solobacterium* (2.07%)] (Ma S. et al., 2021). Such a discrepancy may reflect the fact that transcriptional profiling is complementary to the data on DNA sequencing due to its advantage of revealing active microbial genera. This study also showed a distinct difference in the microbial composition between oropharyngeal swabs and anal swabs (or nasopharyngeal swabs) *via* RNA sequencing. We speculate that such a difference is due to the open oral environment, in which microbial composition changes slightly in patients. Hence, the change in microbial composition in the group of oropharyngeal swabs is not sufficient to represent in patients infected with COVID-19. It is necessary to observe the microbial composition in various parts of patients infected with COVID-19.

Microbiome did not differ significantly between male and female COVID-19 patients. As expected, the genes that were differentially expressed between the two sexes were enriched in pathways related to the production of sex hormones, i.e., gamete generation and gonadal mesoderm development. Signatures containing the top 20 differentially expressed genes in anal and nasopharyngeal swabs were able to distinguish patients with mild vs. severe disease with areas under the receiver operating characteristic curves of 0.79–0.92. The area under the curve was somewhat lower at 0.70–0.78 for oropharyngeal swabs. These results suggest that the markers detected by anal and nasopharyngeal swabs are more suitable for predicting symptom severity than that by oropharyngeal swabs. Whatever, the transcriptome profiling of COVID-19 patients may be important for predicting prognosis.

Majority of the biomarkers located on the X chromosome correlated negatively with COVID-19 severity based on our data. SARS-CoV-2 could invade the testis by disrupting blood-testis barrier due to inflammatory infiltration and innate immune homeostasis damage (Peirouvi et al., 2021; Zafar et al., 2021). Mechanistically, SARS-CoV-2 infection might increase inflammatory cytokines, including TNF-α, IL-1β, IL-6, and CCL2. Entry of the virus into target testicular cells depends on the recognition of angiotensin-converting enzyme 2 (ACE2) receptor by the viral S protein (Liu et al., 2020; Tian and Zhou, 2021). SARS-CoV-2 virus eventually resulted in potential germ cells loss, spermatogenesis, and male reproductive system damage (Ma X. et al., 2021). In this study, several genes highly expressed in male patients with severe disease were enriched in spermatogenesis-related pathway in anal and nasopharyngeal swabs, supporting the potential injury of the male reproductive system by SARS-CoV-2. Several biomarkers located on the X chromosome for disease severity in this data are immune-related, potential determinants of the sperm morphology, or belonging to the testis antigen family. We speculate that the high expression of these immune-related genes can alleviate the aggravation of COVID-19 symptom severity, such as GAGE12F, TNMD, MAGEA6, MAGED2, and XAGE1A (Tanzarella et al., 1999; Tolppanen et al., 2008; Vujanovic et al., 2014). These findings are potentially relevant since SARS-CoV-2 has been reported to damage spermatogenesis *via* autoimmune response (Yang et al., 2020; Zheng et al., 2020). The relatively high expression of these genes is also consistent with the less vulnerability of women to severe COVID-19.

This study has several important limitations. First, the sample size is relatively small. Also, the assay was conducted only in the three types of swab samples, and not in urine, bile, alveolar lavage fluid, and blood. Additionally, samples should be analyzed at different disease stages in the same patient. More importantly, large cohort studies are needed in the future to verify the preliminary findings in this study.

In conclusion, the biomarker candidates identified in this study, if confirmed in follow-up studies, may aid in the diagnosis of COVID-19 and in the stratification of patients by risk of severe disease. In this way, transcriptional profiling of patients may facilitate their personalized treatment.

## Data Availability Statement

The datasets presented in this study can be found in online repositories (CNGB Sequence Archive of China National GeneBank DataBase), under accession number CNP0002879 (https://db.cngb.org/search/project/CNP0002879/).

## Ethics statement

The study had been approved by the Medical Ethics Committee of the Fifth Affiliated Hospital of Sun Yat-sen University [approval (2020) L019-1]. The patients/participants provided their written informed consent to participate in this study.

## Author contributions

HRG and JMMi performed the experiments. HL, BL, ZZ, XYHu, LL, and TW performed the bioinformatics analyses. XYHe, JC, YZ, JH, LC, DW, JS, NZ, WH, JZ, and ZS performed the experiments and analyzed the data. HRG, JMMi, HL, and BL wrote the manuscript. PP, JMMa, JJMa, XJH, and HGu designed the study and supervised the project. All authors contributed to the article and approved the submitted version.

## Footnotes

1
https://covid19.who.int/

2
https://globalhealth5050.org/covid19/Date

3
ftp://ncbi.nlm.nih.gov/refseq/release/

4
https://metascape.org/gp/index.html#/main/step1
