# Supplementary material for: Microbial and human transcriptional profiling of coronavirus disease 2019 patients: Potential predictors of disease severity
Source: Front Microbiol. 2022 Sep 2;13:959433. doi: 10.3389/fmicb.2022.959433 (PMC9479730; doi:10.3389/fmicb.2022.959433)
Supplement: Supplementary file 1 [file Data_Sheet_1.pdf]

SUPPLEMENTAL INFORMATION FOR:

Microbial and human transcriptional profiling of COVID-19 patients: potential predictors of disease severity

This document contains the following:

Additional Tables of Contents

Page 3-4: Supplemental Table 1. Identication of factors associated with risk of severe COVID-19 disease

Page 5: Supplemental Table 2. Summary of chest computed tomography findings for patients with mild or severe COVID-19

Page 5-10: Supplemental Table 3. Description of RNA sequencing data

Page 11-16: Supplemental Table 4. Read numbers and detection rate of SARS-CoV-2

Page 17-18: Supplemental Table 5. Threshold cycle values for SARS-CoV-2

Page 19-23: Supplemental Table 6. Patient sex and symptoms

Page 24: Supplemental Table 7.1. Species-level α-diversity declined with increasing disease severity in Faeces

Page 25-26: Supplemental Table 7.2. Species-level α-diversity declined with increasing disease severity in NPs

Page 26-29: Supplemental Table 7.3. Species-level α-diversity declined with increasing disease severity in OPs

Page 30-34: Supplemental Table 8.1. Human genes whose expression correlated negatively with COVID-19 severity in Faeces used the spearman analysis

Page 35-37: Supplemental Table 8.2. Human genes whose expression correlated positively with COVID-19 severity in Faeces used the spearman analysis

Page 38-53: Supplemental Table 8.3. Human genes whose expression correlated negatively with COVID-19 severity in NPs used the spearman analysis

Page 54-76: Supplemental Table 9.1. Human genes differentially expressed in severe disease relative to mild disease in Faeces

Page 76-105: Supplemental Table 9.2. Human genes differentially expressed in severe disease relative to mild disease in NPs

Page 106-120: Supplemental Table 9.3. Human genes differentially expressed in severe disease relative to mild disease in OPs

Page 120-122: Supplemental Table 10.1. Human genes differing in expression between severe and mild disease, or between males and females in Faeces

Page 123-126: Supplemental Table 10.2. Human genes differing in expression between severe and mild disease, or between males and females in NPs

Page 126-127: Supplemental Table 10.3. Human genes differing in expression between severe and mild disease, or between males and females in OPs
Page 128: Supplemental Table 11.1. Twenty genes differentially expressed between patients with mild or severe disease in Faeces
Page 129: Supplemental Table 11.2. Twenty genes differentially expressed between patients with mild or severe disease in OPs
Page 130: Supplemental Table 11.3. Twenty genes differentially expressed between patients with mild or severe disease in NPs
Page 131: Supplemental Table 11.4. Twenty genes differentially expressed between patients with mild or severe disease in all three types of swabs

Additional Figures of Contents

Page 132: Supplemental Figure 1. Correlation between the cycle threshold value in quantitative RT-PCR and Reads detected for SARS-CoV-2 virus DNA
Page 133: Supplemental Figure 2. The longitudinal analysis of PCA
Page 134: Supplemental Figure 3. The coverage of functional enrichment of microbial genes differentially expressed between patients with mild or severe disease
Page 135: Supplemental Figure 4. Venn diagram depicting overlap among 200 genes with the greatest differential expression between males and females
Page 136: Supplemental Figure 5. Enrichment in Gene Ontology terms of 200 genes with the greatest differential expression between patients with mild or severe COVID-19
Page 136-138: Supplemental Figure 6. Biomarker signatures predict COVID-19 severity in nasopharyngeal swabs
Page 138-140: Supplemental Figure 7. Biomarker signatures predict COVID-19 severity in oropharyngeal swabs
Page 140-141: Supplemental Figure 8. Receiver operating characteristic curve analysis to assess the ability of a D-dimer concentration determined by random forest modeling

SUPPLEMENTAL TABLES

Table S1. Identication of factors associated with risk of severe COVID-19 disease

Variable	Level in patients with		Stratification	Univariate analysis			Multivariate analysis		
	Mild COVID-19 (n = 102)	Severe COVID-19 (n = 23)		OR	95% Cl	*p* value	OR	95% Cl	*p* value
Leukocyte count (10^9/L)	8.19±7.12	7.25±8.63	≥3.5 VS. ＜ 3.5			*NS*			*NS*
Neutrophil count (10^9/L)	5.99±6.75	3.03±1.63	＞6.3 VS. ≤6.3			*0.105*			*0.134*
Lymphocyte count (10^9/L)	1.44±0.66	1.95±0.78	≥1.1 VS. ＜ 1.1			*0.297*			*0.814*
Hemoglobin (g/L)	129.65±22.69	135.16±16.11	≥130 VS. ＜ 130			*0.561*			*0.352*
Platelet count (10^9/L)	184.22±44.87	235.76±61.59	≥125 VS. ＜ 125			*0.129*			*NS*
C-reactive protein (mg/L)	26.04±34.61	7.17±19.48	＞5 VS.≤5	4.188	1.619-10.837	*0.003*			*0.462*
Alanine aminotransferase (U/L)	23.61±13.18	23.27±17.12	＞50 VS.≤50			*0.564*			*NS*
Aspartate aminotransferase (U/L)	22.76±6.98	22.19±9.44	＞40 VS.≤40			*NS*			*NS*
Albumin (g/L)	41.21±8.69	40.40±3.33	＞55 VS.≤55			*0.595*			*NS*
Serum creatinine (umol/L)	76.81±31.69	62.82±24.61	＞111 VS. ≤111			*0.346*			*NS*
Blood urea nitrogen (mmol/L)	4.64±1.99	3.68±1.47	＞9.5 VS.			*0.129*			*NS*

			≤9.5						
Glucose (mmol/L)	6.69±3.67	5.39±2.01	＞6.1 VS. ≤6.1			*0.273*			*0.851*
Creatine kinase (U/L)	61.70±30.88	72.60±41.42	＞310 VS. ≤310			*NS*			*NS*
Troponin I (ug/L)	＜0.01	＜0.01	＞0.01 VS. ≤0.01			*NS*			*NS*
Arterial partial pressure of oxygen (mmHg)	87.29±20.48	105.23±41.13	≥83 VS.＜83	5.407	2.054-14.234	*0.001*			*0.185*
Arterial partial pressure of carbon dioxide (mmHg)	38.43±3.74	39.77±4.58	＞45 VS.≤45			*0.318*			*0.37*
D-dimer (ng/ml)	352.91±410.53	137.49±178.14	＞243 VS. ≤243	9	3.203-25.285	＜ *0.0001*	14.248	2.985-68.005	*0.001*

Values are shown as mean ± SD, unless otherwise noted.
CI, confidence interval; NS, not significant; OR, odds ratio

Table S2. Summary of chest computed tomography findings for patients with mild or severe COVID-19

Variable	Mild disease (n = 102)	Severe disease (n = 23)	*p* value
Ground-glass opacity	63 (61.76)	23 (100%)	*< 0.0001*
Lymphadenopathy	0	5 (21.74%)	*< 0.0001*
Pleural effusion	0	3 (13.04%)	*0.006*

Values are n (%) or mean ± SD, unless otherwise noted.

Table S3. Description of RNA sequencing data

ID	No. of raw reads	No. of clean reads	No. of filtered_human_reads	COVID-19 severity	Tissue
S001	152,053,245	151,903,633	1,932,821	Moderate	Anal
S002	164,085,531	163,927,083	5,197,952	Moderate	Anal
S003	147,446,275	147,344,110	49,063,877	Moderate	Anal
S004	120,423,768	120,294,496	2,040,681	Mild	Anal
S005	100,623,444	100,209,988	3,185,077	Critical	Anal
S006	190,963,227	190,682,814	4,556,872	Moderate	Anal
S007	116,214,641	116,149,945	1,067,415	Moderate	Anal
S008	127,633,740	127,455,320	2,308,768	Moderate	Anal
S009	112,609,454	112,547,108	2,371,590	Sever	Anal
S010	157,634,722	157,387,043	2,944,732	Moderate	Anal
S011	160,045,161	159,723,908	2,450,188	Asymptomatic	Anal
S012	98,722,549	98,142,931	3,444,385	Mild	Anal

S013	123,036,559	122,881,526	1,513,375	Sever	Anal
S014	174,765,470	174,549,798	3,244,459	Sever	Anal
S015	58,555,295	58,448,243	3,497,967	Moderate	Anal
S016	108,102,707	107,958,927	1,438,750	Mild	Anal
S017	99,347,929	99,224,584	2,978,874	Moderate	Anal
S018	97,621,539	97,471,342	3,180,010	Mild	Anal
S019	79,681,079	79,613,374	12,367,251	Moderate	Anal
S020	97,634,687	97,548,592	1,854,153	Mild	Anal
S021	134,692,592	134,625,738	4,518,965	Moderate	Anal
S022	90,630,160	90,584,399	2,160,019	Moderate	Anal
S023	131,168,301	131,096,202	2,083,991	Normal	Anal
S024	145,593,201	145,496,927	1,967,277	Moderate	Anal
S025	198,565,387	198,410,486	9,453,956	Moderate	Anal
S026	180,928,747	180,748,457	35,718,464	Moderate	Nasopharyngeal
S027	203,793,607	203,568,795	3,040,872	Moderate	Nasopharyngeal
S028	119,566,786	119,444,576	6,140,204	Mild	Nasopharyngeal
S029	100,127,304	99,910,809	2,022,149	Moderate	Nasopharyngeal
S030	120,501,618	120,381,695	11,224,904	Moderate	Nasopharyngeal
S031	160,636,627	160,507,645	3,184,197	Sever	Nasopharyngeal
S032	177,938,241	177,804,880	3,171,793	Moderate	Nasopharyngeal
S033	129,835,952	129,728,794	2,245,258	Moderate	Nasopharyngeal
S034	123,242,637	122,975,543	4,606,044	Moderate	Nasopharyngeal
S035	146,905,682	146,698,602	2,612,776	Moderate	Nasopharyngeal
S036	133,991,226	133,912,345	2,828,706	Mild	Nasopharyngeal
S037	133,020,314	132,764,117	1,753,405	Sever	Nasopharyngeal
S038	181,114,340	180,779,057	5,275,391	Moderate	Nasopharyngeal

S039	147,366,607	147,111,115	3,026,761	Moderate	Nasopharyngeal
S040	132,848,205	132,618,168	7,599,724	Moderate	Nasopharyngeal
S041	125,068,643	124,859,698	1,839,408	Sever	Nasopharyngeal
S042	118,886,776	118,674,213	2,487,636	Sever	Nasopharyngeal
S043	238,686,278	236,145,308	9,760,786	Mild	Nasopharyngeal
S044	272,410,017	271,635,447	2,847,947	Moderate	Nasopharyngeal
S045	134,345,007	134,205,107	6,317,527	Moderate	Nasopharyngeal
S046	148,359,054	148,261,680	3,581,364	Moderate	Nasopharyngeal
S047	119,937,866	119,802,543	1,858,986	Moderate	Nasopharyngeal
S048	140,275,265	140,175,549	2,032,173	Critical	Nasopharyngeal
S049	87,719,189	87,616,538	1,372,210	Moderate	Nasopharyngeal
S050	93,546,068	90,571,190	3,071,234	Moderate	Nasopharyngeal
S051	119,932,848	119,499,345	3,122,354	Moderate	Nasopharyngeal
S052	81,107,306	81,005,712	1,279,842	Moderate	Nasopharyngeal
S053	138,849,263	138,786,987	1,770,440	Mild	Nasopharyngeal
S054	156,493,449	156,302,197	6,052,666	Mild	Nasopharyngeal
S055	191,777,759	191,631,473	51,013,992	Moderate	Oropharyngeal
S056	137,419,548	137,399,018	109,600,130	Moderate	Oropharyngeal
S057	122,199,537	122,143,218	1,326,704	Moderate	Oropharyngeal
S058	106,182,243	106,113,594	5,609,324	Mild	Oropharyngeal
S059	188,909,576	188,798,726	75,157,315	Critical	Oropharyngeal
S060	163,241,809	163,148,519	35,573,308	Moderate	Oropharyngeal
S061	148,825,106	148,716,113	25,068,640	Moderate	Oropharyngeal
S062	154,650,199	154,404,558	32,765,176	Moderate	Oropharyngeal
S063	52,560,652	52,488,175	19,283,582	Sever	Oropharyngeal
S064	138,432,180	138,337,879	2,570,634	Normal	Oropharyngeal

S065	79,966,326	79,834,586	5,150,392	Sever	Oropharyngeal
S066	144,952,361	144,731,771	56,366,175	Moderate	Oropharyngeal
S067	98,548,308	98,323,208	13,072,737	Asymptomatic	Oropharyngeal
S068	171,241,537	170,950,061	14,543,513	Mild	Oropharyngeal
S069	124,176,517	123,956,229	7,047,520	Sever	Oropharyngeal
S070	90,582,019	90,459,069	70,964,101	Sever	Oropharyngeal
S071	89,127,437	88,988,862	27,006,037	Moderate	Oropharyngeal
S072	117,955,578	117,778,345	18,592,002	Mild	Oropharyngeal
S073	68,831,892	68,730,736	23,586,358	Moderate	Oropharyngeal
S074	126,964,555	126,780,526	45,261,646	Mild	Oropharyngeal
S075	116,642,545	116,572,904	15,994,167	Moderate	Oropharyngeal
S076	135,256,097	135,062,348	84,537,539	Sever	Oropharyngeal
S077	156,484,319	153,984,942	36,110,506	Moderate	Oropharyngeal
S078	146,325,133	145,944,977	6,827,200	Mild	Oropharyngeal
S079	110,189,042	109,984,847	58,658,224	Moderate	Oropharyngeal
S080	153,768,300	153,238,865	68,808,675	Sever	Oropharyngeal
S081	104,946,820	104,746,626	100,869,726	Moderate	Oropharyngeal
S082	135,669,090	135,255,967	11,351,401	Moderate	Oropharyngeal
S083	144,063,181	143,895,628	36,375,415	Sever	Oropharyngeal
S084	150,678,140	150,564,348	10,080,393	Moderate	Oropharyngeal
S085	102,591,992	100,558,653	1,877,858	Moderate	Oropharyngeal
S086	119,037,200	118,359,057	18,055,040	Moderate	Oropharyngeal
S087	126,428,917	103,272,342	3,920,326	Moderate	Oropharyngeal
S088	151,848,979	151,649,490	7,167,855	Moderate	Oropharyngeal
S089	133,889,875	133,685,252	23,685,577	Moderate	Oropharyngeal
S090	151,331,786	148,740,987	4,069,049	Moderate	Oropharyngeal

S091	182,258,903	181,839,211	46,765,973	Moderate	Oropharyngeal
S092	143,623,098	143,489,785	5,380,671	Moderate	Oropharyngeal
S093	156,923,283	156,796,083	6,028,965	Moderate	Oropharyngeal
S094	138,468,686	137,802,865	19,625,834	Moderate	Oropharyngeal
S095	159,855,556	157,942,490	100,313,236	Moderate	Oropharyngeal
S096	138,618,776	137,594,385	9,121,683	Moderate	Oropharyngeal
S097	151,457,341	151,249,232	5,790,656	Sever	Oropharyngeal
S098	105,573,501	103,150,883	147,144,548	Mild	Oropharyngeal
S099	65,057,553	64,828,631	5,736,238	Moderate	Oropharyngeal
S100	97,071,080	96,952,005	9,779,622	Critical	Oropharyngeal
S101	103,528,766	103,409,031	5,922,519	Critical	Oropharyngeal
S102	87,114,568	87,031,075	19,125,501	Mild	Oropharyngeal
S103	195,129,296	194,856,066	21,397,669	Moderate	Oropharyngeal
S104	139,455,511	139,307,892	16,684,483	Sever	Oropharyngeal
S105	140,323,527	140,224,759	17,949,248	Sever	Oropharyngeal
S106	129,770,621	129,668,363	29,165,431	Moderate	Oropharyngeal
S107	115,878,258	115,794,388	19,459,834	Moderate	Oropharyngeal
S108	143,604,310	143,460,693	34,617,103	Moderate	Oropharyngeal
S109	151,626,971	151,558,179	59,852,603	Moderate	Oropharyngeal
S110	153,977,404	153,662,581	7,779,504	Moderate	Oropharyngeal
S111	110,493,665	110,260,730	29,232,254	Moderate	Oropharyngeal
S112	174,511,836	174,216,654	15,259,239	Moderate	Oropharyngeal
S113	146,554,956	146,288,296	12,476,645	Moderate	Oropharyngeal
S114	137,207,910	137,068,377	6,510,357	Moderate	Oropharyngeal
S115	154,433,804	154,304,293	5,314,930	Moderate	Oropharyngeal
S116	144,024,215	143,797,866	5,002,482	Moderate	Oropharyngeal

S117	78,796,076	78,729,959	4,297,755	Critical	Oropharyngeal
S118	68,383,040	68,299,675	9,757,494	Moderate	Oropharyngeal
S119	129,543,948	129,373,063	10,167,892	Moderate	Oropharyngeal
S120	122,191,658	122,042,271	4,728,509	Moderate	Oropharyngeal
S121	90,167,050	89,962,372	5,279,218	Moderate	Oropharyngeal
S122	115,753,472	115,708,297	39,216,130	Mild	Oropharyngeal
S123	146,656,800	146,489,865	9,281,342	Mild	Oropharyngeal
S124	154,109,860	154,000,085	32,817,180	Moderate	Oropharyngeal
S125	138,028,936	137,906,258	12,494,021	Moderate	Oropharyngeal
S126	170,827,379	170,715,839	33,803,781	Mild	Oropharyngeal
S127	115,676,961	115,614,836	17,761,720	Moderate	Oropharyngeal
S128	110,556,127	110,514,740	71,271,179	Normal	Oropharyngeal
S129	185,466,580	185,299,764	8,561,088	Moderate	Oropharyngeal
Total	17,203,683,422	17,140,618,365	2,321,491,400		
Average	124,664,373	124,207,379	16,822,401		

Table S4. Read numbers and detection rate of SARS-CoV-2

Sample	Tissue	No. of reads	SARS-CoV-2	SARS-CoV-2 (RPM)
S001	Anal	1927052	110	57.0820092
S002	Anal	5184032	1	0.192900044
S003	Anal	48676490	0	0
S004	Anal	2026638	0	0
S005	Anal	3173341	1	0.315125289
S006	Anal	4547109	2	0.439839907
S007	Anal	1064778	0	0
S008	Anal	2297999	8	3.481289592
S009	Anal	2344518	0	0
S010	Anal	2930306	26430	9019.535844
S011	Anal	2447166	0	0
S012	Anal	3432743	99	28.83991024
S013	Anal	1512037	0	0
S014	Anal	3242828	0	0
S015	Anal	3473881	0	0
S016	Anal	1434102	0	0
S017	Anal	2975284	0	0
S018	Anal	3171159	0	0
S019	Anal	12204988	0	0
S020	Anal	1844959	0	0
S021	Anal	4485058	11	2.452588127
S022	Anal	2144014	4	1.865659459

S023	Anal	2080991	0	0
S024	Anal	1962511	67	34.13993603
S025	Anal	9375143	47	5.013256864
			**Detection Rate**	**0.44**
S026	Nasopharyngeal	35292369	27	0.765037904
S027	Nasopharyngeal	3035671	0	0
S028	Nasopharyngeal	6069022	56	9.227186852
S029	Nasopharyngeal	2014500	0	0
S030	Nasopharyngeal	11138767	140	12.56871609
S031	Nasopharyngeal	3181923	0	0
S032	Nasopharyngeal	3168933	0	0
S033	Nasopharyngeal	2243257	25282	11270.22004
S034	Nasopharyngeal	4586693	10161	2215.321584
S035	Nasopharyngeal	2606379	254	97.45320999
S036	Nasopharyngeal	2822586	0	0
S037	Nasopharyngeal	1751866	2	1.141639829
S038	Nasopharyngeal	5273347	150	28.4449326
S039	Nasopharyngeal	3021486	1028	340.2299398
S040	Nasopharyngeal	7598814	0	0
S041	Nasopharyngeal	1837505	0	0
S042	Nasopharyngeal	2483739	0	0
S043	Nasopharyngeal	9719108	22109	2274.797234
S044	Nasopharyngeal	2840664	5909	2080.147458
S045	Nasopharyngeal	6313389	0	0
S046	Nasopharyngeal	3557699	0	0

S047	Nasopharyngeal	1852473	0	0
S048	Nasopharyngeal	2026751	0	0
S049	Nasopharyngeal	1368657	0	0
S050	Nasopharyngeal	2998566	0	0
S051	Nasopharyngeal	3109875	0	0
S052	Nasopharyngeal	1275522	0	0
S053	Nasopharyngeal	1763633	0	0
S054	Nasopharyngeal	6007327	0	0
			**Detection Rate**	**0.379310345**
S055	Oropharyngeal	50422695	136	2.697198157
S056	Oropharyngeal	108192564	0	0
S057	Oropharyngeal	1325456	0	0
S058	Oropharyngeal	5583395	0	0
S059	Oropharyngeal	74374356	8	0.107563957
S060	Oropharyngeal	35242507	1	0.028374826
S061	Oropharyngeal	24241621	42	1.732557406
S062	Oropharyngeal	30543239	0	0
S063	Oropharyngeal	19163456	0	0
S064	Oropharyngeal	2549111	0	0
S065	Oropharyngeal	5093695	0	0
S066	Oropharyngeal	55339910	1	0.018070141
S067	Oropharyngeal	11144603	0	0
S068	Oropharyngeal	14257026	0	0
S069	Oropharyngeal	6980287	2	0.28652117
S070	Oropharyngeal	70157254	0	0

S071	Oropharyngeal	26541429	0	0
S072	Oropharyngeal	18450868	0	0
S073	Oropharyngeal	23367034	0	0
S074	Oropharyngeal	43745252	0	0
S075	Oropharyngeal	15848734	0	0
S076	Oropharyngeal	82151391	1	0.012172648
S077	Oropharyngeal	30735595	1	0.032535567
S078	Oropharyngeal	5978961	2091	349.7263153
S079	Oropharyngeal	39871560	20	0.501610672
S080	Oropharyngeal	39073838	1	0.025592572
S081	Oropharyngeal	99651762	2	0.020069891
S082	Oropharyngeal	10997574	16349	1486.60059
S083	Oropharyngeal	35499398	2	0.056338984
S084	Oropharyngeal	9720385	0	0
S085	Oropharyngeal	1853280	121	65.28964862
S086	Oropharyngeal	11411253	23	2.015554295
S087	Oropharyngeal	3861439	1	0.258970814
S088	Oropharyngeal	6833166	11	1.609795518
S089	Oropharyngeal	15094582	33096	2192.574793
S090	Oropharyngeal	4047396	22	5.435593651
S091	Oropharyngeal	31342218	0	0
S092	Oropharyngeal	5375669	8	1.488186866
S093	Oropharyngeal	5674404	4	0.704919847
S094	Oropharyngeal	15654620	0	0
S095	Oropharyngeal	99409797	163083	1640.512353
S096	Oropharyngeal	6117766	704	115.0746858

S097	Oropharyngeal	4981351	2	0.401497505
S098	Oropharyngeal	145670247	1	0.00686482
S099	Oropharyngeal	5477365	0	0
S100	Oropharyngeal	9692440	12	1.238078337
S101	Oropharyngeal	5764825	4	0.693863214
S102	Oropharyngeal	18751535	1	0.053328967
S103	Oropharyngeal	21245904	0	0
S104	Oropharyngeal	16607220	18	1.083865933
S105	Oropharyngeal	17843960	2463	138.0298992
S106	Oropharyngeal	28932709	56	1.935525636
S107	Oropharyngeal	19275488	0	0
S108	Oropharyngeal	34357630	4	0.116422466
S109	Oropharyngeal	58974868	5	0.084781877
S110	Oropharyngeal	7691974	2	0.260011279
S111	Oropharyngeal	29003534	899	30.99622274
S112	Oropharyngeal	15149532	4	0.264034559
S113	Oropharyngeal	12386033	7619	615.1283466
S114	Oropharyngeal	6430721	1	0.155503559
S115	Oropharyngeal	4748964	0	0
S116	Oropharyngeal	4913254	0	0
S117	Oropharyngeal	4091414	7	1.710899948
S118	Oropharyngeal	9311714	100	10.73916145
S119	Oropharyngeal	10007260	0	0
S120	Oropharyngeal	4693148	0	0
S121	Oropharyngeal	4954954	0	0
S122	Oropharyngeal	38778945	0	0

S123	Oropharyngeal	9203717	0	0
S124	Oropharyngeal	31771058	0	0
S125	Oropharyngeal	12243707	2	0.163349221
S126	Oropharyngeal	33428733	1	0.029914385
S127	Oropharyngeal	17522596	3	0.171207508
S128	Oropharyngeal	69682597	0	0
S129	Oropharyngeal	8493935	5	0.588655317
			**Detection Rate**	**0.6**

Table S5. Threshold cycle (Ct) values for SARS-CoV-2

Sample	Group	No. of reads	SARS-CoV-2	SARS-CoV-2 (RPM)	Log10 of SARS-CoV-2 (RPM)	Ct value
S008	Anal	1512037	0	0	-3	37.31
S010	Nasopharyngeal	3035671	0	0	-3	38.49
S012	Nasopharyngeal	2014500	0	0	-3	38.56
S013	Nasopharyngeal	3181923	0	0	-3	37.54
S026	Nasopharyngeal	3168933	0	0	-3	37.02
S027	Nasopharyngeal	2483739	0	0	-3	35.81
S028	Oropharyngeal	14257026	0	0	-3	33.25
S029	Oropharyngeal	21245904	0	0	-3	39.12
S030	Oropharyngeal	9203717	0	0	-3	37.49
S031	Oropharyngeal	55339910	1	0.018070141	-1.743038449	34.23
S032	Oropharyngeal	58974868	5	0.084781877	-1.071696973	27.57
S033	Oropharyngeal	34357630	4	0.116422466	-0.933963207	30.31
S034	Oropharyngeal	6430721	1	0.155503559	-0.808259668	36.62
S035	Oropharyngeal	7691974	2	0.260011279	-0.585007812	34.17
S037	Oropharyngeal	6980287	2	0.28652117	-0.542843284	36.06
S038	Oropharyngeal	5764825	4	0.693863214	-0.158726137	32.33
S039	Nasopharyngeal	35292369	27	0.765037904	-0.116317047	27.3
S042	Oropharyngeal	16607220	18	1.083865933	0.034975566	27.13
S043	Nasopharyngeal	1751866	2	1.141639829	0.057529112	30.06
S044	Oropharyngeal	9692440	12	1.238078337	0.092748125	35.82
S066	Oropharyngeal	4091414	7	1.710899948	0.233224613	31.44
S068	Oropharyngeal	28932709	56	1.935525636	0.286798928	28.67

S069	Anal	2297999	8	3.481289592	0.541740152	33.14
S100	Nasopharyngeal	6069022	56	9.227186852	0.965069315	24.78
S101	Oropharyngeal	9311714	100	10.73916145	1.030970371	35.74
S103	Nasopharyngeal	11138767	140	12.56871609	1.099290916	26.39
S104	Nasopharyngeal	5273347	150	28.4449326	1.454004909	24.91
S105	Anal	3432743	99	28.83991024	1.459993904	31.73
S106	Oropharyngeal	29003534	899	30.99622274	1.491308773	33.79
S108	Nasopharyngeal	2606379	254	97.45320999	1.988796149	25.56
S109	Oropharyngeal	17843960	2463	138.0298992	2.139973171	24.72
S110	Nasopharyngeal	3021486	1028	340.2299398	2.531772528	25.22
S111	Oropharyngeal	12386033	7619	615.1283466	2.788965741	33.84
S113	Nasopharyngeal	2840664	5909	2080.147458	3.318094123	14.99
S114	Nasopharyngeal	4586693	10161	2215.321584	3.345436779	22.91
S117	Nasopharyngeal	9719108	22109	2274.797234	3.356942692	16.45
S118	Anal	2930306	26430	9019.535844	3.955184189	21.39
S123	Nasopharyngeal	2243257	25282	11270.22004	4.051932395	17.23

Table S6. Patient sex and symptoms

ID	Tissue	No. of reads	Sex	Symptom	Symptom2
S001	Anal	1434102	female	Mild	Moderate
S002	Anal	2026638	female	Mild	Moderate
S003	Anal	3171159	female	Mild	Moderate
S004	Anal	1844959	male	Mild	Moderate
S005	Anal	3432743	male	Mild	Moderate
S006	Anal	1064778	female	Moderate	Moderate
S007	Anal	1962511	female	Moderate	Moderate
S008	Anal	2297999	female	Moderate	Moderate
S009	Anal	5184032	female	Moderate	Moderate
S010	Anal	9375143	female	Moderate	Moderate
S012	Anal	12204988	female	Moderate	Moderate
S013	Anal	48676490	female	Moderate	Moderate
S014	Anal	1927052	male	Moderate	Moderate
S015	Anal	2144014	male	Moderate	Moderate
S016	Anal	2930306	male	Moderate	Moderate
S017	Anal	2975284	male	Moderate	Moderate
S018	Anal	3473881	male	Moderate	Moderate
S019	Anal	4485058	male	Moderate	Moderate
S020	Anal	4547109	male	Moderate	Moderate
S021	Anal	1512037	female	Sever	Sever
S022	Anal	2344518	male	Sever	Sever
S024	Anal	3242828	male	Sever	Sever

S025	Anal	3173341	male	Critical	Sever
S026	Nasopharyngeal	1763633	female	Mild	Moderate
S027	Nasopharyngeal	2822586	male	Mild	Moderate
S028	Nasopharyngeal	6007327	male	Mild	Moderate
S029	Nasopharyngeal	6069022	male	Mild	Moderate
S030	Nasopharyngeal	9719108	NA	Mild	Moderate
S031	Nasopharyngeal	1275522	female	Moderate	Moderate
S032	Nasopharyngeal	2014500	female	Moderate	Moderate
S033	Nasopharyngeal	2243257	female	Moderate	Moderate
S034	Nasopharyngeal	2606379	female	Moderate	Moderate
S035	Nasopharyngeal	2998566	female	Moderate	Moderate
S036	Nasopharyngeal	3109875	female	Moderate	Moderate
S037	Nasopharyngeal	3168933	female	Moderate	Moderate
S038	Nasopharyngeal	4586693	female	Moderate	Moderate
S039	Nasopharyngeal	6313389	female	Moderate	Moderate
S040	Nasopharyngeal	11138767	female	Moderate	Moderate
S041	Nasopharyngeal	1368657	male	Moderate	Moderate
S042	Nasopharyngeal	1852473	male	Moderate	Moderate
S043	Nasopharyngeal	3021486	male	Moderate	Moderate
S044	Nasopharyngeal	3035671	male	Moderate	Moderate
S045	Nasopharyngeal	3557699	male	Moderate	Moderate
S046	Nasopharyngeal	5273347	male	Moderate	Moderate
S047	Nasopharyngeal	7598814	male	Moderate	Moderate
S048	Nasopharyngeal	35292369	male	Moderate	Moderate
S049	Nasopharyngeal	2840664	NA	Moderate	Moderate
S050	Nasopharyngeal	3181923	female	Sever	Sever

S051	Nasopharyngeal	1751866	male	Sever	Sever
S052	Nasopharyngeal	1837505	male	Sever	Sever
S053	Nasopharyngeal	2483739	male	Sever	Sever
S054	Nasopharyngeal	2026751	male	Critical	Sever
S055	Oropharyngeal	5583395	female	Mild	Moderate
S056	Oropharyngeal	18450868	female	Mild	Moderate
S057	Oropharyngeal	18751535	female	Mild	Moderate
S058	Oropharyngeal	38778945	female	Mild	Moderate
S059	Oropharyngeal	43745252	female	Mild	Moderate
S060	Oropharyngeal	145670247	female	Mild	Moderate
S061	Oropharyngeal	5978961	male	Mild	Moderate
S062	Oropharyngeal	9203717	male	Mild	Moderate
S063	Oropharyngeal	14257026	male	Mild	Moderate
S065	Oropharyngeal	33428733	male	Mild	Moderate
S066	Oropharyngeal	1325456	female	Moderate	Moderate
S068	Oropharyngeal	3861439	female	Moderate	Moderate
S069	Oropharyngeal	4047396	female	Moderate	Moderate
S070	Oropharyngeal	4693148	female	Moderate	Moderate
S071	Oropharyngeal	4954954	female	Moderate	Moderate
S072	Oropharyngeal	5477365	female	Moderate	Moderate
S073	Oropharyngeal	5674404	female	Moderate	Moderate
S074	Oropharyngeal	6117766	female	Moderate	Moderate
S075	Oropharyngeal	6430721	female	Moderate	Moderate
S076	Oropharyngeal	7691974	female	Moderate	Moderate
S077	Oropharyngeal	8493935	female	Moderate	Moderate
S078	Oropharyngeal	10007260	female	Moderate	Moderate

S079	Oropharyngeal	10997574	female	Moderate	Moderate
S080	Oropharyngeal	12243707	female	Moderate	Moderate
S081	Oropharyngeal	12386033	female	Moderate	Moderate
S082	Oropharyngeal	15094582	female	Moderate	Moderate
S083	Oropharyngeal	15848734	female	Moderate	Moderate
S084	Oropharyngeal	19275488	female	Moderate	Moderate
S085	Oropharyngeal	24241621	female	Moderate	Moderate
S086	Oropharyngeal	28932709	female	Moderate	Moderate
S087	Oropharyngeal	29003534	female	Moderate	Moderate
S088	Oropharyngeal	30543239	female	Moderate	Moderate
S089	Oropharyngeal	30735595	female	Moderate	Moderate
S090	Oropharyngeal	31342218	female	Moderate	Moderate
S091	Oropharyngeal	34357630	female	Moderate	Moderate
S092	Oropharyngeal	39871560	female	Moderate	Moderate
S093	Oropharyngeal	108192564	female	Moderate	Moderate
S094	Oropharyngeal	1853280	male	Moderate	Moderate
S095	Oropharyngeal	4748964	male	Moderate	Moderate
S096	Oropharyngeal	4913254	male	Moderate	Moderate
S097	Oropharyngeal	5375669	male	Moderate	Moderate
S098	Oropharyngeal	6833166	male	Moderate	Moderate
S099	Oropharyngeal	9311714	male	Moderate	Moderate
S100	Oropharyngeal	9720385	male	Moderate	Moderate
S101	Oropharyngeal	11411253	male	Moderate	Moderate
S102	Oropharyngeal	15149532	male	Moderate	Moderate
S103	Oropharyngeal	15654620	male	Moderate	Moderate
S104	Oropharyngeal	17522596	male	Moderate	Moderate

S105	Oropharyngeal	21245904	male	Moderate	Moderate
S106	Oropharyngeal	23367034	male	Moderate	Moderate
S107	Oropharyngeal	26541429	male	Moderate	Moderate
S108	Oropharyngeal	35242507	male	Moderate	Moderate
S109	Oropharyngeal	50422695	male	Moderate	Moderate
S110	Oropharyngeal	55339910	male	Moderate	Moderate
S111	Oropharyngeal	58974868	male	Moderate	Moderate
S112	Oropharyngeal	99409797	male	Moderate	Moderate
S113	Oropharyngeal	99651762	male	Moderate	Moderate
S114	Oropharyngeal	31771058	NA	Moderate	Moderate
S115	Oropharyngeal	6980287	female	Sever	Sever
S116	Oropharyngeal	17843960	female	Sever	Sever
S117	Oropharyngeal	19163456	female	Sever	Sever
S118	Oropharyngeal	82151391	female	Sever	Sever
S119	Oropharyngeal	4981351	male	Sever	Sever
S120	Oropharyngeal	5093695	male	Sever	Sever
S121	Oropharyngeal	16607220	male	Sever	Sever
S122	Oropharyngeal	35499398	male	Sever	Sever
S123	Oropharyngeal	39073838	male	Sever	Sever
S124	Oropharyngeal	70157254	male	Sever	Sever
S125	Oropharyngeal	4091414	male	Critical	Sever
S126	Oropharyngeal	5764825	male	Critical	Sever
S127	Oropharyngeal	9692440	male	Critical	Sever
S129	Oropharyngeal	74374356	male	Critical	Sever

Table S7.1. Species-level α-diversity declined with increasing disease severity in Faeces

ID	Tissue	No. of reads	Sex	Symptom1	Symptom2
S001	Anal	1434102	female	Mild	Moderate
S002	Anal	2026638	female	Mild	Moderate
S003	Anal	3171159	female	Mild	Moderate
S004	Anal	1844959	male	Mild	Moderate
S005	Anal	3432743	male	Mild	Moderate
S006	Anal	1064778	female	Moderate	Moderate
S007	Anal	1962511	female	Moderate	Moderate
S008	Anal	2297999	female	Moderate	Moderate
S009	Anal	5184032	female	Moderate	Moderate
S010	Anal	9375143	female	Moderate	Moderate
S012	Anal	12204988	female	Moderate	Moderate
S013	Anal	48676490	female	Moderate	Moderate
S014	Anal	1927052	male	Moderate	Moderate
S015	Anal	2144014	male	Moderate	Moderate
S016	Anal	2930306	male	Moderate	Moderate
S017	Anal	2975284	male	Moderate	Moderate
S018	Anal	3473881	male	Moderate	Moderate
S019	Anal	4485058	male	Moderate	Moderate
S020	Anal	4547109	male	Moderate	Moderate
S021	Anal	1512037	female	Sever	Sever
S022	Anal	2344518	male	Sever	Sever
S024	Anal	3242828	male	Sever	Sever
S025	Anal	3173341	male	Critical	Sever

Table S7.2. Species-level α-diversity declined with increasing disease severity in NPs

ID	Tissue	No. of reads	Sex	Symptom1	Symptom2
S026	Nasopharyngeal	1763633	female	Mild	Moderate
S027	Nasopharyngeal	2822586	male	Mild	Moderate
S028	Nasopharyngeal	6007327	male	Mild	Moderate
S029	Nasopharyngeal	6069022	male	Mild	Moderate
S030	Nasopharyngeal	9719108	NA	Mild	Moderate
S031	Nasopharyngeal	1275522	female	Moderate	Moderate
S032	Nasopharyngeal	2014500	female	Moderate	Moderate
S033	Nasopharyngeal	2243257	female	Moderate	Moderate
S034	Nasopharyngeal	2606379	female	Moderate	Moderate
S035	Nasopharyngeal	2998566	female	Moderate	Moderate
S036	Nasopharyngeal	3109875	female	Moderate	Moderate
S037	Nasopharyngeal	3168933	female	Moderate	Moderate
S038	Nasopharyngeal	4586693	female	Moderate	Moderate
S039	Nasopharyngeal	6313389	female	Moderate	Moderate
S040	Nasopharyngeal	11138767	female	Moderate	Moderate
S041	Nasopharyngeal	1368657	male	Moderate	Moderate
S042	Nasopharyngeal	1852473	male	Moderate	Moderate
S043	Nasopharyngeal	3021486	male	Moderate	Moderate
S044	Nasopharyngeal	3035671	male	Moderate	Moderate
S045	Nasopharyngeal	3557699	male	Moderate	Moderate
S046	Nasopharyngeal	5273347	male	Moderate	Moderate
S047	Nasopharyngeal	7598814	male	Moderate	Moderate

S048	Nasopharyngeal	35292369	male	Moderate	Moderate
S049	Nasopharyngeal	2840664	NA	Moderate	Moderate
S050	Nasopharyngeal	3181923	female	Sever	Sever
S051	Nasopharyngeal	1751866	male	Sever	Sever
S052	Nasopharyngeal	1837505	male	Sever	Sever
S053	Nasopharyngeal	2483739	male	Sever	Sever
S054	Nasopharyngeal	2026751	male	Critical	Sever

Table S7.3. Species-level α-diversity declined with increasing disease severity in OPs

ID	Tissue	No. of reads	Sex	Symptom1	Symptom2
S055	Oropharyngeal	5583395	female	Mild	Moderate
S056	Oropharyngeal	18450868	female	Mild	Moderate
S057	Oropharyngeal	18751535	female	Mild	Moderate
S058	Oropharyngeal	38778945	female	Mild	Moderate
S059	Oropharyngeal	43745252	female	Mild	Moderate
S060	Oropharyngeal	145670247	female	Mild	Moderate
S061	Oropharyngeal	5978961	male	Mild	Moderate
S062	Oropharyngeal	9203717	male	Mild	Moderate
S063	Oropharyngeal	14257026	male	Mild	Moderate
S065	Oropharyngeal	33428733	male	Mild	Moderate
S066	Oropharyngeal	1325456	female	Moderate	Moderate
S068	Oropharyngeal	3861439	female	Moderate	Moderate
S069	Oropharyngeal	4047396	female	Moderate	Moderate
S070	Oropharyngeal	4693148	female	Moderate	Moderate

S071	Oropharyngeal	4954954	female	Moderate	Moderate
S072	Oropharyngeal	5477365	female	Moderate	Moderate
S073	Oropharyngeal	5674404	female	Moderate	Moderate
S074	Oropharyngeal	6117766	female	Moderate	Moderate
S075	Oropharyngeal	6430721	female	Moderate	Moderate
S076	Oropharyngeal	7691974	female	Moderate	Moderate
S077	Oropharyngeal	8493935	female	Moderate	Moderate
S078	Oropharyngeal	10007260	female	Moderate	Moderate
S079	Oropharyngeal	10997574	female	Moderate	Moderate
S080	Oropharyngeal	12243707	female	Moderate	Moderate
S081	Oropharyngeal	12386033	female	Moderate	Moderate
S082	Oropharyngeal	15094582	female	Moderate	Moderate
S083	Oropharyngeal	15848734	female	Moderate	Moderate
S084	Oropharyngeal	19275488	female	Moderate	Moderate
S085	Oropharyngeal	24241621	female	Moderate	Moderate
S086	Oropharyngeal	28932709	female	Moderate	Moderate
S087	Oropharyngeal	29003534	female	Moderate	Moderate
S088	Oropharyngeal	30543239	female	Moderate	Moderate
S089	Oropharyngeal	30735595	female	Moderate	Moderate
S090	Oropharyngeal	31342218	female	Moderate	Moderate
S091	Oropharyngeal	34357630	female	Moderate	Moderate
S092	Oropharyngeal	39871560	female	Moderate	Moderate
S093	Oropharyngeal	108192564	female	Moderate	Moderate
S094	Oropharyngeal	1853280	male	Moderate	Moderate
S095	Oropharyngeal	4748964	male	Moderate	Moderate
S096	Oropharyngeal	4913254	male	Moderate	Moderate

S097	Oropharyngeal	5375669	male	Moderate	Moderate
S098	Oropharyngeal	6833166	male	Moderate	Moderate
S099	Oropharyngeal	9311714	male	Moderate	Moderate
S100	Oropharyngeal	9720385	male	Moderate	Moderate
S101	Oropharyngeal	11411253	male	Moderate	Moderate
S102	Oropharyngeal	15149532	male	Moderate	Moderate
S103	Oropharyngeal	15654620	male	Moderate	Moderate
S104	Oropharyngeal	17522596	male	Moderate	Moderate
S105	Oropharyngeal	21245904	male	Moderate	Moderate
S106	Oropharyngeal	23367034	male	Moderate	Moderate
S107	Oropharyngeal	26541429	male	Moderate	Moderate
S108	Oropharyngeal	35242507	male	Moderate	Moderate
S109	Oropharyngeal	50422695	male	Moderate	Moderate
S110	Oropharyngeal	55339910	male	Moderate	Moderate
S111	Oropharyngeal	58974868	male	Moderate	Moderate
S112	Oropharyngeal	99409797	male	Moderate	Moderate
S113	Oropharyngeal	99651762	male	Moderate	Moderate
S114	Oropharyngeal	31771058	NA	Moderate	Moderate
S115	Oropharyngeal	6980287	female	Sever	Sever
S116	Oropharyngeal	17843960	female	Sever	Sever
S117	Oropharyngeal	19163456	female	Sever	Sever
S118	Oropharyngeal	82151391	female	Sever	Sever
S119	Oropharyngeal	4981351	male	Sever	Sever
S120	Oropharyngeal	5093695	male	Sever	Sever
S121	Oropharyngeal	16607220	male	Sever	Sever
S122	Oropharyngeal	35499398	male	Sever	Sever

S123	Oropharyngeal	39073838	male	Sever	Sever
S124	Oropharyngeal	70157254	male	Sever	Sever
S125	Oropharyngeal	4091414	male	Critical	Sever
S126	Oropharyngeal	5764825	male	Critical	Sever
S127	Oropharyngeal	9692440	male	Critical	Sever
S129	Oropharyngeal	74374356	male	Critical	Sever

Table S8.1. Human genes whose expression correlated negatively with COVID-19 severity in Faeces used the spearman analysis

KO	Pathway	Subtype	Type	No. of genes	*p* value	Gene ID
190	Oxidative phosphorylation	Energy metabolism	Metabolism	2	2.2E-16	XM_011531179.1,NM_001183.6,
4144	Endocytosis	Transport and catabolism	Cellular Processes	11	0.000002512	XM_005265384.4,XM_017007026.1,XM_017007025.2,XM_011534001.2,NM_022340.4,NM_001302378.2,XM_017007024.2,XM_017007027.2,XM_017007028.2,XM_005265385.4,XM_017007023.1,
3410	Base excision repair	Replication and repair	Genetic Information Processing	4	0.0001644	NM_001015052.3,NM_002434.4,NM_001015054.2, XM_024450282.1,
3020	RNA polymerase	Transcription	Genetic Information Processing	3	0.0003053	XM_005277431.4,NM_001330685.1,NM_032305.3,
4623	Cytosolic DNA-sensing pathway	Immune system	Organismal Systems	3	0.001452	XM_005277431.4,NM_001330685.1,NM_032305.3,
4740	Olfactory transduction	Sensory system	Organismal Systems	4	0.006889	NM_001005286.1,NM_024082.3,XM_011531197.2, NM_001372163.1,
5120	Epithelial cell signaling in Helicobacter pylori infection	Infectious diseases: Bacterial	Human Diseases	2	0.02802	XM_011531179.1,NM_001183.6,
5110	Vibrio cholerae infection	Infectious diseases:	Human Diseases	2	0.03658	XM_011531179.1,NM_001183.6,

5323	Rheumatoid arthritis	Bacterial Immune diseases	Human Diseases	2	0.04062	XM_011531179.1,NM_001183.6,
4024	cAMP signaling pathway	Signal transduction	Environmental Information Processing	3	0.09101	NM_024082.3,XM_011531197.2,NM_001372163.1,
3015	mRNA surveillance pathway	Translation	Genetic Information Processing	2	0.1114	NM_032345.3,NM_001143853.1,
5161	Hepatitis B	Infectious diseases: Viral	Human Diseases	2	0.1119	XM_011531179.1,NM_001183.6,
1100	Metabolic pathways	Global and overview maps	Metabolism	2	0.1288	XM_011531179.1,NM_001183.6,
4218	Cellular senescence	Cell growth and death	Cellular Processes	2	0.1382	NM_001018081.1,NM_175885.4,
4145	Phagosome	Transport and catabolism	Cellular Processes	2	0.1401	XM_011531179.1,NM_001183.6,
5216	Thyroid cancer	Cancers: Specific types	Human Diseases	1	0.1516	NM_175885.4,
5152	Tuberculosis	Infectious diseases: Bacterial	Human Diseases	2	0.1613	XM_011531179.1,NM_001183.6,
4142	Lysosome	Transport and catabolism	Cellular Processes	2	0.1627	XM_011531179.1,NM_001183.6,
3013	RNA transport	Translation	Genetic Information	2	0.1888	NM_032345.3,NM_001143853.1,

			Processing			
5223	Non-small cell lung cancer	Cancers: Specific types	Human Diseases	1	0.2485	NM_175885.4,
5218	Melanoma	Cancers: Specific types	Human Diseases	1	0.2563	NM_175885.4,
5214	Glioma	Cancers: Specific types	Human Diseases	1	0.2647	NM_175885.4,
5212	Pancreatic cancer	Cancers: Specific types	Human Diseases	1	0.2782	NM_175885.4,
5220	Chronic myeloid leukemia	Cancers: Specific types	Human Diseases	1	0.3084	NM_175885.4,
4612	Antigen processing and presentation	Immune system	Organismal Systems	1	0.3123	NM_001018081.1,
5213	Endometrial cancer	Cancers: Specific types	Human Diseases	1	0.3129	NM_175885.4,
4650	Natural killer cell mediated cytotoxicity	Immune system	Organismal Systems	1	0.3129	NM_001018081.1,
4115	p53 signaling pathway	Cell growth and death	Cellular Processes	1	0.3179	NM_175885.4,
5210	Colorectal cancer	Cancers: Specific types	Human Diseases	1	0.3233	NM_175885.4,
4610	Complement and coagulation cascades	Immune system	Organismal Systems	1	0.3277	NM_001372165.1,
5217	Basal cell carcinoma	Cancers: Specific types	Human Diseases	1	0.3347	NM_175885.4,

5222	Small cell lung cancer	Cancers: Specific types	Human Diseases	1	0.3558	NM_175885.4,
4068	FoxO signaling pathway	Signal transduction	Environmental Information Processing	1	0.3677	NM_175885.4,
4110	Cell cycle	Cell growth and death	Cellular Processes	1	0.449	NM_175885.4,
4210	Apoptosis	Cell growth and death	Cellular Processes	1	0.4565	NM_175885.4,
5169	Epstein-Barr virus infection	Infectious diseases: Viral	Human Diseases	1	0.5202	NM_175885.4,
5200	Pathways in cancer	Cancers: Overview	Human Diseases	1	0.5252	NM_175885.4,
5224	Breast cancer	Cancers: Specific types	Human Diseases	1	0.5695	NM_175885.4,
5225	Hepatocellular carcinoma	Cancers: Specific types	Human Diseases	1	0.5773	NM_175885.4,
5226	Gastric cancer	Cancers: Specific types	Human Diseases	1	0.5841	NM_175885.4,
5165	Human papillomavirus infection	Infectious diseases: Viral	Human Diseases	2	1	XM_011531179.1,NM_001183.6,
4010	MAPK signaling pathway	Signal transduction	Environmental Information Processing	1	1	NM_175885.4,
4020	Calcium signaling	Signal	Environmental	1	1	NM_005314.3,

	pathway	transduction	Information Processing			
4080	Neuroactive ligand-receptor interaction	Signaling molecules and interaction	Environmental Information Processing	1	1	NM_005314.3,
5202	Transcriptional misregulation in cancer	Cancers: Overview	Human Diseases	1	1	NM_175885.4,

Table S8.2. Human genes whose expression correlated positively with COVID-19 severity in Faeces used the spearman analysis

KO	Pathway	Subtype	Type	No. of genes	*p* value	Gene ID
260	Glycine, serine and threonine metabolism	Amino acid metabolism	Metabolism	1	3.464E-13	NM_000290.4,
10	Glycolysis / Gluconeogenesis	Carbohydrate metabolism	Metabolism	1	3.464E-13	NM_000290.4,
680	Methane metabolism	Energy metabolism	Metabolism	1	3.464E-13	NM_000290.4,
500	Starch and sucrose metabolism	Carbohydrate metabolism	Metabolism	2	4.335E-12	NM_001008218.1,XM_011541262.1,
5150	Staphylococcus aureus infection	Infectious diseases: Bacterial	Human Diseases	3	0.000334	NM_001242823.2,NM_001302265.1,NM_001042500.1,
4973	Carbohydrate digestion and absorption	Digestive system	Organismal Systems	2	0.002504	NM_001008218.1,XM_011541262.1,
4927	Cortisol synthesis and secretion	Endocrine system	Organismal Systems	3	0.002546	NM_001291911.1,NM_000529.2,XM_017025781.1,
4925	Aldosterone synthesis and secretion	Endocrine system	Organismal Systems	3	0.005108	NM_001291911.1,NM_000529.2,XM_017025781.1,
4934	Cushing syndrome	Endocrine and metabolic diseases	Human Diseases	3	0.01901	NM_001291911.1,NM_000529.2,XM_017025781.1,

4024	cAMP signaling pathway	Signal transduction	Environmental Information Processing	3	0.02631	NM_001291911.1,NM_000529.2,XM_017025781.1,
4080	Neuroactive ligand-receptor interaction	Signaling molecules and interaction	Environmental Information Processing	3	0.0465	NM_001291911.1,NM_000529.2,XM_017025781.1,
4740	Olfactory transduction	Sensory system	Organismal Systems	2	0.07091	NM_001005160.2,NM_001005241.3,
4621	NOD-like receptor signaling pathway	Immune system	Organismal Systems	2	0.07105	NM_001302265.1,NM_001042500.1,
5230	Central carbon metabolism in cancer	Cancers: Overview	Human Diseases	1	0.1406	NM_000290.4,
5322	Systemic lupus erythematosus	Immune diseases	Human Diseases	1	0.1641	NM_001242823.2,
5133	Pertussis	Infectious diseases: Bacterial	Human Diseases	1	0.1673	NM_001242823.2,
1230	Biosynthesis of amino acids	Global and overview maps	Metabolism	1	0.1737	NM_000290.4,
3010	Ribosome	Translation	Genetic Information Processing	1	0.2005	NM_021029.6,
4610	Complement and coagulation cascades	Immune system	Organismal Systems	1	0.2096	NM_001242823.2,

4972	Pancreatic secretion	Digestive system	Organismal Systems	1	0.2312	NM_001869.3,
4914	Progesterone-mediated oocyte maturation	Endocrine system	Organismal Systems	1	0.2393	NM_001382525.1,
4974	Protein digestion and absorption	Digestive system	Organismal Systems	1	0.2454	NM_001869.3,
4922	Glucagon signaling pathway	Endocrine system	Organismal Systems	1	0.2643	NM_000290.4,
1200	Carbon metabolism	Global and overview maps	Metabolism	1	0.2664	NM_000290.4,
4114	Oocyte meiosis	Cell growth and death	Cellular Processes	1	0.3144	NM_001382525.1,
1120	Microbial metabolism in diverse environments	Global and overview maps	Metabolism	1	0.3913	NM_000290.4,
1130	Biosynthesis of antibiotics	Global and overview maps	Metabolism	1	0.4348	NM_000290.4,
1100	Metabolic pathways	Global and overview maps	Metabolism	3	1	NM_000290.4,NM_001008218.1,XM_011541262.1,
1110	Biosynthesis of secondary metabolites	Global and overview maps	Metabolism	1	1	NM_000290.4,

Table S8.3. Human genes whose expression correlated negatively with COVID-19 severity in NPs used the spearman analysis

KO	Pathway	Subtype	Type	No. of genes	*p* value	Gene ID
521	Streptomycin biosynthesis	Biosynthesis of other secondary metabolites	Metabolism	3	2.2E-16	NM_001172819.1,NM_002633.3,NM_001172818.1,
10	Glycolysis / Gluconeogenesis	Carbohydrate metabolism	Metabolism	3	2.2E-16	NM_001172819.1,NM_002633.3,NM_001172818.1,
30	Pentose phosphate pathway	Carbohydrate metabolism	Metabolism	3	2.2E-16	NM_001172819.1,NM_002633.3,NM_001172818.1,
52	Galactose metabolism	Carbohydrate metabolism	Metabolism	3	2.2E-16	NM_001172819.1,NM_002633.3,NM_001172818.1,
500	Starch and sucrose metabolism	Carbohydrate metabolism	Metabolism	3	2.2E-16	NM_001172819.1,NM_002633.3,NM_001172818.1,
520	Amino sugar and nucleotide sugar metabolism	Carbohydrate metabolism	Metabolism	3	2.2E-16	NM_001172819.1,NM_002633.3,NM_001172818.1,
760	Nicotinate and nicotinamide metabolism	Metabolism of cofactors and vitamins	Metabolism	3	2.2E-16	NM_001024656.3,XM_024451585.1,NM_001114598.2,
230	Purine metabolism	Nucleotide metabolism	Metabolism	3	2.2E-16	NM_001172819.1,NM_002633.3,NM_001172818.1,
140	Steroid hormone biosynthesis	Lipid metabolism	Metabolism	2	2.2E-16	NM_000781.3,NM_001099773.2,
220	Arginine	Amino acid	Metabolism	1	2.2E-16	NM_007088.3,

	biosynthesis	metabolism				
250	Alanine, aspartate and glutamate metabolism	Amino acid metabolism	Metabolism	1	2.2E-16	NM_007088.3,
270	Cysteine and methionine metabolism	Amino acid metabolism	Metabolism	1	2.2E-16	NM_007088.3,
330	Arginine and proline metabolism	Amino acid metabolism	Metabolism	1	2.2E-16	NM_007088.3,
350	Tyrosine metabolism	Amino acid metabolism	Metabolism	1	2.2E-16	NM_007088.3,
360	Phenylalanine metabolism	Amino acid metabolism	Metabolism	1	2.2E-16	NM_007088.3,
400	Phenylalanine, tyrosine and tryptophan biosynthesis	Amino acid metabolism	Metabolism	1	2.2E-16	NM_007088.3,
950	Isoquinoline alkaloid biosynthesis	Biosynthesis of other secondary metabolites	Metabolism	1	2.2E-16	NM_007088.3,
960	Tropane, piperidine and pyridine alkaloid biosynthesis	Biosynthesis of other secondary metabolites	Metabolism	1	2.2E-16	NM_007088.3,
710	Carbon fixation in	Energy	Metabolism	1	2.2E-16	NM_007088.3,

	photosynthetic organisms	metabolism				
511	Other glycan degradation	Glycan biosynthesis and metabolism	Metabolism	1	2.2E-16	NM_005383.2,
600	Sphingolipid metabolism	Lipid metabolism	Metabolism	1	2.2E-16	NM_005383.2,
983	Drug metabolism - other enzymes	Xenobiotics biodegradation and metabolism	Metabolism	1	2.2E-16	XM_017030236.2,
1100	Metabolic pathways	Global and overview maps	Metabolism	10	1.879E-10	NM_001024656.3,NM_000781.3,NM_001172819.1,XM_024451585.1,NM_002633.3,XM_017030236.2,NM_001099773.2,NM_001114598.2,NM_007088.3,NM_001172818.1,
5144	Malaria	Infectious diseases: Parasitic	Human Diseases	11	2.228E-07	XM_011524889.2,XM_005276880.1,XM_017024741.1,XM_005276882.1,XM_017024738.1,XM_017024739.1,NM_000442.5,XM_005276883.2,XM_005276881.1,XM_017024740.1,XM_011524890.1,
4392	Hippo signaling pathway - multiple species	Signal transduction	Environmental Information Processing	10	0.000001321	NM_007182.5,NM_201443.3,NM_201441.3,NM_170712.3,NM_170713.3,NM_003213.4,NM_001206957.1,XM_011533316.2,NM_170714.2,XM_024453328.1,
4621	NOD-like receptor signaling pathway	Immune system	Organismal Systems	19	0.000007827	NM_001321135.2,XM_011522167.2,XM_011522165.2,XM_011522166.2,XM_005273356.2,XM_01700

						2868.1,XM_011522164.1,NM_001321137.1,XM_011522163.2,NM_001926.4,XM_011522168.3,NM_001193322.2,NM_014002.4,NM_001193321.2,NM_001321136.2,NM_003978.5,XM_006720737.3,XM_011522169.2,NM_001162371.3,
4670	Leukocyte transendothelial migration	Immune system	Organismal Systems	15	0.00004041	NM_005428.4,XM_011524889.2,XM_005276880.1,XM_017024741.1,XM_005276882.1,NM_001258207.2,XM_017024738.1,XM_005259642.1,NM_001258206.2,XM_017024739.1,NM_000442.5,XM_005276883.2,XM_005276881.1,XM_017024740.1,XM_011524890.1,
5130	Pathogenic Escherichia coli infection	Infectious diseases: Bacterial	Human Diseases	8	0.00009308	NM_001289130.1,NM_006086.4,NM_001289131.1,NM_001289129.1,NM_001289123.1,NM_001289127.1,NM_001197181.2,NM_006087.4,
4664	Fc epsilon RI signaling pathway	Immune system	Organismal Systems	8	0.0001591	NM_005428.4,NM_014387.4,NM_001014988.2,NM_001014989.2,NM_001258207.2,XM_005259642.1,NM_001258206.2,NM_001014987.2,
5120	Epithelial cell signaling in Helicobacter pylori infection	Infectious diseases: Bacterial	Human Diseases	9	0.0002173	NM_001354988.2,XM_005246530.3,XM_017003992.1,NM_001557.4,XM_017003990.1,XM_017003991.1,NM_004383.2,NM_001168298.2,NM_001127190.2,
5219	Bladder cancer	Cancers: Specific types	Human Diseases	7	0.0003792	NM_007182.5,NM_170712.3,NM_170713.3,NM_001206957.1,XM_011533316.2,NM_170714.2,XM_024453328.1,
4151	PI3K-Akt signaling pathway	Signal transduction	Environmental Information	2	0.0007216	XM_005244891.5,XM_011509163.3,

			Processing			
5202	Transcriptional misregulation in cancer	Cancers: Overview	Human Diseases	1	0.001453	NM_003493.3,
5223	Non-small cell lung cancer	Cancers: Specific types	Human Diseases	8	0.001999	NM_007182.5,NM_170712.3,NM_170713.3,NM_001206957.1,XM_011533316.2,NM_170714.2,NM_032484.5,XM_024453328.1,
1110	Biosynthesis of secondary metabolites	Global and overview maps	Metabolism	4	0.002729	NM_001172819.1,NM_002633.3,NM_007088.3,NM_001172818.1,
4140	Autophagy - animal	Transport and catabolism	Cellular Processes	1	0.002956	NM_001017921.4,
4062	Chemokine signaling pathway	Immune system	Organismal Systems	14	0.003184	NM_005428.4,XM_005246530.3,XM_017003992.1,NM_001557.4,NM_001258207.2,XM_005259642.1,XM_017003990.1,NM_001258206.2,XM_017003991.1,NM_001168298.2,NM_032484.5,NM_001320037.1,NM_001320038.2,XM_017015106.2,
5165	Human papillomavirus infection	Infectious diseases: Viral	Human Diseases	5	0.003999	XM_005273356.2,XM_017002868.1,NM_001193322.2,NM_014002.4,NM_001193321.2,
4145	Phagosome	Transport and catabolism	Cellular Processes	12	0.0059	NM_001289130.1,NM_006086.4,NM_001289131.1,XM_011512082.2,NM_001289129.1,NM_001289123.1,NM_006770.3,XM_017005171.2,XM_011512083.3,NM_001289127.1,NM_001197181.2,NM_006087.4,
4660	T cell receptor	Immune	Organismal	8	0.006042	NM_005428.4,NM_014387.4,NM_001014988.2,NM

	signaling pathway	system	Systems			_001014989.2,NM_001258207.2,XM_005259642.1,NM_001258206.2,NM_001014987.2,
4360	Axon guidance	Development	Organismal Systems	1	0.006076	NM_173561.3,
4714	Thermogenesis	Environmental adaptation	Organismal Systems	1	0.006158	NM_001006665.2,
4075	Plant hormone signal transduction	Signal transduction	Environmental Information Processing	1	0.006262	NM_001142623.2,
5203	Viral carcinogenesis	Cancers: Overview	Human Diseases	1	0.006379	NM_032484.5,
4666	Fc gamma R-mediated phagocytosis	Immune system	Organismal Systems	8	0.006636	NM_005428.4,NM_014387.4,NM_001014988.2,NM_001014989.2,NM_001258207.2,XM_005259642.1,NM_001258206.2,NM_001014987.2,
5418	Fluid shear stress and atherosclerosis	Cardiovascular diseases	Human Diseases	11	0.008298	XM_011524889.2,XM_005276880.1,XM_017024741.1,XM_005276882.1,XM_017024738.1,XM_017024739.1,NM_000442.5,XM_005276883.2,XM_005276881.1,XM_017024740.1,XM_011524890.1,
4650	Natural killer cell mediated cytotoxicity	Immune system	Organismal Systems	8	0.009928	NM_005428.4,NM_014387.4,NM_001014988.2,NM_001014989.2,NM_001258207.2,XM_005259642.1,NM_001258206.2,NM_001014987.2,
5166	Human T-cell leukemia virus 1 infection	Infectious diseases: Viral	Human Diseases	1	0.01283	NM_032484.5,
5200	Pathways in cancer	Cancers: Overview	Human Diseases	10	0.01308	NM_007182.5,NM_170712.3,NM_170713.3,NM_001206957.1,XM_011533316.2,NM_170714.2,NM_03

						2484.5,XM_005244891.5,XM_011509163.3,XM_024453328.1,
4011	MAPK signaling pathway - yeast	Signal transduction	Environmental Information Processing	3	0.01662	NM_201443.3,NM_201441.3,NM_003213.4,
4260	Cardiac muscle contraction	Circulatory system	Organismal Systems	6	0.02375	XM_005257391.5,XM_024450766.1,XM_017024683.1,NM_001002841.1,NM_002476.2,XM_011524839.2,
4623	Cytosolic DNA-sensing pathway	Immune system	Organismal Systems	5	0.02723	XM_005273356.2,XM_017002868.1,NM_001193322.2,NM_014002.4,NM_001193321.2,
4540	Gap junction	Cellular community - eukaryotes	Cellular Processes	8	0.0277	NM_001289130.1,NM_006086.4,NM_001289131.1,NM_001289129.1,NM_001289123.1,NM_001289127.1,NM_001197181.2,NM_006087.4,
4020	Calcium signaling pathway	Signal transduction	Environmental Information Processing	2	0.0295	XM_005244891.5,XM_011509163.3,
4150	mTOR signaling pathway	Signal transduction	Environmental Information Processing	1	0.03605	NM_001006665.2,
4261	Adrenergic signaling in cardiomyocytes	Circulatory system	Organismal Systems	12	0.04342	NM_001330065.1,XM_005257391.5,NM_001369869.1,NM_002708.4,XM_024450766.1,XM_006723946.2,XM_017024683.1,NM_001008709.2,NM_001002841.1,NM_206873.2,NM_002476.2,XM_011524839.2,
4622	RIG-I-like receptor	Immune	Organismal	5	0.04618	XM_005273356.2,XM_017002868.1,NM_00119332

	signaling pathway	system	Systems			2.2,NM_014002.4,NM_001193321.2,
4142	Lysosome	Transport and catabolism	Cellular Processes	1	0.05014	NM_001199058.2,
4137	Mitophagy - animal	Transport and catabolism	Cellular Processes	1	0.05069	NM_001017921.4,
4010	MAPK signaling pathway	Signal transduction	Environmental Information Processing	4	0.0535	XM_017026935.2,NM_001204284.2,NM_006247.4, NM_001006665.2,
4934	Cushing syndrome	Endocrine and metabolic diseases	Human Diseases	2	0.05496	NM_000781.3,NM_001099773.2,
5205	Proteoglycans in cancer	Cancers: Overview	Human Diseases	3	0.05596	NM_002708.4,NM_001008709.2,NM_206873.2,
4657	IL-17 signaling pathway	Immune system	Organismal Systems	5	0.05919	XM_005273356.2,XM_017002868.1,NM_00119332 2.2,NM_014002.4,NM_001193321.2,
4620	Toll-like receptor signaling pathway	Immune system	Organismal Systems	5	0.06041	XM_005273356.2,XM_017002868.1,NM_00119332 2.2,NM_014002.4,NM_001193321.2,
4144	Endocytosis	Transport and catabolism	Cellular Processes	6	0.0631	XM_005246530.3,XM_017003992.1,NM_001557.4, XM_017003990.1,XM_017003991.1,NM_00116829 8.2,
4390	Hippo signaling pathway	Signal transduction	Environmental Information Processing	13	0.06528	NM_002708.4,NM_007182.5,NM_201443.3,NM_00 1008709.2,NM_201441.3,NM_170712.3,NM_20687 3.2,NM_170713.3,NM_003213.4,NM_001206957.1,
4514	Cell adhesion molecules (CAMs)	Signaling molecules and	Environmental Information	11	0.06987	XM_011533316.2,NM_170714.2,XM_024453328.1, XM_011524889.2,XM_005276880.1,XM_01702474 1.1,XM_005276882.1,XM_017024738.1,XM_01702

		interaction	Processing			4739.1,NM_000442.5,XM_005276883.2,XM_005276881.1,XM_017024740.1,XM_011524890.1,
5226	Gastric cancer	Cancers: Specific types	Human Diseases	2	0.07614	NM_001037675.3,NM_001277444.1,
5322	Systemic lupus erythematosus	Immune diseases	Human Diseases	5	0.0885	NM_021066.3,NM_003512.4,NM_003493.3,NM_001352000.1,NM_033445.3,
4658	Th1 and Th2 cell differentiation	Immune system	Organismal Systems	5	0.09543	NM_014387.4,NM_001014988.2,NM_001014989.2,NM_001014987.2,NM_032484.5,
4610	Complement and coagulation cascades	Immune system	Organismal Systems	6	0.1365	NM_000185.4,NM_001352000.1,NM_000062.3,NM_001032295.2,XM_005244891.5,XM_011509163.3,
3018	RNA degradation	Folding, sorting and degradation	Genetic Information Processing	1	0.1393	NM_001010846.3,
4662	B cell receptor signaling pathway	Immune system	Organismal Systems	4	0.1456	NM_005428.4,NM_001258207.2,XM_005259642.1,NM_001258206.2,
5162	Measles	Infectious diseases: Viral	Human Diseases	6	0.1589	XM_005273356.2,XM_017002868.1,NM_001193322.2,NM_014002.4,NM_001193321.2,NM_032484.5,
4722	Neurotrophin signaling pathway	Nervous system	Organismal Systems	1	0.1955	NM_001006665.2,
4512	ECM-receptor interaction	Signaling molecules and interaction	Environmental Information Processing	1	0.1985	NM_001368242.1,
1200	Carbon metabolism	Global and overview maps	Metabolism	1	0.2004	NM_007088.3,
4659	Th17 cell	Immune	Organismal	5	0.2129	NM_014387.4,NM_001014988.2,NM_001014989.2,

	differentiation	system	Systems			NM_001014987.2,NM_032484.5,
4910	Insulin signaling pathway	Endocrine system	Organismal Systems	3	0.2319	NM_002708.4,NM_001008709.2,NM_206873.2,
4022	cGMP-PKG signaling pathway	Signal transduction	Environmental Information Processing	3	0.2334	NM_002708.4,NM_001008709.2,NM_206873.2,
1130	Biosynthesis of antibiotics	Global and overview maps	Metabolism	4	0.2648	NM_001172819.1,NM_002633.3,NM_007088.3,NM_001172818.1,
4972	Pancreatic secretion	Digestive system	Organismal Systems	1	0.2738	NM_005747.5,
4640	Hematopoietic cell lineage	Immune system	Organismal Systems	1	0.2759	NM_001368242.1,
4060	Cytokine-cytokine receptor interaction	Signaling molecules and interaction	Environmental Information Processing	11	0.2763	NM_005373.3,XM_005246530.3,XM_017003992.1,NM_001557.4,XM_017001320.1,XM_017003990.1,XM_017003991.1,NM_001168298.2,NM_001320037.1,NM_001320038.2,XM_017015106.2,
4974	Protein digestion and absorption	Digestive system	Organismal Systems	1	0.2815	NM_005747.5,
5168	Herpes simplex infection	Infectious diseases: Viral	Human Diseases	8	0.2824	NM_002708.4,XM_005273356.2,XM_017002868.1,NM_001008709.2,NM_001193322.2,NM_014002.4,NM_001193321.2,NM_206873.2,
4925	Aldosterone synthesis and secretion	Endocrine system	Organismal Systems	2	0.3382	NM_000781.3,NM_001099773.2,
4940	Type I diabetes mellitus	Endocrine and metabolic	Human Diseases	2	0.3384	XM_024452881.1,NM_001007089.4,

		diseases				
1210	2-Oxocarboxylic acid metabolism	Global and overview maps	Metabolism	1	0.3516	NM_007088.3,
5034	Alcoholism	Substance dependence	Human Diseases	7	0.3596	NM_021066.3,NM_002708.4,NM_001008709.2,NM_003512.4,NM_206873.2,NM_003493.3,NM_033445.3,
4720	Long-term potentiation	Nervous system	Organismal Systems	4	0.377	NM_002708.4,NM_001008709.2,NM_206873.2,NM_001006665.2,
4014	Ras signaling pathway	Signal transduction	Environmental Information Processing	11	0.3778	NM_007182.5,NM_014387.4,NM_001014988.2,NM_001014989.2,NM_170712.3,NM_170713.3,NM_001014987.2,NM_001206957.1,XM_011533316.2,NM_170714.2,XM_024453328.1,
3010	Ribosome	Translation	Genetic Information Processing	1	0.3802	NM_021029.6,
4012	ErbB signaling pathway	Signal transduction	Environmental Information Processing	1	0.3813	NM_032484.5,
4933	AGE-RAGE signaling pathway in diabetic complications	Endocrine and metabolic diseases	Human Diseases	1	0.3883	NM_032484.5,
4630	Jak-STAT signaling pathway	Signal transduction	Environmental Information Processing	3	0.3916	NM_005373.3,XM_017001320.1,NM_032484.5,
4921	Oxytocin signaling	Endocrine	Organismal	3	0.3944	NM_002708.4,NM_001008709.2,NM_206873.2,

	pathway	system	Systems			
4625	C-type lectin receptor signaling pathway	Immune system	Organismal Systems	5	0.4002	XM_005273356.2,XM_017002868.1,NM_001193322.2,NM_014002.4,NM_001193321.2,
4072	Phospholipase D signaling pathway	Signal transduction	Environmental Information Processing	8	0.4021	XM_005246530.3,XM_017003992.1,NM_001557.4,XM_017003990.1,XM_017003991.1,NM_001168298.2,XM_005244891.5,XM_011509163.3,
4015	Rap1 signaling pathway	Signal transduction	Environmental Information Processing	6	0.4029	NM_014387.4,NM_001014988.2,NM_001014989.2,NM_001014987.2,XM_005244891.5,XM_011509163.3,
4080	Neuroactive ligand-receptor interaction	Signaling molecules and interaction	Environmental Information Processing	7	0.4249	NM_002722.5,XM_011524978.3,NM_000823.4,NM_001319209.1,NM_006144.4,XM_005244891.5,XM_011509163.3,
1120	Microbial metabolism in diverse environments	Global and overview maps	Metabolism	4	0.4281	NM_001172819.1,NM_002633.3,NM_007088.3,NM_001172818.1,
5161	Hepatitis B	Infectious diseases: Viral	Human Diseases	6	0.4624	XM_005273356.2,XM_017002868.1,NM_001193322.2,NM_014002.4,NM_001193321.2,NM_032484.5,
4611	Platelet activation	Immune system	Organismal Systems	6	0.466	NM_002708.4,NM_001008709.2,NM_206873.2,NM_001368242.1,XM_005244891.5,XM_011509163.3,
4218	Cellular senescence	Cell growth and death	Cellular Processes	3	0.4997	NM_002708.4,NM_001008709.2,NM_206873.2,
4728	Dopaminergic synapse	Nervous system	Organismal Systems	3	0.5026	NM_002708.4,NM_001008709.2,NM_206873.2,
4270	Vascular smooth	Circulatory	Organismal	3	0.5068	NM_002708.4,NM_001008709.2,NM_206873.2,

	muscle contraction	system	Systems			
5133	Pertussis	Infectious diseases: Bacterial	Human Diseases	3	0.5188	NM_001352000.1,NM_000062.3,NM_001032295.2,
1230	Biosynthesis of amino acids	Global and overview maps	Metabolism	1	0.5275	NM_007088.3,
5220	Chronic myeloid leukemia	Cancers: Specific types	Human Diseases	1	0.5396	NM_032484.5,
4064	NF-kappa B signaling pathway	Signal transduction	Environmental Information Processing	4	0.5572	NM_014387.4,NM_001014988.2,NM_001014989.2, NM_001014987.2,
5164	Influenza A	Infectious diseases: Viral	Human Diseases	5	0.6121	XM_005273356.2,XM_017002868.1,NM_001193322.2,NM_014002.4,NM_001193321.2,
5160	Hepatitis C	Infectious diseases: Viral	Human Diseases	5	0.6241	XM_005273356.2,XM_017002868.1,NM_001193322.2,NM_014002.4,NM_001193321.2,
4114	Oocyte meiosis	Cell growth and death	Cellular Processes	6	0.6451	NM_002708.4,NM_001008709.2,NM_001382494.1, NM_001382495.1,NM_206873.2,NM_001006665.2,
4371	Apelin signaling pathway	Signal transduction	Environmental Information Processing	6	0.667	XM_005257391.5,XM_024450766.1,XM_017024683.1,NM_001002841.1,NM_002476.2,XM_011524839.2,
5150	Staphylococcus aureus infection	Infectious diseases: Bacterial	Human Diseases	2	0.6852	NM_001926.4,NM_001352000.1,
5206	MicroRNAs in cancer	Cancers: Overview	Human Diseases	7	0.689	NM_007182.5,NM_170712.3,NM_170713.3,NM_001206957.1,XM_011533316.2,NM_170714.2,XM_024453328.1,

4913	Ovarian steroidogenesis	Endocrine system	Organismal Systems	2	0.6908	NM_000781.3,NM_001099773.2,
5163	Human cytomegalovirus infection	Infectious diseases: Viral	Human Diseases	6	0.7163	XM_005246530.3,XM_017003992.1,NM_001557.4,XM_017003990.1,XM_017003991.1,NM_001168298.2,
4024	cAMP signaling pathway	Signal transduction	Environmental Information Processing	9	0.7209	NM_005428.4,NM_002708.4,NM_001008709.2,NM_001258207.2,XM_005259642.1,NM_206873.2,NM_001258206.2,XM_005244891.5,XM_011509163.3,
4961	Endocrine and other factor-regulated calcium reabsorption	Excretory system	Organismal Systems	1	0.7299	NM_001740.5,
5221	Acute myeloid leukemia	Cancers: Specific types	Human Diseases	1	0.7329	NM_032484.5,
4917	Prolactin signaling pathway	Endocrine system	Organismal Systems	1	0.7329	NM_032484.5,
3460	Fanconi anemia pathway	Replication and repair	Genetic Information Processing	3	0.7479	XM_017023890.1,NM_152287.4,NM_001113525.2,
5031	Amphetamine addiction	Substance dependence	Human Diseases	3	0.7728	NM_002708.4,NM_001008709.2,NM_206873.2,
4927	Cortisol synthesis and secretion	Endocrine system	Organismal Systems	2	0.78	NM_000781.3,NM_001099773.2,
4931	Insulin resistance	Endocrine and metabolic	Human Diseases	4	0.7861	NM_002708.4,NM_001008709.2,NM_206873.2,NM_001006665.2,

		diseases				
3015	mRNA surveillance pathway	Translation	Genetic Information Processing	3	0.8065	NM_002708.4,NM_001008709.2,NM_206873.2,
5167	Kaposi sarcoma-associated herpesvirus infection	Infectious diseases: Viral	Human Diseases	5	0.8132	XM_005273356.2,XM_017002868.1,NM_001193322.2,NM_014002.4,NM_001193321.2,
4141	Protein processing in endoplasmic reticulum	Folding, sorting and degradation	Genetic Information Processing	4	0.8241	NM_006005.3,XM_017008586.1,NM_001145853.1,NM_003145.4,
4740	Olfactory transduction	Sensory system	Organismal Systems	6	0.8297	NM_030901.1,NM_001005218.2,NM_001004462.1,NM_001013358.2,NM_001005178.1,NM_001005236.3,
4510	Focal adhesion	Cellular community - eukaryotes	Cellular Processes	7	0.8596	NM_005428.4,NM_002708.4,NM_001008709.2,NM_001258207.2,XM_005259642.1,NM_206873.2,NM_001258206.2,
4810	Regulation of actin cytoskeleton	Cell motility	Cellular Processes	9	1	NM_005428.4,NM_002708.4,NM_001008709.2,NM_001258207.2,XM_005259642.1,NM_206873.2,NM_001258206.2,XM_005244891.5,XM_011509163.3,
3013	RNA transport	Translation	Genetic Information Processing	6	1	NM_016080.3,NM_001366248.1,NM_001366250.1,NM_001366249.1,NM_001366247.1,XM_024450778.1,
5169	Epstein-Barr virus infection	Infectious diseases: Viral	Human Diseases	5	1	XM_005273356.2,XM_017002868.1,NM_001193322.2,NM_014002.4,NM_001193321.2,
4217	Necroptosis	Cell growth	Cellular	4	1	NM_021066.3,NM_003512.4,NM_033445.3,NM_03

		and death	Processes			2484.5,
3040	Spliceosome	Transcription	Genetic Information Processing	4	1	NM_003089.6,XM_011527240.2,XM_011527241.2, NM_001301069.2,
4113	Meiosis - yeast	Cell growth and death	Cellular Processes	3	1	NM_002708.4,NM_001008709.2,NM_206873.2,
4146	Peroxisome	Transport and catabolism	Cellular Processes	3	1	XM_024452999.1,XM_024452998.1,NM_018441.6,
4391	Hippo signaling pathway - fly	Signal transduction	Environmental Information Processing	3	1	NM_201443.3,NM_201441.3,NM_003213.4,
4914	Progesterone-mediated oocyte maturation	Endocrine system	Organismal Systems	3	1	NM_001382494.1,NM_001382495.1,NM_001006665.2,
4750	Inflammatory mediator regulation of TRP channels	Sensory system	Organismal Systems	3	1	NM_002708.4,NM_001008709.2,NM_206873.2,
5014	Amyotrophic lateral sclerosis (ALS)	Neurodegenerative diseases	Human Diseases	1	1	NM_005125.2,
4975	Fat digestion and absorption	Digestive system	Organismal Systems	1	1	NM_007088.3,
4977	Vitamin digestion and absorption	Digestive system	Organismal Systems	1	1	NM_004164.3,

Table S9.1. Human genes differentially expressed in severe disease relative to mild disease in Faeces

Symptom							Gender						
GeneID	log2FC (Severe/ Mild)	logCPM	LR	*p* value	FDR	symbol	GeneID	logFC(Male/Female)	logCPM	LR	*p* value	FDR	symbol
729442	6.421490294	-0.343547427	49.92275993	1.60E-12	1.97E-09	GAGE12H	139341	-1.170310137	1.800915531	28.93813494	7.47E-08	2.89E-06	FUNDC1
653275	6.000261606	-0.236011169	39.87607988	2.71E-10	2.32E-07	CFC1B	158798	-1.140550343	1.716083508	25.99214051	3.43E-07	1.24E-05	AKAP14
729431	5.825678571	-0.815213089	48.68125872	3.01E-12	3.30E-09	GAGE12E	347468	-1.169322008	1.8183064	28.80185186	8.02E-08	3.10E-06	OR13H1
729422	5.825678571	-0.815213089	48.68125872	3.01E-12	3.30E-09	GAGE12C	728695	-1.178323175	1.844262091	28.22896426	1.08E-07	4.10E-06	SPANXB1
84218	5.791829489	-0.638474883	26.928605	2.11E-07	8.49E-05	TBC1D3F	90316	-1.048408501	1.649812565	21.01560932	4.56E-06	0.000140929	TGIF2LX
728049	4.012846091	1.65601012	156.8883746	5.41E-36	1.07E-31	CT47A8	340542	-1.368775136	1.679924733	35.35043684	2.75E-09	1.28E-07	BEX5
277	3.662874027	1.120513006	61.95487978	3.51E-15	5.33E-12	AMY1B	9363	-1.209053718	1.903640547	32.16239979	1.42E-08	5.92E-07	RAB33A
728075	2.915972784	1.306676727	76.37684827	2.34E-18	5.77E-15	CT47A4	1538	-1.0615386	2.359621955	33.48262969	7.19E-09	3.12E-07	CYLC1
728689	2.592879531	0.674252621	38.53150253	5.39E-10	3.93E-07	EIF3CL	407	-1.192728219	1.566654089	25.2633074	5.00E-07	1.75E-05	ARR3
728524	2.516529	2.03159	86.538	1.37E-	6.75E	SPDYE8P	203611	2.8673354	0.2073	35.062	3.19E-0	1.46E	CDY2B

	735	3472	97331	20	-17			74	12218	3849	9	-07	
100463289	2.349447744	8.398867601	134.5723802	4.10E-31	4.03E-27	MTRNR2L5	9452	-1.089640719	2.339314542	35.26491154	2.88E-09	1.33E-07	ITM2A
203611	2.252869487	0.207369081	19.24570074	1.15E-05	0.00263196	CDY2B	729396	-1.651320503	0.08167285	15.4348453	8.54E-05	0.002268023	GAGE12J
24150	2.203410891	0.624987418	19.30103002	1.12E-05	0.002619164	TP53TG3	259215	1.08846112	1.136990617	15.71276265	7.37E-05	0.001971269	LY6G6F
728358	2.132777476	1.471537903	38.71090863	4.91E-10	3.72E-07	DEFA1B	101928917	-1.184680139	1.586323662	25.32069659	4.85E-07	1.71E-05	HSFX3
9085	2.096476183	0.918420915	13.29461461	0.00026617	0.037638574	CDY1	728911	-1.182601024	0.378760447	9.965092207	0.001595362	0.035126482	CT45A2
100289087	2.090350377	1.905086365	28.48453306	9.44E-08	4.23E-05	TSPY10	9084	3.724389631	-0.707373748	21.72977122	3.14E-06	9.96E-05	VCY
100287399	2.020261325	0.100270988	14.81889346	0.000118344	0.019434044	POTEB2	407977	1.697771052	-0.316292045	11.07599432	0.000874525	0.019946056	TNFSF12-TNFSF13
102724101	1.957441082	2.269992785	41.2618359	1.33E-10	1.19E-07	TP53TG3E	140947	-1.342080032	1.320768334	26.37244583	2.82E-07	1.03E-05	DCANP1
100293534	1.630815792	3.181002953	67.30953791	2.32E-16	3.81E-13	C4B_2	4935	-1.06573759	2.298374157	32.74608233	1.05E-08	4.47E-07	GPR143
728419	1.564322033	3.415222685	31.98361708	1.55E-08	7.47E-06	USP17L30	170627	-1.114604793	1.541210842	22.08040875	2.61E-06	8.47E-05	XAGE5

375759	1.509583494	4.448335321	80.31811279	3.19E-19	1.05E-15	C9orf50	28986	-1.03479662	2.448130467	34.23265627	4.89E-09	2.19E-07	MAGEH1
3126	1.458080089	1.413881138	16.03361283	6.22E-05	0.011681462	HLA-DRB4	353143	-1.733466544	-0.145456457	14.14827943	0.000168952	0.004279382	LCE3B
100463482	1.445636855	9.91946	51.63823827	6.67E-13	8.77E-10	MTRNR2L6	494118	-1.280106529	1.613919596	30.04601764	4.22E-08	1.67E-06	SPANXN1
1617	1.218911501	2.259088341	15.34728984	8.94E-05	0.015540574	DAZ1	101929627	-1.526573319	0.620046951	13.2911164	0.000266667	0.006614389	LOC101929627
100463285	1.198463677	7.13264508	38.98258113	4.28E-10	3.37E-07	MTRNR2L4	728096	-1.11603392 3	2.027574281	25.61039473	4.18E-07	1.49E-05	CT47A1
100463488	1.193981752	10.64583246	34.615701	4.02E-09	2.20E-06	MTRNR2L10	90737	-1.160303212	1.480243491	23.19314968	1.47E-06	4.88E-05	PAGE5
343066	1.189929678	4.313211606	47.64687208	5.10E-12	5.03E-09	AADACL4	142689	-1.040538572	2.109194669	27.78503344	1.36E-07	5.13E-06	ASB12
1667	1.122013511	2.004047923	13.24496259	0.000273313	0.038197966	DEFA1	110806299	-1.329736001	-0.03843176	9.691222898	0.001851503	0.040494687	ETDC
83694	1.091444267	5.953455972	33.46246139	7.27E-09	3.77E-06	RPS6KL1	26609	-1.206608862	1.386396698	22.91836543	1.69E-06	5.59E-05	VCX
728369	1.06715336	3.380574128	25.49980489	4.42E-07	0.000161453	USP17L24	100129239	-1.175318296	1.283987259	20.57623199	5.73E-06	0.000174531	CXorf51A
645402	1.056766384	2.391080935	17.69586784	2.59E-05	0.0054920	USP17L4	727940	-1.311208656	0.602767809	14.9326971	0.000111415	0.0029118	RHOXF2B

					21							66	
84798	1.047805182	5.150947147	39.47429567	3.32E-10	2.73E-07	C19orf48	56849	-1.018746842	2.056567391	25.93051322	3.54E-07	1.28E-05	TCEAL7
100462983	1.026515431	5.103628839	28.96160288	7.38E-08	3.38E-05	MTRNR2L3	494119	-1.341206544	0.984997133	21.88868217	2.89E-06	9.26E-05	SPANXN2
440243	1.026304267	4.631818626	35.11888198	3.10E-09	1.85E-06	GOLGA6L22	100287441	-1.046957856	2.480560739	24.34217902	8.07E-07	2.75E-05	USP17L20
100462981	1.014807909	14.28442467	22.33462113	2.29E-06	0.000739911	MTRNR2L2	553158	1.62379019	0.370822401	19.30271925	1.12E-05	0.000325652	PRR5-ARHGAP8
54065	1.008947547	4.15518104	36.60492776	1.45E-09	9.48E-07	SMIM11A	6658	-1.009466777	2.036370675	24.66048778	6.84E-07	2.35E-05	SOX3
399939	-1.071023269	2.988376578	21.38668313	3.75E-06	0.001087798	TRIM49D1	100526760	1.697792038	0.221936308	17.85289147	2.39E-05	0.000679619	ABHD14A-ACY1
7298	-1.120462912	2.302891046	15.77336285	7.14E-05	0.013149743	TYMS	102724334	-2.593889876	-0.657740445	13.69883188	0.000214588	0.0054006	LOC102724334
79057	-1.126669605	5.425227949	34.67047241	3.91E-09	2.20E-06	PRRG3	645073	-1.071702408	1.735646839	23.5219307	1.23E-06	4.15E-05	GAGE12G
1493	-1.149713045	7.43276613	23.38186322	1.33E-06	0.000436242	CTLA4	729355	-1.057474954	1.576955941	19.70409693	9.04E-06	0.000268785	TP53TG3B
100532731	-1.161009144	2.050913952	15.14642381	9.95E-05	0.016614258	COMMD3-BMI1	503614	-1.936314895	-0.372652971	12.32069987	0.000447963	0.01068711	DEFB107B

221786	-1.195203524	6.161601734	33.97264743	5.59E-09	2.98E-06	FAM200A	5956	-1.160742859	1.552875148	23.02582169	1.60E-06	5.29E-05	OPN1LW
100996928	-1.198745252	2.144597899	15.27260672	9.31E-05	0.0159458	FMC1-LUC7L2	343070	1.321419056	0.388583883	12.87079865	0.000333749	0.00817243	PRAMEF9
548644	-1.302420725	3.694784204	36.3220961	1.67E-09	1.03E-06	POLR2J3	392465	-1.000261199	2.017797323	24.47876769	7.51E-07	2.57E-05	GLOD5
100996413	-1.338416335	2.303029835	17.04906754	3.64E-05	0.007636339	LOC100996413	3047	1.236272731	0.710816343	14.90908713	0.000112818	0.002940736	HBG1
100526794	-1.351616611	1.630665571	13.6095565	0.000225037	0.032848741	NT5C1B-RDH14	58530	1.605409894	-0.344864642	9.97391622	0.001587734	0.034997641	LY6G6D
100506164	-1.368373298	2.637327979	22.0607371	2.64E-06	0.000839596	HSFX1	347411	-1.275829426	0.943765438	18.8799276	1.39E-05	0.000403422	MPC1L
100653049	-1.383607866	2.18233889	15.3377314	8.99E-05	0.015540574	LOC100653049	245909	-1.058511423	0.929158398	13.32735498	0.000261562	0.006499804	DEFB106A
26168	-1.399111527	7.362387647	33.00760625	9.18E-09	4.52E-06	SENP3	548593	1.002706267	1.996215719	22.18744826	2.47E-06	8.05E-05	SLX1A
100287364	-1.430167229	3.499214103	37.49450435	9.17E-10	6.32E-07	USP17L18	147199	-1.062882905	1.922162443	25.23678632	5.07E-07	1.77E-05	SCGB1C1
8073	-1.473749478	9.07894584	47.9313457	4.41E-12	4.58E-09	PTP4A2	282808	-1.183683697	1.746789655	28.16883601	1.11E-07	4.22E-06	RAB40AL
11038469	-1.50748	3.06060	27.475	1.59E-	6.67E	LOC11038	10012940	-1.7502577	0.4120	21.352	3.82E-0	0.000	FAM236A

2	2235	6475	26116	07	-05	4692	7	61	55322	18865	6	119545	
100526693	-1.601278832	2.810614451	33.27587599	8.00E-09	4.04E-06	ARPC4-TTLL3	425054	-1.024854988	1.821172693	22.66562867	1.93E-06	6.34E-05	VCX3B
728137	-1.624516231	2.350640594	21.4203136	3.69E-06	0.001084838	TSPY3	203562	-1.020246556	1.490021755	18.04027	2.16E-05	0.000617687	TMEM31
4108	-1.778761299	2.471507643	26.35003929	2.85E-07	0.00011005	MAGEA9	23676	-1.008454374	1.732496747	20.65554848	5.50E-06	0.00016828	SMPX
1668	-1.865172363	1.604045786	23.83056966	1.05E-06	0.000357421	DEFA3	100533997	-1.0210362	1.717110547	20.1910828	7.01E-06	0.000211158	MAGEA10-MAGEA5
100526737	-1.911784401	1.484468576	16.57763759	4.67E-05	0.009542505	RBM14-RBM4	158800	-1.050251046	1.107921293	14.58083623	0.000134273	0.003458806	RHOXF1
721	-1.915994068	3.148437906	52.64809167	3.99E-13	5.62E-10	C4B	90843	-1.074543357	2.216724379	31.91832237	1.61E-08	6.67E-07	TCEAL8
147199	-2.038688963	1.922050162	23.69727148	1.13E-06	0.000376559	SCGB1C1	8363	-3.553590933	-0.692211911	19.72987507	8.92E-06	0.000265915	H4C11
402317	-2.084967061	1.39874095	19.85982234	8.33E-06	0.002105345	OR2A42	552891	1.593300824	0.358156827	17.59450057	2.73E-05	0.000764139	DNAJC25-GNG10
4104	-2.145730854	1.230300088	18.23989552	1.95E-05	0.0042174	MAGEA5	728458	-1.895438728	-0.13443199	15.14134343	9.98E-05	0.0026315	OPN1MW2

					38				2			46	
100528020	-2.345926811	0.327290994	14.24508455	0.000160479	0.025299256	FAM187A	284428	-1.048809415	0.762516784	11.04362337	0.000889928	0.020273888	MBD3L5
414060	-2.485834094	1.807040814	30.25221276	3.79E-08	1.78E-05	TBC1D3C	107303344	3.766124643	-0.725482044	21.92249291	2.84E-06	9.13E-05	SETDB2-PHF11
728393	-2.485923434	3.564587647	85.5040245	2.31E-20	9.11E-17	USP17L27	2664	-1.004005401	2.56287825	34.5066426	4.25E-09	1.91E-07	GDI1
728945	-2.828253671	0.983215538	21.72856404	3.14E-06	0.000967071	PPIAL4F	3028	-1.03863739	1.496393526	18.85962336	1.41E-05	0.00040714	HSD17B10
730262	-2.828253671	0.983215538	21.72856404	3.14E-06	0.000967071	PPIAL4E	8270	-1.171152588	1.116713309	18.48455021	1.71E-05	0.000491323	LAGE3
653404	-3.066591722	1.726884718	45.04278746	1.93E-11	1.81E-08	FOXD4L6	51442	-1.08667216	1.959165112	27.73575476	1.39E-07	5.23E-06	VGLL1
728405	-3.199760199	2.928267127	27.11339613	1.92E-07	7.88E-05	USP17L29	1476	1.007558058	1.842322559	21.20896231	4.12E-06	0.000128411	CSTB
728712	-3.276689562	0.331771375	26.0655189	3.30E-07	0.000122707	SPANXA2	3266	-1.125605472	1.380692954	20.59079021	5.69E-06	0.000173477	ERAS
54921	-3.286903996	2.247274936	72.23678193	1.91E-17	3.76E-14	CHTF8	51270	-1.020601818	2.482581301	33.4112653	7.46E-09	3.22E-07	TFDP3
284428	-4.22406	0.76251	27.873	1.30E-	5.55E	MBD3L5	728689	1.6788791	0.6741	21.537	3.47E-0	0.000	EIF3CL

	9183	8334	5692	07	-05			73	6504	63527	6	10887	
100129407	-4.235533824	0.412014878	12.99000839	0.000313158	0.042854743	FAM236A	101060233	1.715210411	-0.45000016	9.414073908	0.002153263	0.046424724	OPN1MW3
105180390	-4.570901385	0.079396735	14.91306014	0.000112581	0.018643017	SPDYE13P	112267876	-2.0289399	-0.555895704	11.39275033	0.000737313	0.017133829	LOC112267876
100533496	-4.670501976	-0.161560974	16.35528695	5.25E-05	0.010347517	TVP23C-CDRT4	100529144	2.03734747	-0.351324778	14.15353121	0.000168481	0.004278451	CORO7-PAM16
728090	-4.740519262	2.406246603	70.24144726	5.25E-17	9.40E-14	CT47A2	653067	-1.030786634	2.390721573	32.87437125	9.83E-09	4.20E-07	XAGE1B
245908	-5.250913328	0.269116489	23.86203167	1.03E-06	0.000357421	DEFB105A	728082	1.333417849	1.232059482	20.10066672	7.35E-06	0.000221041	CT47A3
150094	-5.380746484	1.132837058	27.96946239	1.23E-07	5.40E-05	SIK1	158511	-1.153760213	1.381828676	21.70616816	3.18E-06	0.000100674	CSAG1
100287441	-5.955340997	2.480439533	36.54553461	1.49E-09	9.48E-07	USP17L20	10549	-1.263858372	1.331004367	24.68559911	6.75E-07	2.32E-05	PRDX4
105180391	-6.10554192	0.78183056	34.85289483	3.56E-09	2.06E-06	SPDYE15P	3598	-1.029995067	1.871000859	23.66837533	1.14E-06	3.86E-05	IL13RA2
653656	-6.325718377	0.644458371	37.46747706	9.30E-10	6.32E-07	MBD3L4	795	-1.026442107	2.006722261	25.12196878	5.38E-07	1.87E-05	S100G
728042	-7.06702	2.17936	77.568	1.28E-	3.61E	CT47A9	353513	2.6146279	0.1144	33.885	5.84E-0	2.57E	VCY1B

	455	9121	14487	18	-15			03	57623	91272	9	-07	
101060301	-7.265613038	-0.777976066	19.69139196	9.10E-06	0.002270191	HNRNPCL4	100527949	-1.170262298	1.030689423	16.10124662	6.00E-05	0.001623096	GIMAP1-GIMAP5
102724127	-8.600429903	2.287544846	129.5902565	5.04E-30	3.31E-26	TP53TG3F	729447	-1.076706934	2.427849749	35.10469136	3.12E-09	1.44E-07	GAGE2A
255313	-9.438254693	0.925098046	75.98448164	2.86E-18	6.26E-15	CT47A11	645051	-2.765050604	-0.021697376	33.43993314	7.35E-09	3.18E-07	GAGE13
							100289087	7.933974175	1.905009822	306.4526894	1.29E-68	1.59E-65	TSPY10
							728403	6.519751346	1.459542806	205.7627153	1.15E-46	9.48E-44	TSPY8
							57135	6.450555936	2.270993892	307.4368256	7.90E-69	1.04E-65	DAZ4
							9085	6.397595319	0.918489458	104.9294938	1.27E-24	5.67E-22	CDY1
							57055	5.560185868	2.852632665	459.2830894	6.88E-102	1.51E-98	DAZ2
							126961	5.393888516	-0.177254111	60.05611037	9.22E-15	1.28E-12	H3C14
							378949	5.310001903	2.632110805	417.8203381	7.28E-93	1.30E-89	RBMY1D
							159119	5.104746772	1.833815128	231.4111162	2.93E-52	2.89E-49	HSFY2

	5.1033370	1.7402	221.42	4.41E-5	3.95E	
64591	19	40888	98385	0	-47	TSPY2
	5.0860693	0.4657	83.715	5.71E-2	1.94E	
5940	45	29856	08368	0	-17	RBMY1A1
	5.0139233	3.0340	491.90	5.48E-1	1.54E	
7258	07	66923	88116	09	-105	TSPY1
	4.9921384	3.6984	679.69	7.79E-1	5.12E	
9081	27	45349	32537	50	-146	PRY
	4.9653687	1.9291	252.89	6.08E-5	6.31E	
9426	48	31627	24444	7	-54	CDY2A
	4.9036563	3.8670	732.94	2.05E-1	2.47E	
728395	35	05727	77353	61	-157	TSPY4
	4.8682670	1.0315	133.34	7.60E-3	4.40E	
353515	01	9012	51843	1	-28	XKRY2
	4.8682670	1.0315	133.34	7.60E-3	4.40E	
9082	01	9012	51843	1	-28	XKRY
	4.8268589	0.9803	128.10	1.06E-2	5.82E	
9086	57	97446	81893	9	-27	EIF1AY
	4.5972700	4.0674	732.54	2.51E-1	2.47E	
159163	92	41002	35438	61	-157	RBMY1F
	4.5463692	1.2041	145.33	1.82E-3	1.23E	
9087	61	9262	1885	3	-30	TMSB4Y
	4.5366670	0.5230	88.575	4.89E-2	1.79E	
6736	06	41704	08655	1	-18	SRY
	4.4116571	3.3171	512.74	1.60E-1	7.90E	
8287	97	96862	38625	13	-110	USP9Y

	4.4081103	0.8420	108.52	2.07E-2	9.70E	
442867	96	29926	1152	5	-23	BPY2B
	4.4081103	0.8420	108.52	2.07E-2	9.70E	
442868	96	29926	1152	5	-23	BPY2C
	4.4081103	0.8420	108.52	2.07E-2	9.70E	
9083	96	29926	1152	5	-23	BPY2
	4.3598093	0.1848	64.797	8.30E-1	1.47E	
266	06	88277	4534	6	-13	AMELY
	4.2461165	3.2670	486.51	8.17E-1	2.01E	
253175	39	16369	38601	08	-104	CDY1B
	4.1739877	3.4086	510.49	4.94E-1	1.95E	
378950	69	59022	84235	13	-109	RBMY1E
	4.0427083	1.3607	137.40	9.84E-3	6.25E	
86614	39	32127	40526	2	-29	HSFY1
	3.9126534	2.6489	316.05	1.05E-7	1.47E	
7544	44	16789	72673	0	-67	ZFY
	3.9001464	0.7511	91.455	1.14E-2	4.33E	
90655	62	49293	48952	1	-19	TGIF2LY
	3.6990133	3.1831	405.55	3.40E-9	5.59E	
378951	83	13616	48855	0	-87	RBMY1J
	3.5073344	2.2594	216.55	5.10E-4	4.37E	
1617	09	33251	78489	9	-46	DAZ1
	3.4347479	1.0404	72.952	1.33E-1	3.27E	
378948	01	11916	86014	7	-15	RBMY1B
	3.4210473	2.3504	224.10	1.15E-5	1.08E	
728137	1	01537	72834	0	-47	TSPY3

	3.3163677	4.0605	448.59	1.46E-9	2.87E	
728400	59	65475	56512	9	-96	USP17L28
	3.2130680	0.2746	51.603	6.79E-1	6.00E	
140032	39	45214	73227	3	-11	RPS4Y2
	3.1754432	3.4104	367.38	6.95E-8	1.05E	
8284	31	31562	34691	2	-78	KDM5D
	3.1411146	1.0474	85.920	1.87E-2	6.48E	
6192	19	58921	3042	0	-18	RPS4Y1
	3.1297689	2.7909	263.30	3.27E-5	3.79E	
8653	45	80464	32158	9	-56	DDX3Y
	3.0598443	4.6824	493.89	2.03E-1	6.66E	
22829	6	12708	21387	09	-106	NLGN4Y
10518039	3.0426958	0.7818	65.578	5.58E-1	1.05E	
1	01	10241	4534	6	-13	SPDYE15P
	2.6906187	3.1813	262.21	5.66E-5	6.20E	
90665	88	40036	02438	9	-56	TBL1Y
	2.5924570	0.3317	37.817	7.77E-1	3.91E	
728712	9	02178	93659	0	-08	SPANXA2
	2.5535912	1.4308	74.115	7.37E-1	1.93E	
445329	99	58556	78082	8	-15	SULT1A4
	2.3435309	0.4219	36.211	1.77E-0	8.53E	
442862	57	22571	03338	9	-08	PRY2
10028747	2.0392590	2.8782	119.71	7.30E-2	3.89E	
8	73	64198	6561	8	-25	USP17L21
10046348	1.9392315	13.560	156.64	6.11E-3	4.63E	
6	59	66796	8607	6	-33	MTRNR2L8

100463482	1.897297189	9.919454889	151.7458218	7.20E-35	5.26E-32	MTRNR2L6
100463488	1.858354038	10.64583105	150.7574552	1.18E-34	8.33E-32	MTRNR2L10
100462981	1.833263667	14.28442463	141.1907234	1.46E-32	9.60E-30	MTRNR2L2
100462977	1.725164282	11.73183256	134.541298	4.16E-31	2.56E-28	MTRNR2L1
728929	1.647648419	3.156417299	101.0949066	8.77E-24	3.68E-21	ELOA3B
102724127	1.427984261	2.287449855	49.56782811	1.92E-12	1.56E-10	TP53TG3F
100463289	1.301190198	8.398851346	81.3494523	1.89E-19	6.21E-17	MTRNR2L5
100463487	1.136954597	8.228626391	58.42884627	2.11E-14	2.61E-12	MTRNR2L9
100293534	1.073002219	3.180977016	47.08015781	6.81E-12	4.99E-10	C4B_2
6282	1.044689553	2.842181557	41.50986382	1.17E-10	6.66E-09	S100A11
90293	-1.000297848	4.234732021	67.42466	2.19E-16	4.49E-14	KLHL13
4110	-1.001805944	3.738172354	59.89428435	1.00E-14	1.38E-12	MAGEA11
442444	-1.003220063	3.407765677	52.7912588	3.71E-13	3.43E-11	FAM47C

	-1.0038739	2.8459	40.016	2.52E-1	1.36E	
2277	55	75556	06839	0	-08	VEGFD
	-1.0044749	5.4054	64.451	9.89E-1	1.70E	
79868	59	16965	43288	6	-13	ALG13
	-1.0050173	2.8580	40.490	1.98E-1	1.10E	
170062	79	75277	94628	0	-08	FAM47B
	-1.0064449	4.5098	69.815	6.51E-1	1.41E	
139324	47	26133	08748	7	-14	HDX
	-1.0066208	3.3531	51.231	8.21E-1	7.19E	
23708	16	88477	11766	3	-11	GSPT2
10013030	-1.0067787	3.5121	54.815	1.32E-1	1.37E	
2	26	54913	72533	3	-11	SUPT20HL1
	-1.0075970	3.6468	57.772	2.94E-1	3.49E	
114928	12	1325	0542	4	-12	GPRASP2
		4.8986	69.732	6.79E-1	1.45E	
139065	-1.008342	02721	81401	7	-14	SLITRK4
	-1.0086485	2.6379	36.167	1.81E-0	8.70E	
158506	96	59784	25515	9	-08	CBLL2
	-1.0097041	3.6755	58.394	2.15E-1	2.64E	
7569	06	12552	2463	4	-12	ZNF182
	-1.0097683	4.6756	70.761	4.03E-1	9.13E	
412	09	33563	79562	7	-15	STS
	-1.0118065	3.1964	48.432	3.42E-1	2.63E	
4068	17	51849	64919	2	-10	SH2D1A
	-1.0133508	2.8658	41.458	1.20E-1	6.82E	
2245	72	25638	0433	0	-09	FGD1

	-1.0138300	4.6904	71.702	2.50E-1	5.94E	
139189	05	65123	56956	7	-15	DGKK
	-1.0139776	3.9695	65.876	4.80E-1	9.19E	
84295	89	05368	28602	6	-14	PHF6
	-1.0146974	3.3845	53.269	2.91E-1	2.75E	
1831	87	339	84666	3	-11	TSC22D3
	-1.0164727	3.5626	57.744	2.99E-1	3.50E	
59272	87	38153	0953	4	-12	ACE2
	-1.0167492	3.8340	62.993	2.07E-1	3.19E	
4168	02	59525	91319	5	-13	MCF2
	-1.0168185	4.2649	69.924	6.16E-1	1.35E	
56062	36	56705	19476	7	-14	KLHL4
	-1.0169243	3.4699	54.783	1.35E-1	1.39E	
139420	61	74601	45172	3	-11	PPP4R3C
	-1.0172007	3.0487	46.639	8.53E-1	6.03E	
5634	9	28083	55775	2	-10	PRPS2
	-1.0193069	4.1469	68.663	1.17E-1	2.47E	
401613	76	4435	82458	6	-14	SERTM2
	-1.0200317	3.6431	59.590	1.17E-1	1.57E	
6103	67	73459	166	4	-12	RPGR
	-1.0206037	3.3679	52.907	3.50E-1	3.28E	
158833	29	84489	3695	3	-11	AWAT1
	-1.0207601	6.2586	59.247	1.39E-1	1.84E	
1756	31	1688	51306	4	-12	DMD
	-1.0228217	3.0649	46.352	9.88E-1	6.90E	
54440	82	21855	60423	2	-10	SASH3

	-1.0261080	3.3564	53.723	2.31E-1	2.25E	
5303	33	47616	89264	3	-11	PIN4
	-1.0278432	4.4368	73.524	9.94E-1	2.48E	
117154	79	93522	8656	8	-15	DACH2
	-1.0284090	4.2388	71.165	3.29E-1	7.62E	
51114	8	2143	03796	7	-15	ZDHHC9
	-1.0295780	4.8208	73.667	9.24E-1	2.34E	
4534	71	56819	57128	8	-15	MTM1
	-1.0301894	3.1321	48.684	3.01E-1	2.35E	
1193	45	05981	75051	2	-10	CLIC2
	-1.0327620	4.1704	70.989	3.59E-1	8.23E	
9949	53	16715	63141	7	-15	AMMECR1
	-1.0328434	4.1913	70.010	5.90E-1	1.31E	
3750	72	54153	23586	7	-14	KCND1
	-1.0330228	5.4841	66.389	3.70E-1	7.15E	
53344	08	65922	24335	6	-14	CHIC1
	-1.0355915	5.0253	72.287	1.86E-1	4.47E	
2157	88	83958	61337	7	-15	F8
	-1.0379714	4.1811	70.719	4.12E-1	9.22E	
91851	31	90144	89776	7	-15	CHRDL1
	-1.0384298	4.9401	74.099	7.43E-1	1.93E	
83550	98	50508	17798	8	-15	GPR101
	-1.0429343	2.6452	38.375	5.84E-1	2.99E	
4693	01	26799	5766	0	-08	NDP
	-1.0429438	3.4375	56.508	5.60E-1	6.23E	
55511	29	63649	06325	4	-12	SAGE1

	-1.0429935	2.9380	45.392	1.61E-1	1.06E	
56850	1	07329	00192	1	-09	GRIPAP1
	-1.0454907	4.3633	74.971	4.78E-1	1.33E	
286410	15	03595	46743	8	-15	ATP11C
	-1.0472621	3.6937	63.831	1.36E-1	2.21E	
2556	68	90257	19151	5	-13	GABRA3
	-1.0476080	3.5752	60.759	6.45E-1	9.41E	
116442	51	58221	88764	5	-13	RAB39B
	-1.0487953	3.3160	55.258	1.06E-1	1.11E	
186	48	59751	93731	3	-11	AGTR2
	-1.0517263	3.5194	60.186	8.63E-1	1.22E	
9075	51	3212	66984	5	-12	CLDN2
	-1.0537182	4.2463	74.341	6.57E-1	1.75E	
3547	03	94	96235	8	-15	IGSF1
	-1.0541723	2.5515	37.576	8.79E-1	4.39E	
8823	71	69183	36072	0	-08	FGF16
10012951	-1.0544334	4.0050	71.522	2.74E-1	6.43E	
5	06	57762	21783	7	-15	ETDB
	-1.0568882	3.6394	63.542	1.57E-1	2.49E	
83604	43	17378	13308	5	-13	TMEM47
	-1.0583777	3.1021	50.681	1.09E-1	9.35E	
9104	52	67294	29727	2	-11	RGN
	-1.0586674	3.7713	66.878	2.89E-1	5.69E	
54466	73	83916	82532	6	-14	SPIN2A
	-1.0605443	4.2047	75.169	4.32E-1	1.22E	
9767	03	24204	24461	8	-15	JADE3

	-1.0643326	5.5993	68.154	1.51E-1	3.17E	
2334	17	77514	2922	6	-14	AFF2
	-1.0654034	2.6941	41.903	9.59E-1	5.54E	
10857	96	52348	98495	1	-09	PGRMC1
	-1.0659405	3.0578	50.482	1.20E-1	1.03E	
10813	66	80519	59677	2	-10	UTP14A
	-1.0690913	3.6456	64.493	9.68E-1	1.67E	
171484	55	23524	63881	6	-13	FAM9C
	-1.0728487	4.2956	78.304	8.83E-1	2.72E	
90167	46	13516	03305	9	-16	FRMD7
	-1.0788394	2.6939	42.573	6.81E-1	4.01E	
170685	34	86362	0602	1	-09	NUDT10
	-1.0792728	4.4787	80.046	3.66E-1	1.18E	
286499	77	46603	31048	9	-16	FAM133A
	-1.0805292	3.1366	53.936	2.07E-1	2.04E	
4129	49	33425	0955	3	-11	MAOB
	-1.0864482	3.2617	57.881	2.78E-1	3.34E	
4675	11	84316	72065	4	-12	NAP1L3
	-1.0867342	4.0765	76.109	2.68E-1	7.66E	
85417	67	66034	68667	8	-16	CCNB3
10537324	-1.0906423	3.4394	62.422	2.77E-1	4.20E	LOC1053732
2	95	13209	2608	5	-13	42
	-1.0943938	4.1329	77.726	1.18E-1	3.59E	
26280	4	05839	77327	8	-16	IL1RAPL2
	-1.1026004	3.9968	77.501	1.33E-1	3.96E	
139221	03	70093	41811	8	-16	PWWP3B

	-1.1041098	2.8507	48.955	2.62E-1	2.10E	
660	83	09358	78406	2	-10	BMX
	-1.1042241	2.4127	37.096	1.12E-0	5.53E	
139741	99	191	29819	9	-08	ACTRT1
	-1.1054430	3.9945	77.147	1.59E-1	4.60E	
139716	84	15597	20277	8	-16	GAB3
	-1.1061888	3.0820	54.979	1.22E-1	1.27E	
8277	6	76285	62926	3	-11	TKTL1
	-1.1082468	2.6746	44.711	2.28E-1	1.43E	
50814	2	45017	91424	1	-09	NSDHL
	-1.1104697	2.9817	54.015	1.99E-1	1.98E	
548313	98	2594	75549	3	-11	SSX4B
10537329	-1.1112459	3.4207	63.493	1.61E-1	2.54E	
7	54	35371	44049	5	-13	ERVFC1
	-1.1134252	4.2563	82.845	8.87E-2	2.96E	
203522	23	29576	27912	0	-17	INTS6L
	-1.1146372	2.4973	40.286	2.19E-1	1.20E	
51213	85	15377	88489	0	-08	LUZP4
	-1.1149690	3.8486	74.838	5.11E-1	1.40E	
158747	35	34159	74722	8	-15	MOSPD2
	-1.1171708	3.1495	57.008	4.34E-1	4.97E	
2652	85	18103	54371	4	-12	OPN1MW
10099664	-1.1206785	3.4681	65.555	5.65E-1	1.05E	
8	92	99792	031	6	-13	TCP11X2
	-1.1232621	7.3619	44.908	2.07E-1	1.32E	
26168	76	03973	00377	1	-09	SENP3

	-1.1272378	3.1237	58.123	2.46E-1	2.98E	
158724	11	64426	54175	4	-12	FAM47A
	-1.1346231	6.1616	60.670	6.75E-1	9.71E	
221786	31	48509	31075	5	-13	FAM200A
	-1.1372228	2.3067	37.251	1.04E-0	5.13E	
347487	33	88691	07066	9	-08	CXorf66
	-1.1581343	2.8986	55.067	1.16E-1	1.22E	
51438	46	20313	86137	3	-11	MAGEC2
	-1.1596577	4.0320	86.353	1.50E-2	5.30E	
2239	57	2068	56482	0	-18	GPC4
	-1.1661208	2.3244	39.798	2.82E-1	1.51E	
54830	62	82917	35861	0	-08	NUP62CL
	-1.1704795	4.1251	89.153	3.65E-2	1.36E	
203523	98	49174	16149	1	-18	ZNF449
	-1.1744250	2.5888	47.368	5.88E-1	4.39E	
29935	68	90299	48148	2	-10	RPA4
	-1.1796971	4.5628	95.020	1.88E-2	7.43E	
5251	56	63438	13552	2	-20	PHEX
10013008	-1.1809175	2.7451	48.833	2.79E-1	2.21E	
6	63	27456	5561	2	-10	HSFX2
	-1.1840401	2.1951	37.795	7.86E-1	3.95E	
254158	69	3423	23195	0	-08	CXorf58
	-1.1863524	3.0471	61.724	3.95E-1	5.94E	
8226	51	30901	04195	5	-13	PUDP
	-1.1878438	3.6588	73.999	7.81E-1	2.00E	
728379	02	8459	86363	8	-15	USP17L26

	-1.2039994	2.6688	52.012	5.51E-1	4.92E	
347516	37	19299	56133	3	-11	DGAT2L6
	-1.2156595	4.6673	101.98	5.60E-2	2.40E	
23133	55	18821	21796	4	-21	PHF8
	-1.2192327	4.3013	94.692	2.22E-2	8.59E	
84220	5	89857	83441	2	-20	RGPD5
10272368	-1.2252506	3.3242	72.397	1.76E-1	4.28E	
0	16	76282	29639	7	-15	CT45A9
		1.9831	35.362	2.74E-0	1.28E	
56271	-1.2351673	71584	83973	9	-07	BEX4
10013239	-1.2382679	2.3148	40.872	1.62E-1	9.07E	
9	17	30209	53848	0	-09	GAGE12D
	-1.2395898	3.4209	78.330	8.71E-1	2.72E	
340562	7	64575	99584	9	-16	SATL1
	-1.2444606	2.6414	54.410	1.63E-1	1.65E	
4111	11	55268	23708	3	-11	MAGEA12
	-1.2460391	2.3626	45.923	1.23E-1	8.36E	
10761	66	61886	57042	1	-10	PLAC1
10272363	-1.2466486	2.1099	38.184	6.44E-1	3.28E	
1	34	55022	73121	0	-08	CT45A10
	-1.2535323	2.4530	49.191	2.32E-1	1.88E	
10916	87	46756	33005	2	-10	MAGED2
	-1.2923164	2.2288	45.957	1.21E-1	8.24E	
1069	24	74653	58669	1	-10	CETN2
	-1.2993613	4.5768	103.47	2.63E-2	1.15E	
2742	92	16669	96766	4	-21	GLRA2

57826	-1.321817915	4.459084639	114.3224394	1.11E-26	5.74E-24	RAP2C
728090	-1.349549254	2.40637097	53.27804716	2.90E-13	2.75E-11	CT47A2
414060	-1.492094855	1.807058284	45.20728409	1.77E-11	1.15E-09	TBC1D3C
728419	-1.533659345	3.41560296	98.3657709	3.48E-23	1.43E-20	USP17L30
728405	-1.643587033	2.92898869	77.24977677	1.51E-18	4.43E-16	USP17L29
541466	-1.673761829	2.409108412	79.54517881	4.71E-19	1.50E-16	CT45A1
3188	-1.69901936	2.2137443	74.34450411	6.56E-18	1.75E-15	HNRNPH2
100507170	-1.735401969	3.929353606	175.9583206	3.70E-40	2.91E-37	CT47A12
4108	-1.827070684	2.471561247	97.55665463	5.23E-23	2.10E-20	MAGEA9
643909	-2.223018826	1.386338811	63.13767309	1.93E-15	3.01E-13	SPDYE9P
653282	-2.446104114	1.707870022	87.90071303	6.88E-21	2.47E-18	CT47A7
728036	-2.795575054	1.538866872	105.7378918	8.42E-25	3.86E-22	CT47A10
728062	-2.830997474	1.942872814	128.7228459	7.80E-30	4.39E-27	CT47A6

							105180390	-2.956194513	0.079460509	39.39900909	3.45E-10	1.84E-08	SPDYE13P
							112268355	-3.212225638	0.291526727	50.39459321	1.26E-12	1.07E-10	LOC112268355
							30014	-3.800063034	1.127676271	113.0252257	2.13E-26	1.08E-23	SPANXA1

Table S9.2. Human genes differentially expressed in severe disease relative to mild disease in NPs

Symptom							Gender						
GeneID	log2FC(Severe/Mild)	logCPM	LR	*p* value	FDR	symbol	GeneID	logFC(Male/Female)	logCPM	LR	*p* value	FDR	symbol
100526761	4.838202374	-1.206910836	23.29317038	1.39E-06	0.000409307	CCDC169-SOHLH2	728049	1.672384567	0.434893894	14.78064065	0.000120769	0.003491322	CT47A8
100463482	3.932866814	9.87729406	302.3048176	1.04E-67	2.04E-63	MTRNR2L6	102724127	-1.201690984	2.525155258	25.01878332	5.67745E-07	2.50979E-05	TP53TG3F
107987373	3.871824887	-1.10493314	17.92145533	2.30E-05	0.005403386	LOC107987373	63904	-1.01434083	1.582655844	13.44255271	0.000245981	0.006735771	DUSP21
100462977	3.64305998	12.11251487	199.6351778	2.51E-45	8.24E-42	MTRNR2L1	8544	-1.186115862	2.0360404	23.90230447	1.0135E-06	4.26061E-0	PIR

												5	
100462981	3.539496621	14.52973372	160.9996752	6.84E-37	1.50E-33	MTRNR2L2	4935	-1.103496103	2.119064314	22.78400635	1.81268E-06	7.30855E-05	GPR143
100463486	3.513329085	13.86385172	165.3403178	7.71E-38	1.90E-34	MTRNR2L8	266740	-1.089943809	2.337690256	23.35262389	1.34859E-06	5.56253E-05	MAGEA2B
100463488	3.513015416	10.55656263	228.826072	1.07E-51	5.30E-48	MTRNR2L10	6218	1.084242613	2.31653438	24.21932842	8.60E-07	3.66E-05	RPS17
102724101	3.409239401	1.682985962	132.170397	1.37E-30	2.46E-27	TP53TG3E	54145	-2.560742172	-0.151735934	22.75587985	1.8394E-06	7.39168E-05	H2BS1
401427	3.30450675	0.340292516	27.61264291	1.48E-07	6.22E-05	OR2A7	79144	1.059947496	1.939818446	17.10119448	3.54E-05	0.001138319	PPDPF
100462983	3.211542682	5.808742908	236.7245807	2.04E-53	1.34E-49	MTRNR2L3	28986	-1.026330643	2.353233988	24.90296528	6.02896E-07	2.64737E-05	MAGEH1
552891	2.673240294	-0.628597684	16.06830012	6.11E-05	0.011472512	DNAJC25-GNG10	102723737	-1.650459207	-0.155961606	13.00702931	0.000310324	0.008358395	CT45A8
100289462	2.352752296	-0.629895344	13.81955334	0.000201231	0.028749796	DEFB4B	110806299	-1.677396023	-0.308351257	10.06040735	0.001514897	0.035221362	ETDC
100463487	2.273725495	7.660149009	115.0354352	7.73E-27	1.17E-23	MTRNR2L9	414060	-1.557111004	1.238404476	22.59479019	2.00026E-06	7.95104E-0	TBC1D3C

												5	
653275	2.227807013	0.102512592	30.17762909	3.94E-08	1.85E-05	CFC1B	23630	-1.006591042	2.018406291	17.91938396	2.30462E-05	0.000758562	KCNE5
728373	2.224125563	3.684657515	201.5811913	9.44E-46	3.72E-42	USP17L25	101059915	-1.042382239	2.44086217	23.97413561	9.76386E-07	4.11334E-05	LOC101059915
23617	1.979936981	-0.385663739	16.45356584	4.99E-05	0.0097937	TSSK2	9130	-1.119752865	1.143121572	13.11403041	0.000293092	0.007915905	FAM50A
3813	1.810788334	0.500715916	23.91564538	1.01E-06	0.000310066	KIR3DS1	5956	-1.086362251	1.508043521	15.1346845	0.000100107	0.002919691	OPN1LW
100528020	1.770981217	-0.075338684	14.48655403	0.000141164	0.022105604	FAM187A	353144	1.544784321	0.070664643	10.88463696	0.000969652	0.023628558	LCE3C
100289087	1.686255215	2.125891291	31.51780435	1.98E-08	1.11E-05	TSPY10	105373314	-1.103226862	1.937857858	18.90429272	1.37443E-05	0.000473746	LOC105373314
140947	1.680544836	0.275578605	13.22100561	0.000276829	0.036386411	DCANP1	100534592	3.177518952	-1.157672545	9.711402812	0.001831282	0.041983199	URGCP-MRPS24
728369	1.597117792	3.628514672	60.23645652	8.41E-15	8.73E-12	USP17L24	101929627	4.86110337	-1.128126789	21.41881956	3.69E-06	0.000139421	LOC101929627
150094	1.572134	0.9998	20.788	5.13E-	0.001	SIK1	1005280	-1.6830639	-0.075	14.429	0.0001	0.004	FAM187A

	576	00369	99787	06	347962		20	77	500844	91826	45473	126821	
57135	1.561208539	2.734137985	53.44948184	2.65E-13	2.18E-10	DAZ4	6658	-1.128079022	2.00961781	20.98706189	4.62395E-06	0.000169769	SOX3
9085	1.459351383	0.814352166	15.96033873	6.47E-05	0.011808338	CDY1	8363	3.487458974	-0.792106244	12.28204084	0.000457338	0.011974602	H4C11
124401	1.379482233	4.25001752	75.69815979	3.31E-18	3.83E-15	ANKS3	10084	-1.054601724	2.108188588	22.0398275	2.67051E-06	0.000102836	PQBP1
445329	1.372655541	1.572678139	27.55965753	1.52E-07	6.26E-05	SULT1A4	84707	-1.149565848	1.515018767	17.03498711	3.66974E-05	0.001174554	BEX2
3188	1.358728451	1.735721595	19.9480142	7.96E-06	0.001961164	HNRNPH2	2944	-1.055296946	1.47551291	13.73475449	0.000210523	0.005788931	GSTM1
728400	1.350786709	4.065744664	61.68719957	4.03E-15	4.41E-12	USP17L28	3188	-1.23978746	1.735833388	21.65633153	3.26132E-06	0.000124132	HNRNPH2
100506164	1.33968124	2.202265194	30.0824763	4.14E-08	1.90E-05	HSFX1	343070	1.466605279	0.781729121	17.66957731	2.63E-05	0.000856412	PRAMEF9
727905	1.237561927	2.557404275	39.72057443	2.93E-10	1.81E-07	SPATA31A5	643311	-1.025745301	2.031418624	19.07363417	1.2577E-05	0.000437335	CT47B1

7730	1.220579868	1.522767399	13.24459447	0.000273367	0.036172514	ZNF177	2286	1.423133394	0.069452244	9.796884387	0.001748078	0.04021599	FKBP2
102723859	1.190100486	1.806129653	22.5675612	2.03E-06	0.000579712	TBC1D3E	1046	-1.24992248	0.987983673	14.63737541	0.000130305	0.003734134	CDX4
80823	1.178136968	5.685105384	46.73736149	8.12E-12	6.15E-09	BHLHB9	440353	4.252784841	-1.233457129	20.37753841	6.36E-06	0.000229556	NPIPB12
388324	1.134719073	5.118095745	54.89567748	1.27E-13	1.19E-10	INCA1	403244	-1.247985132	1.566133408	21.22244217	4.08948E-06	0.000152817	OR2T35
57187	1.118188727	6.422154928	48.99335295	2.57E-12	2.03E-09	THOC2	347454	-1.265658979	1.678082897	20.68574808	5.41173E-06	0.000197223	SOWAHD
125997	1.101240783	1.438590197	14.19923798	0.000164437	0.024560922	MBD3L2	11219	-1.008039381	1.272861327	10.50091676	0.001193153	0.028308316	TREX2
390075	1.091549566	1.832781895	15.8737425	6.77E-05	0.012136393	OR52N5	729355	1.196016735	0.63822637	12.10921574	0.000501732	0.013015993	TP53TG3B
100287178	1.084984507	5.093168791	41.99712387	9.14E-11	6.01E-08	USP17L11	1538	-1.036316909	2.194977887	19.3482032	1.08922E-05	0.000383482	CYLC1
51002	1.070466346	4.731634218	54.66831518	1.43E-13	1.28E-10	TPRKB	353513	3.530803962	-0.55387235	21.15302875	4.24E-06	0.0001577	VCY1B

									9			39	
23324	1.045457412	6.12049776	44.80570749	2.18E-11	1.48E-08	MAN2B2	158511	-1.304042529	1.314601347	19.64405	9.32934E-06	0.000329637	CSAG1
100293534	1.044950509	2.349247246	26.38689741	2.79E-07	0.000108024	C4B_2	80712	-1.083206712	1.706927077	16.50740148	4.84605E-05	0.001521414	ESX1
100287364	1.034807307	3.769168862	28.65797455	8.64E-08	3.78E-05	USP17L18	347549	-1.00494403	2.019762364	17.33779532	3.12901E-05	0.001016336	CENPVL3
728239	1.013861604	1.888839027	15.0472789	0.000104851	0.017371836	MAGED4	53940	-1.060551764	1.070649404	11.07261587	0.00087612	0.021619	FTHL17
56128	-1.062342348	3.751667659	39.27890683	3.67E-10	2.19E-07	PCDHB8	139760	-1.070635747	1.947642973	19.04833013	1.27449E-05	0.000442392	GPR119
105379589	-1.073485108	2.988372836	23.9426748	9.92E-07	0.000310066	LOC105379589	3598	-1.017015911	1.897909496	15.38630883	8.76209E-05	0.002590004	IL13RA2
280636	-1.09854875	4.924019738	44.97723702	1.99E-11	1.40E-08	SELENOH	11230	-1.130129748	1.77535332	19.9405303	7.98887E-06	0.000284825	PRAF2
100130086	-1.158262304	2.779430678	26.13890742	3.18E-07	0.000120464	HSFX2	100287399	-2.281192826	-0.642135483	16.16837756	5.79535E-05	0.00179374	POTEB2
728393	-1.18231	3.2707	30.710	3.00E-	1.48E	USP17L27	3808	3.6276884	-0.882	18.486	1.71E-0	0.000	KIR2DS3

	4245	2194	73617	08	-05			7	726602	71106	5	575644	
474384	-1.230486515	1.945805726	16.51578114	4.82E-05	0.009608431	F8A3	245928	1.519122209	0.493397964	13.11844991	0.000292402	0.007911091	DEFB114
102724560	-1.472375108	1.851865358	24.79572921	6.37E-07	0.000212995	CBSL	100272147	-1.251986634	1.054746186	15.846312	6.87003E-05	0.002077446	CMC4
7101	-1.503044673	5.264104717	78.57051954	7.72E-19	9.51E-16	NR2E1	474382	-1.513124896	0.91742576	22.12457078	2.55518E-06	9.89744E-05	H2AB1
100132476	-1.514956574	0.965822546	13.98537201	0.000184238	0.026709156	KRTAP4-7	7730	1.091117924	1.52312757	10.45482006	0.001223298	0.0288449	ZNF177
728137	-1.548839121	2.06808542	30.9406436	2.66E-08	1.38E-05	TSPY3	728090	-1.20206973	1.563014581	18.69515351	1.53372E-05	0.000520461	CT47A2
728082	-1.568590962	2.056207457	26.73548151	2.33E-07	9.39E-05	CT47A3	10406	1.0032252	2.168817885	16.52843636	4.79E-05	0.001507029	WFDC2
112268355	-1.580803704	0.842778361	15.47317773	8.37E-05	0.014101891	LOC112268355	729201	-2.208205639	-0.835977663	12.02236023	0.000525661	0.013618832	SPACA5B
81888	-1.653540423	0.549498641	12.52614164	0.000401298	0.0494499	HYI	653275	-1.404276093	0.102365065	9.379554994	0.002194188	0.048882048	CFC1B

644054	-1.692814711	1.454357825	22.32073463	2.31E-06	0.000649789	FAM25C	102724101	1.181033418	1.68283341	14.62183552	0.000131383	0.003759586	TP53TG3E
728929	-1.716350656	3.424124923	59.96613962	9.65E-15	9.51E-12	ELOA3B	645188	-1.532789727	1.007255571	21.25730209	4.01578E-06	0.000150523	LOC645188
504180	-1.858874613	0.672575455	17.49563623	2.88E-05	0.006308416	DEFB105B	100529144	1.694778864	0.024076889	13.4292944	0.000247725	0.006774135	CORO7-PAM16
728036	-1.870669377	1.438842214	20.29441836	6.64E-06	0.001656992	CT47A10	100132396	1.488124694	0.262828997	14.03302248	0.000179628	0.004995134	ZNF705B
147199	-1.878013428	1.593368768	24.5133457	7.38E-07	0.000242497	SCGB1C1	84218	2.461563482	-0.637555406	15.73266243	7.30E-05	0.002192609	TBC1D3F
2952	-1.930212996	3.46629359	100.4113236	1.24E-23	1.74E-20	GSTT1	170627	-1.361606569	1.565166792	22.52577284	2.07343E-06	8.17595E-05	XAGE5
100529144	-1.934332679	0.02424053	12.61236806	0.000383203	0.047664357	CORO7-PAM16	3127	-1.191983628	1.103322613	9.616132332	0.001928755	0.044013118	HLA-DRB5
728419	-1.972777166	3.696194944	92.10531545	8.22E-22	1.08E-18	USP17L30	9452	-1.048585082	2.257971307	22.42374142	2.18654E-06	8.54539E-05	ITM2A
1617	-2.106823241	1.814129975	46.24418125	1.04E-11	7.62E-09	DAZ1	56000	-1.004682325	2.185083977	20.23130616	6.86207E-06	0.0002464	NXF3

												35	
728403	-2.116278775	1.278719041	24.1821383	8.76E-07	0.000278699	TSPY8	10682	-1.065183079	1.601364629	15.57246933	7.94024E-05	0.002361232	EBP
110599583	-2.163352004	0.649395032	24.37689175	7.92E-07	0.000256027	EEF1AKMT4-ECE2	494197	-1.187725143	1.210029579	15.59981884	7.82621E-05	0.002330839	SPANXN5
102724770	-2.335394601	0.37598577	15.20599058	9.64E-05	0.016106491	LOC102724770	641654	1.093190062	1.292449664	11.14068651	0.000844546	0.020892189	HEPN1
100287327	-2.351678554	4.417893773	144.2633292	3.11E-33	6.14E-30	USP17L17	441525	-1.438390069	1.251299461	22.75442496	1.8408E-06	7.39168E-05	SPANXN4
346528	-2.605517511	0.906998366	31.14821368	2.39E-08	1.31E-05	OR2A1	6156	1.102873706	2.01954979	18.75917847	1.48E-05	0.000505889	RPL30
728945	-2.607792373	0.670473328	25.4157127	4.62E-07	0.000159851	PPIAL4F	100132285	1.104107473	1.407026753	16.07902017	6.08E-05	0.001868664	KIR2DS2
730262	-2.607792373	0.670473328	25.4157127	4.62E-07	0.000159851	PPIAL4E	441490	-1.07639225	1.73203309	16.15032014	5.85087E-05	0.001808083	FTHL18
353143	-2.719379628	-0.412111028	14.97172904	0.000109134	0.017718463	LCE3B	203569	-1.5794422	0.192218598	10.92238935	0.00095009	0.023211863	PAGE2
728405	-2.74996	4.0153	182.05	1.73E-	4.86E	USP17L29	24140	-1.0495028	2.3347	23.437	1.2904	5.333	FTSJ1

	8049	90002	2874	41	-38			53	21862	37009	7E-06	95E-05	
653282	-2.929347873	1.789880462	53.98727917	2.02E-13	1.73E-10	CT47A7	730394	-1.143353958	1.990990392	21.05179372	4.47034E-06	0.000164435	GTF2H2C_2
353144	-3.004351359	0.070709382	23.55166858	1.22E-06	0.000368853	LCE3C	3421	-1.01757173	1.711865209	14.94730789	0.000110556	0.00321492	IDH3G
57054	-3.189427387	0.727194508	37.0623381	1.14E-09	6.63E-07	DAZ3	389895	-1.149859138	1.800679617	19.02600944	1.28949E-05	0.000446811	LOC389895
106865373	-3.259974622	0.359436063	25.1931396	5.19E-07	0.00017631	WRB-SH3BGR	255313	-2.147743094	-0.578383909	15.96690402	6.44596E-05	0.001976493	CT47A11
728689	-3.629500423	0.492423409	40.22955292	2.26E-10	1.44E-07	EIF3CL	190	-1.029100234	2.086876292	19.11430816	1.23118E-05	0.00042887	NR0B1
643909	-4.424654449	1.379045311	30.8739265	2.75E-08	1.39E-05	SPDYE9P	474343	-1.002517224	2.060927586	18.62665346	1.58982E-05	0.000536728	SPIN2B
8363	-4.427593796	-0.792222864	13.85978925	0.000196968	0.028346139	H4C11	219431	1.010461224	1.264895401	10.40925393	0.001253854	0.02949998	OR4S2
692094	-4.648999871	-0.665096702	17.30859439	3.18E-05	0.006809446	MSMP	203562	-1.069759111	1.303057235	13.28158446	0.000268026	0.007278785	TMEM31

102723737	-4.871219232	-0.155926384	21.00651639	4.58E-06	0.001219525	CT45A8
100287478	-5.33252213	3.137622243	266.8209711	5.59E-60	5.52E-56	USP17L21
728379	-7.434326	3.246330348	118.3463545	1.46E-27	2.39E-24	USP17L26

23676	-1.092266293	1.4363649	14.61085341	0.000132151	0.003776078	SMPX
2833	-1.057441261	2.381162606	24.93314128	5.93533E-07	2.61207E-05	CXCR3
5009	-1.039291752	2.256162418	22.67246141	1.92101E-06	7.66695E-05	OTC
728395	10.3646635	3.758932285	800.6237093	3.95E-176	3.89E-172	TSPY4
378950	10.12375971	3.122862359	657.7814178	4.54E-145	2.98E-141	RBMY1E
378951	9.554577205	3.434136973	570.4379744	4.51E-126	1.48E-122	RBMY1J
9085	8.268130416	0.814179515	175.9534307	3.71E-40	2.52E-37	CDY1
64591	8.178898476	2.107517649	248.8647658	4.59E-56	5.32E-53	TSPY2
9426	8.019450095	1.689395465	216.1046317	6.40E-49	5.73E-46	CDY2A
100289087	7.898338411	2.125734744	226.1540036	4.11E-51	4.05E-48	TSPY10
159163	7.856729574	4.113268354	848.6677868	1.42E-186	2.79E-182	RBMY1F
728137	7.8425186	2.0679	209.47	1.79E-4	1.54E	TSPY3

	11	143	07189	7	-44	
	7.8044946	1.4723	183.84	7.02E-4	5.32E	
86614	07	80279	43899	2	-39	HSFY1
	7.6450143	1.0763	148.74	3.27E-3	2.08E	
9082	81	31231	0643	4	-31	XKRY
	7.6450143	1.0763	148.74	3.27E-3	2.08E	
353515	81	31231	0643	4	-31	XKRY2
	7.6217388	3.4730	626.14	3.45E-1	1.70E	
8287	7	88175	47066	38	-134	USP9Y
	7.5224364	1.8139	236.11	2.77E-5	3.03E	
1617	21	25803	25435	3	-50	DAZ1
	7.3662047	2.9373	446.85	3.49E-9	7.64E	
7258	23	21567	5007	9	-96	TSPY1
	7.1543459	2.6907	414.71	3.45E-9	6.19E	
7544	93	09067	41687	2	-89	ZFY
	7.1464248	0.7270	100.26	1.33E-2	7.49E	
57054	07	09648	98498	3	-21	DAZ3
	7.0139033	0.3644	86.929	1.12E-2	5.41E	
203611	08	19361	93389	0	-18	CDY2B
	6.9820243	1.6925	199.44	2.76E-4	2.18E	
57055	85	21951	64097	5	-42	DAZ2
	6.7334431	2.7340	352.33	1.32E-7	2.00E	
57135	98	29034	01369	8	-75	DAZ4
	6.5870572	3.7586	612.05	4.00E-1	1.58E	
9081	25	40694	32516	35	-131	PRY
378949	6.4881407	2.2621	219.84	9.79E-5	9.19E	RBMY1D

	12	73286	17291	0	-47	
	6.4414040	1.9247	235.36	4.02E-5	4.18E	
159119	11	98224	84244	3	-50	HSFY2
	6.3049866	2.9922	317.41	5.30E-7	6.96E	
253175	76	52083	4354	1	-68	CDY1B
	6.2061078	0.8305	107.27	3.87E-2	2.25E	
9086	99	23824	52178	5	-22	EIF1AY
	5.9900606	0.0778	59.640	1.14E-1	4.08E	
6736	01	90516	88265	4	-12	SRY
	5.9118116	0.4682	65.373	6.20E-1	2.49E	
442867	9	35218	27954	6	-13	BPY2B
	5.9118116	0.4682	65.373	6.20E-1	2.49E	
442868	9	35218	27954	6	-13	BPY2C
	5.9118116	0.4682	65.373	6.20E-1	2.49E	
9083	9	35218	27954	6	-13	BPY2
	5.5348575	1.1052	131.09	2.37E-3	1.46E	
9087	28	33002	10243	0	-27	TMSB4Y
	5.4614697	0.3020	70.777	4.00E-1	1.88E	
5940	45	30773	35399	7	-14	RBMY1A1
	5.2537626	1.3772	111.51	4.57E-2	2.73E	
378948	6	76741	36127	6	-23	RBMY1B
		-0.116				
	5.2362127	94707	44.952	2.02E-1	2.75E	
442862	65	9	03717	1	-09	PRY2
1002874	4.6493205	3.1376	460.47	3.79E-1	9.35E	
78	46	98402	28272	02	-99	USP17L21

140032	4.110289811	0.225896638	47.51352459	5.46E-12	9.45E-10	RPS4Y2
8284	4.031976608	3.299843424	381.0972642	7.18E-85	1.18E-81	KDM5D
266	3.9741816	-0.227650192	34.08896729	5.26E-09	3.58E-07	AMELY
90655	3.823897823	0.35948722	58.87733743	1.68E-14	5.80E-12	TGIF2LY
728369	3.669783361	3.62858538	319.5694083	1.80E-71	2.53E-68	USP17L24
22829	3.607951039	4.793950763	510.5510182	4.81E-113	1.36E-109	NLGN4Y
90665	3.436430925	3.243837283	284.5633006	7.61E-64	9.37E-61	TBL1Y
9084	3.195867624	-0.477625752	25.56746465	4.27E-07	1.93E-05	VCY
6192	3.142996289	1.034067616	70.52832217	4.54E-17	2.03E-14	RPS4Y1
100287327	3.141595906	4.417932179	425.5997085	1.47E-94	2.91E-91	USP17L17
8653	3.058542887	2.923297692	201.061884	1.22E-45	1.01E-42	DDX3Y
728403	2.742610447	1.278653216	27.0715673	1.96E-07	9.43E-06	TSPY8

	2.5705165	3.4240	178.69	9.33E-4	6.57E	
728929	32	47658	74819	1	-38	ELOA3B
	2.5089186	2.2298	88.998	3.95E-2	2.00E	
7356	61	35271	2311	1	-18	SCGB1A1
	1.5741775	3.6847	52.482	4.34E-1	1.06E	
728373	27	76084	46419	3	-10	USP17L25
	1.5371261	1.9141	31.902	1.62E-0	9.96E	
120146	56	01414	65565	8	-07	TRIM64
	1.4818899	4.2996	93.698	3.68E-2	1.96E	
162699	61	10132	09363	2	-19	ELOA3
	1.2011966	4.0154	70.530	4.53E-1	2.03E	
728405	82	27338	20063	7	-14	USP17L29
	1.1552709	4.2237	65.274	6.52E-1	2.57E	
6607	98	43031	40262	6	-13	SMN2
1027248	1.1440533	4.0629	53.294	2.87E-1	7.39E	
62	31	52426	17147	3	-11	TBC1D3I
	1.1165822	3.2951	40.303	2.17E-1	2.12E	
728386	64	58265	56881	0	-08	USP17L5
	1.1155037	2.5099	25.387	4.69E-0	2.10E	
6590	43	44734	18228	7	-05	SLPI
	1.1106801	2.6557	27.751	1.38E-0	6.83E	
6205	2	50936	41486	7	-06	RPS11
	1.0749513	2.3974	25.544	4.32E-0	1.95E	
10399	25	74039	47122	7	-05	RACK1
	1.0688057	3.6993	49.310	2.18E-1	4.49E	
6606	05	86609	7127	2	-10	SMN1

301	1.012079347	3.420165464	34.21098345	4.94E-09	3.41E-07	ANXA1
9248	-1.001304798	3.401447001	39.35838428	3.52735E-10	3.26503E-08	GPR50
84889	-1.002574494	2.571259938	25.87594261	3.64078E-07	1.66041E-05	SLC7A3
64061	-1.002759422	3.783301433	44.24172374	2.90229E-11	3.73997E-09	TSPYL2
57526	-1.003996147	4.817732959	53.49626955	2.59084E-13	6.90285E-11	PCDH19
84968	-1.004879879	3.005226845	31.85443285	1.6617E-08	1.01133E-06	PNMA6A
8233	-1.006402779	3.414329462	38.40775498	5.74038E-10	5.00785E-08	ZRSR2
4068	-1.006455605	3.132759306	35.93264842	2.04257E-09	1.53123E-07	SH2D1A
347442	-1.007303957	3.762106676	43.68970638	3.8479E-11	4.7714E-09	DCAF8L2
1027244	-1.0073634	3.0055	35.689	2.3142	1.717	GAGE10

73	05	12861	37379	3E-09	69E-07	
158586	-1.008454067	4.019612592	46.60298583	8.69277E-12	1.42822E-09	ZXDB
8471	-1.009159425	4.570932914	53.61422217	2.43987E-13	6.68117E-11	IRS4
6567	-1.010681623	3.775559567	45.65255402	1.41202E-11	1.98853E-09	SLC16A2
170261	-1.012711444	2.716584468	30.11985965	4.06151E-08	2.29446E-06	ZCCHC12
644596	-1.014482425	2.889424188	31.15955114	2.37667E-08	1.40716E-06	SMIM10L2B
100329135	-1.014584621	3.345688393	38.43526642	5.66003E-10	4.95969E-08	TRPC5OS
158835	-1.014920823	2.722398906	30.80448681	2.85377E-08	1.6401E-06	AWAT2
83550	-1.015989076	4.793524429	56.40637075	5.89391E-14	1.90499E-11	GPR101
2334	-1.0170808	5.4954	48.358	3.5494	6.707	AFF2

	37	78221	83798	1E-12	35E-10	
7403	-1.019153449	4.022797722	46.94705548	7.29305E-12	1.23957E-09	KDM6A
347516	-1.022569283	2.643999093	29.26435362	6.31467E-08	3.39237E-06	DGAT2L6
64860	-1.022704957	3.313107744	39.82601524	2.77623E-10	2.61895E-08	ARMCX5
55086	-1.022706118	3.482492392	42.16070312	8.40725E-11	9.54462E-09	RADX
1260	-1.024776642	3.340187817	40.54276357	1.9236E-10	1.89628E-08	CNGA2
2157	-1.025300363	4.924459619	53.64545716	2.40138E-13	6.6684E-11	F8
3358	-1.028681037	3.928964363	48.64184703	3.07243E-12	5.99762E-10	HTR2C
10479	-1.02919428	4.017611267	49.68165281	1.80829E-12	3.79279E-10	SLC9A6
8242	-1.0297227	4.1237	49.831	1.6753	3.590	KDM5C

	28	65302	46554	6E-12	36E-10	
4129	-1.029896581	3.056821146	37.30275354	1.01142E-09	8.20621E-08	MAOB
139170	-1.032807703	3.133959143	36.97636703	1.1957E-09	9.46762E-08	DCAF12L1
139221	-1.034361236	3.891505251	48.26952801	3.7148E-12	6.87848E-10	PWWP3B
4168	-1.034744043	3.638486403	44.35884715	2.73374E-11	3.54595E-09	MCF2
6853	-1.035494865	2.946590375	35.67936987	2.32614E-09	1.71769E-07	SYN1
695	-1.036558586	3.11454937	38.00002231	7.07438E-10	6.01201E-08	BTK
154796	-1.037761798	4.800266562	55.61819183	8.80054E-14	2.71112E-11	AMOT
170062	-1.038270145	2.693694103	28.64503879	8.69364E-08	4.52253E-06	FAM47B

55869	-1.0406751 24	3.7031 72998	46.327 6352	1.0004 3E-11	1.577 96E-0 9	HDAC8
56062	-1.0417564 38	4.1248 99771	51.621 30865	6.7307 1E-13	1.598 83E-1 0	KLHL4
54967	-1.0420228 51	3.5037 01094	44.385 93127	2.6961 8E-11	3.520 39E-0 9	CT55
492	-1.0434400 35	4.5507 26654	54.494 05177	1.5591 9E-13	4.520 74E-1 1	ATP2B3
80258	-1.0435284 99	4.0213 28308	51.410 3158	7.4943 7E-13	1.759 04E-1 0	EFHC2
9843	-1.0453412 5	4.4006 84537	55.578 13038	8.9817 3E-14	2.724 37E-1 1	HEPH
55922	-1.0461809 06	3.6417 38324	46.132 90325	1.1049 7E-11	1.686 55E-0 9	NKRF
90161	-1.0505685 67	4.4842 34907	59.314 02024	1.3441 5E-14	4.732 38E-1 2	HS6ST2
6247	-1.0514776 42	3.0741 80771	37.732 61893	8.1136 4E-10	6.778 33E-0	RS1

					8	
57393	-1.0518112	2.725019376	31.02757559	2.54388E-08	1.48373E-06	CLTRN
8852	-1.052170952	3.329439473	44.1827489	2.99106E-11	3.82933E-09	AKAP4
7101	-1.052392193	5.264122681	45.27718964	1.7103E-11	2.35805E-09	NR2E1
203522	-1.053880647	4.194909023	54.09180379	1.91338E-13	5.46727E-11	INTS6L
84295	-1.061691627	3.9420524	48.34634992	3.57208E-12	6.70735E-10	PHF6
6839	-1.061995625	3.071075267	38.08594477	6.7696E-10	5.7779E-08	SUV39H1
3897	-1.062294353	3.442801539	44.76935025	2.21665E-11	2.99339E-09	L1CAM
10800	-1.062352451	3.169722039	38.9041429	4.45135E-10	4.02582E-08	CYSLTR1
203430	-1.062745028	3.02230176	37.63512062	8.52948E-10	7.03629E-0	RTL3

					8	
5456	-1.066810709	3.293606372	41.6321818	1.10165E-10	1.18808E-08	POU3F4
139067	-1.069549262	2.515749251	29.09331185	6.89747E-08	3.68538E-06	SPANXN3
347365	-1.071332982	3.943578685	52.74733752	3.79337E-13	9.4671E-11	ITIH6
9104	-1.077087429	2.755365519	33.07830204	8.85211E-09	5.74106E-07	RGN
5199	-1.07877813	3.385439594	45.78144902	1.3221E-11	1.93085E-09	CFP
51209	-1.079156286	3.847912077	52.81695998	3.66126E-13	9.25454E-11	RAB9B
4100	-1.079657662	2.343204292	25.24960614	5.03699E-07	2.24174E-05	MAGEA1
100507170	-1.080856453	3.711004288	46.37627946	9.75898E-12	1.55168E-09	CT47A12
727866	-1.082164832	4.157043963	53.49909501	2.58712E-13	6.90285E-1	FAM156B

					1	
100130086	-1.086425843	2.779415401	31.02204259	2.55115E-08	1.48373E-06	HSFX2
412	-1.094861737	4.597347379	62.93798811	2.13318E-15	8.08803E-13	STS
10544	-1.100054032	4.418224371	63.87935833	1.32276E-15	5.11365E-13	PROCR
6901	-1.105846641	2.679907415	33.36932019	7.62169E-09	5.02573E-07	TAZ
1536	-1.111174607	3.819129542	55.34988394	1.00876E-13	2.96846E-11	CYBB
4111	-1.113742694	2.481736128	29.54826367	5.45415E-08	2.98706E-06	MAGEA12
2239	-1.116328522	3.886678324	55.42575558	9.70564E-14	2.89934E-11	GPC4
7789	-1.116444939	3.940033974	55.88718902	7.67511E-14	2.40194E-11	ZXDA
286514	-1.1182949	2.4259	28.497	9.3823	4.804	MAGEB18

	07	26237	42016	4E-08	73E-06	
158506	-1.12748628	2.58623527	33.3836313	7.56581E-09	5.00562E-07	CBLL2
79589	-1.132000927	3.063384471	43.31633128	4.65681E-11	5.70271E-09	RNF128
55613	-1.135643633	3.114166069	45.35163237	1.64651E-11	2.28609E-09	MTMR8
404281	-1.140420967	3.302452592	47.5354434	5.40184E-12	9.42501E-10	YY2
81557	-1.142187421	2.956322074	42.80947675	6.03391E-11	7.16655E-09	MAGED4B
56001	-1.145646961	2.484608296	31.92526325	1.6022E-08	9.87154E-07	NXF2
158724	-1.146374614	2.918119878	41.75264037	1.03582E-10	1.14091E-08	FAM47A
645974	-1.14977051	3.014627529	43.07160575	5.27726E-11	6.42262E-09	PABPC1L2B

5354	-1.151287061	3.391782559	50.92570298	9.59291E-13	2.19923E-10	PLP1
6756	-1.162304462	2.476145141	33.55943291	6.91194E-09	4.60391E-07	SSX1
116442	-1.164544769	3.387551587	50.25558393	1.3497E-12	2.98879E-10	RAB39B
55190	-1.165395825	2.938913084	42.06981898	8.8072E-11	9.81033E-09	NUDT11
10742	-1.167507895	2.985117341	44.709569	2.28538E-11	3.04449E-09	RAI2
8266	-1.181066338	2.521052139	33.61348072	6.72252E-09	4.49292E-07	UBL4A
5256	-1.182428162	3.714964583	56.99458334	4.37014E-14	1.46037E-11	PHKA2
1736	-1.187136471	3.222240869	50.29159932	1.32516E-12	2.96895E-10	DKC1
29934	-1.187884852	3.100595791	48.54216339	3.23264E-12	6.24849E-1	SNX12

					0	
107985657	-1.195752409	2.775362891	40.95304562	1.55931E-10	1.59292E-08	LOC107985657
102723680	-1.203056283	3.22738567	49.92769827	1.59517E-12	3.45608E-10	CT45A9
401613	-1.20783864	4.040840033	67.43206473	2.18073E-16	9.55452E-14	SERTM2
56548	-1.210182947	2.228961749	28.0065413	1.20906E-07	6.09663E-06	CHST7
7102	-1.21229208	2.491692882	35.58522324	2.44132E-09	1.78934E-07	TSPAN7
56157	-1.230699586	2.06684102	27.4746041	1.59171E-07	7.74868E-06	TEX13A
4674	-1.231691595	3.200765418	54.01155713	1.99314E-13	5.61382E-11	NAP1L2
347487	-1.250206901	2.272172338	32.6779536	1.08764E-08	6.91738E-07	CXorf66
11040	-1.2565390	2.5452	41.044	1.4878	1.535	PIM2

	16	70129	72688	5E-10	84E-08	
105373133	-1.270792621	3.002289127	48.48952463	3.32058E-12	6.35617E-10	LOC105373133
51481	-1.27453322	1.800402677	26.00588194	3.40379E-07	1.55706E-05	VCX3A
399939	-1.280496307	2.518617358	42.58512913	6.76719E-11	7.94178E-09	TRIM49D1
441521	-1.284860622	3.607045029	56.86303801	4.67246E-14	1.53537E-11	CT45A5
340578	-1.288530427	2.776006815	46.78592115	7.91802E-12	1.31186E-09	DCAF12L2
680	-1.308174326	3.161958718	61.85948176	3.68862E-15	1.37217E-12	BRS3
728419	-1.312751727	3.696197011	48.13664958	3.97526E-12	7.25706E-10	USP17L30
29935	-1.324418999	2.300485189	36.59457153	1.45436E-09	1.13116E-07	RPA4

729447	-1.347842728	2.592911266	49.11353433	2.41568E-12	4.91005E-10	GAGE2A
392509	-1.358961602	2.824971042	53.37412395	2.75705E-13	7.24772E-11	ARL13A
50814	-1.361253414	2.583532001	48.64452584	3.06824E-12	5.99762E-10	NSDHL
795	-1.363814836	1.840606288	29.39649692	5.89842E-08	3.21252E-06	S100G
548313	-1.371184067	2.842599398	53.28551729	2.88425E-13	7.38518E-11	SSX4B
8263	-1.524733399	2.789339083	65.71859027	5.20129E-16	2.22932E-13	F8A1
728379	-1.571916168	3.246270324	90.34068819	2.00487E-21	1.04021E-18	USP17L26
728075	-1.597118436	2.189255335	25.62941968	4.13684E-07	1.87931E-05	CT47A4
728082	-1.617422771	2.056290665	45.7174961	1.36597E-11	1.9658E-09	CT47A3

100533997	-1.706088086	1.635035435	34.38756511	4.51599E-09	3.12412E-07	MAGEA10-MAGEA5
541466	-1.710266962	2.189808988	50.32287049	1.30421E-12	2.9556E-10	CT45A1
100506888	-1.735739937	4.015280149	98.44214882	3.3466E-23	1.83282E-20	ELOA3D
102723547	-1.774883185	1.297122744	35.2694711	2.87097E-09	2.054E-07	CSAG2
728062	-1.977189468	1.457991609	40.5720995	1.89493E-10	1.88613E-08	CT47A6
728072	-2.092664556	1.680850082	34.13524762	5.14115E-09	3.51955E-07	CT47A5
110599583	-2.308285942	0.649272051	28.91647913	7.55673E-08	4.01586E-06	EEF1AKMT4-ECE2
643909	-2.525618756	1.379307976	42.86546744	5.86366E-11	7.04369E-09	SPDYE9P
653656	-2.798565239	0.235275221	25.43027965	4.58662E-07	2.05522E-05	MBD3L4
246100	-2.9891428	0.6057	43.388	4.4887	5.531	CTAG1A

	65	65832	23913	8E-11	3E-09	
100133053	-3.302189131	-0.082118015	46.79003583	7.90141E-12	1.31186E-09	CXorf51B
30014	-3.410882628	0.833449087	57.67074142	3.09878E-14	1.05337E-11	SPANXA1
100996631	-3.411505862	0.538675638	55.96909658	7.36192E-14	2.34109E-11	TCP11X1
728036	-3.44964741	1.43909974	60.60996267	6.9581E-15	2.54048E-12	CT47A10
728096	-4.759012707	1.950720732	178.7753794	8.97035E-41	6.55035E-38	CT47A1
728911	-6.82797168	0.470435114	87.43685465	8.70139E-21	4.28892E-18	CT45A2

Table S9.3. Human genes differentially expressed in severe disease relative to mild disease in OPs

Symptom							Gender						
GeneID	log2FC(Mild/Severe)	logCPM	LR	*p* value	FDR	symbol	GeneID	logFC(Male/Female)	logCPM	LR	*p* value	FDR	symbol
100532726	1.977662169	-0.781844805	20.19133561	7.01E-06	0.000881047	NDUFC2-KCTD14	51480	-1.125286031	0.606363033	31.06243514	2.50E-08	6.41E-07	VCX2
101929627	1.882859061	2.051662685	79.03948756	6.09E-19	6.01E-16	LOC101929627	340542	-1.009777808	1.633955171	51.53684474	7.03E-13	2.22E-11	BEX5
114118903	1.689147311	-0.397026429	21.37391587	3.78E-06	0.000525329	ARHGAP11A-SCG5	347411	-1.004367916	0.923296757	32.21814565	1.38E-08	3.55E-07	MPC1L
101060233	1.451575991	-0.421170453	19.3204124	1.11E-05	0.001338495	OPN1MW3	255313	-1.179394515	-0.039380531	19.85478678	8.36E-06	0.000193594	CT47A11
100129407	1.422627142	-0.076576882	23.28460498	1.40E-06	0.000215476	FAM236A	100132304	-1.070400917	0.974294277	37.24145901	1.04E-09	2.84E-08	FAM236B
100287399	1.414968004	-0.223284029	22.59193051	2.00E-06	0.000297338	POTEB2	8712	-1.018543506	1.020676845	34.75772112	3.73E-09	9.91E-08	PAGE1
100996631	1.3436984	0.972637122	39.41748209	3.42E-10	1.23E-07	TCP11X1	100534592	-2.392272291	-0.490697193	48.18223371	3.88E-12	1.18E-10	URGCP-MRPS24

102724652	1.335679389	0.577335874	34.73278573	3.78E-09	1.08E-06	CRYAA2	90737	-1.015174927	1.475198305	46.48468873	9.23E-12	2.78E-10	PAGE5
728911	1.227469738	0.679134417	27.80552369	1.34E-07	2.76E-05	CT45A2	102723547	-1.061897559	1.157592901	41.53278869	1.16E-10	3.33E-09	CSAG2
102723737	1.117359805	0.349760932	18.1997527	1.99E-05	0.002269698	CT45A8	728458	-1.485365949	-0.535570236	18.09849037	2.10E-05	0.000472181	OPN1MW2
100287441	1.114589605	2.510977226	74.70217965	5.47E-18	5.15E-15	USP17L20	100533997	-1.007679002	1.621465117	49.53693654	1.95E-12	6.05E-11	MAGEA10-MAGEA5
4585	-1.018656198	8.22734002	94.47656364	2.48E-22	3.77E-19	MUC4	795	-1.012851011	1.839048247	59.13819089	1.47E-14	4.96E-13	S100G
3106	-1.022891379	3.280048812	65.40600349	6.10E-16	4.15E-13	HLA-B	8409	-1.079394471	1.308088797	48.42936797	3.42E-12	1.05E-10	UXT
100287205	-1.024296946	3.154435781	67.79600619	1.81E-16	1.33E-13	USP17L12	654364	3.381512565	-0.823556722	48.89713585	2.70E-12	8.29E-11	NME1-NME2
7430	-1.03655821	4.588917261	91.59198774	1.07E-21	1.40E-18	EZR	26548	-1.01420927	1.75348566	57.1997444	3.94E-14	1.30E-12	ITGB1BP2
6205	-1.087139162	3.101520579	60.32840659	8.03E-15	4.53E-12	RPS11	3266	-1.032052433	1.471221253	48.3682253	3.53E-12	1.08E-10	ERAS
643847	-1.100174868	1.217485113	25.41010029	4.63E-07	8.10E-05	PGA4	8363	2.472769301	-0.597775879	37.62394313	8.58E-10	2.35E-08	H4C11
3813	-1.103649925	0.870329734	20.20159108	6.97E-06	0.000881047	KIR3DS1	246100	-1.094728581	0.360787153	25.24059189	5.06E-07	1.24E-05	CTAG1A

1509	-1.104929445	4.21245374	92.85142763	5.64E-22	7.95E-19	CTSD	5355	-1.047576046	1.701163535	57.3237279	3.70E-14	1.22E-12	PLP2
1493	-1.123202355	4.691285161	114.6036571	9.61E-27	2.11E-23	CTLA4	27344	-1.258898386	1.257294017	63.00107778	2.07E-15	7.32E-14	PCSK1N
728929	-1.145393276	3.365576137	82.25164934	1.20E-19	1.39E-16	ELOA3B	89885	-1.017970842	1.731026119	56.2953878	6.24E-14	2.04E-12	FATE1
8000	-1.168509652	3.247695724	73.89518142	8.24E-18	6.78E-15	PSCA	100272147	-1.100688785	1.279153452	49.03717832	2.51E-12	7.75E-11	CMC4
4582	-1.182921171	5.120286932	113.4391885	1.73E-26	3.41E-23	MUC1	56849	-1.002363424	1.954892718	62.78766203	2.30E-15	8.15E-14	TCEAL7
105180391	-1.275853005	0.901937009	26.13247715	3.19E-07	5.83E-05	SPDYE15P	100505385	-3.019172638	-0.979844339	38.27493893	6.14E-10	1.70E-08	IQCJ-SCHIP1
100287478	-1.297553282	3.009613706	79.21788458	5.56E-19	5.78E-16	USP17L21	728049	-1.128054154	1.551912378	51.72921469	6.37E-13	2.01E-11	CT47A8
402317	-1.319669721	1.255347911	34.11736889	5.19E-09	1.42E-06	OR2A42	10549	-1.123953339	1.160423001	46.34800861	9.90E-12	2.97E-10	PRDX4
137797	-1.329790168	3.054237548	81.97476487	1.38E-19	1.51E-16	LYPD2	728656	-1.031324848	1.572231811	51.89621205	5.85E-13	1.85E-11	DMRTC1B
100132396	-1.392708594	0.362190196	23.142429	1.50E-06	0.00022844	ZNF705B	102723737	-1.340925519	0.349778621	35.82138614	2.16E-09	5.83E-08	CT45A8
100462983	-1.44989946	6.364103231	121.5088573	2.96E-28	7.30E-25	MTRNR2L3	11230	-1.072586454	1.905928443	69.04827918	9.61E-17	3.63E-15	PRAF2
79144	-1.460607907	2.852076288	91.38557348	1.18E-21	1.46E-18	PPDPF	107984902	1.740344279	0.778008743	56.68768657	5.11E-14	1.68E-12	LOC107984902
8073	-1.47193	6.79767	171.10	4.23E-3	1.39E	PTP4A2	54830	-1.027072	2.1117	72.383	1.77E-1	6.93	NUP62CL

	6937	3396	96785	9	-35			337	65232	71582	7	E-16	
					0.004							0.007	
10192858	-1.55419	-0.0304	16.677	4.43E-0	46107	LOC10192	1005280	-1.607394	-0.871	12.684	0.00036	6055	
9	2008	00259	98111	5	5	8589	17	655	52536	50114	8699	23	SAA2-SAA4
	-1.59775	5.06638	206.69	7.21E-4	3.56E			-1.007684	2.2525	73.880	8.30E-1	3.30	
26168	1918	2184	94866	7	-43	SENP3	139793	308	4303	44111	8	E-16	PAGE3
	-1.60091	3.79607	163.92	1.57E-3	4.42E		1019289	-1.197273	1.6499	71.951	2.21E-1	8.59	
728369	9991	8812	76743	7	-34	USP17L24	17	012	21151	25266	7	E-16	HSFX3
	-1.72570	0.25565	26.874	2.17E-0	4.24E			-1.190076	0.7831	38.297	6.07E-1	1.68	
126961	0833	671	60272	7	-05	H3C14	552900	094	53592	24848	0	E-08	BOLA2
	-1.97085	4.14408	212.97	3.09E-4	2.03E			-1.036134	2.1603	74.603	5.75E-1	2.30	
3934	3857	2321	14996	8	-44	LCN2	23630	823	98384	36289	8	E-16	KCNE5
	-2.05016	3.63089	180.22	4.32E-4	1.71E		1002888	-1.066149	2.0300	73.418	1.05E-1	4.14	
10406	1406	8754	92751	1	-37	WFDC2	14	123	84403	25757	7	E-16	CLDN34
	-2.29364	0.92262	57.037	4.28E-1	2.22E			-1.017941	1.9146	48.285	3.68E-1	1.12	
728689	3176	5139	75508	4	-11	EIF3CL	728042	148	87734	84501	2	E-10	CT47A9
	-2.44869	1.46079	113.13	2.02E-2	3.62E			-1.066929	1.7645	62.608	2.52E-1	8.87	
728072	5106	2078	43407	6	-23	CT47A5	5956	58	13149	72258	5	E-14	OPN1LW
					0.007				-0.922			0.009	
10052676	-2.49984	-0.8812	15.476	8.35E-0	74214	CCDC169-		1.6993467	10194	12.196	0.00047	6853	
1	3699	82059	54134	5	1	SOHLH2	645359	34	2	28931	8847	61	PRAMEF26
	-2.95603	3.58173	403.12	1.15E-8	1.14E			-1.074099	2.0627	74.978	4.76E-1	1.91	
728373	2953	7722	50262	9	-85	USP17L25	389860	227	654	55922	8	E-16	PAGE2B
	-3.09427	1.37921	111.09	5.65E-2	9.30E			-2.003252	0.0991	62.432	2.76E-1	9.65	
9085	5683	8954	09813	6	-23	CDY1	729396	696	46758	6849	5	E-14	GAGE12J
10028732	-3.13783	3.77246	449.42	9.62E-1	1.90E	USP17L17	644538	-1.009978	2.3046	76.629	2.06E-1	8.45	SMIM10

7	5874	6468	52884	00	-95			024	95074	06106	8	E-17	
10106030	-3.29931	-0.5562	29.016	7.18E-0	1.59E	HNRNPCL		-1.123986	1.0668	42.486	7.12E-1	2.06	
1	644	66679	29192	8	-05	4	474382	099	8248	22556	1	E-09	H2AB1
	-3.32519	-0.5977	27.020	2.01E-0	3.97E			-1.028216	1.1816	40.950	1.56E-1	4.45	
8363	4996	76934	73115	7	-05	H4C11	645188	377	48774	88127	0	E-09	LOC645188
	-4.81545	-0.0394	74.402	6.37E-1	5.72E			-1.067809	1.3725	49.289	2.21E-1	6.84	
255313	0045	88243	36402	8	-15	CT47A11	55859	665	42887	82358	2	E-11	BEX1
												2.20	
								7.4403245	1.3789	622.13	2.56E-1	E-13	
							9085	73	46026	66708	37	4	CDY1
								7.2960254	3.1595	1723.1			
							378950	49	45273	24349	0	0	RBMY1E
								6.7491251	3.7043	2104.6			
							728395	4	74765	40641	0	0	TSPY4
												5.83	
								6.7085547	1.7566	749.43	5.31E-1	E-16	
							64591	88	562	88501	65	2	TSPY2
												2.51	
								6.5853247	1.9852	806.42	2.16E-1	E-17	
							9426	39	18813	92246	77	4	CDY2A
								6.4240502	4.0861	2654.6			
							159163	99	59726	35902	0	0	RBMY1F
								6.1977787	3.2868	1722.4			
							378951	27	3162	03731	0	0	RBMY1J
								6.0085559	1.8175	692.96	1.01E-1	9.97	
							1617	1	83479	96082	52	E-15	DAZ1

					0	
8287	5.914092123	3.360669355	1809.367353	0	0	USP9Y
9081	5.762396881	3.655053617	1930.143675	0	0	PRY
100289087	5.736751126	2.361342363	1011.595982	5.42E-222	8.91E-219	TSPY10
728137	5.723055371	2.077594173	639.8321154	3.63E-141	3.42E-138	TSPY3
57135	5.680567582	2.441329604	934.4266819	3.22E-205	4.54E-202	DAZ4
57054	5.582209882	0.476130646	229.7121362	6.89E-52	3.40E-49	DAZ3
353515	5.546783276	1.056436413	421.7615283	1.01E-93	7.12E-91	XKRY2
9082	5.546783276	1.056436413	421.7615283	1.01E-93	7.12E-91	XKRY
728403	5.360777068	1.487222745	470.5562426	2.43E-104	1.84E-101	TSPY8
7258	5.355111853	3.091658949	1422.036424	3.41895777436637e-31	8.44E-308	TSPY1

				1		
6736	5.326316796	0.466830893	255.460023	1.68E-57	9.19E-55	SRY
253175	5.286226125	2.971461032	1251.776986	3.41E-274	7.48E-271	CDY1B
159119	5.237426446	1.766142987	630.2920175	4.32E-139	3.87E-136	HSFY2
9086	5.136011592	0.929590538	363.2338529	5.56E-81	3.66E-78	EIF1AY
442862	5.029075864	0.480976808	248.1808687	6.47E-56	3.45E-53	PRY2
86614	4.999759642	1.438377168	517.0682975	1.84E-114	1.45E-111	HSFY1
7544	4.902672824	2.514662476	987.7651942	8.20E-217	1.24E-213	ZFY
57055	4.782698397	2.496393207	909.8985941	6.92E-200	9.10E-197	DAZ2
203611	4.77637293	0.344361998	226.6963762	3.13E-51	1.51E-48	CDY2B
378949	4.762316537	2.555109235	1022.876471	1.91E-224	3.43E-22	RBMY1D

					1	
9087	4.730511263	1.228772513	419.4027691	3.29E-93	2.24E-90	TMSB4Y
442867	4.67660273	0.700449016	262.0381799	6.17E-59	3.48E-56	BPY2B
442868	4.67660273	0.700449016	262.0381799	6.17E-59	3.48E-56	BPY2C
9083	4.67660273	0.700449016	262.0381799	6.17E-59	3.48E-56	BPY2
140032	4.490794784	0.431867931	206.3335849	8.67E-47	4.07E-44	RPS4Y2
378948	4.285771123	1.154516165	336.7443912	3.27E-75	2.08E-72	RBMY1B
266	3.997188698	0.098610278	142.9406506	6.06E-33	9.49E-31	AMELY
5940	3.99116642	0.127722279	168.0806287	1.94E-38	6.50E-36	RBMY1A1
8284	3.870367826	3.325497127	1187.878616	2.63E-260	5.19E-257	KDM5D
353513	3.68027556	0.332540456	170.5259299	5.68E-39	2.16E-36	VCY1B
90655	3.630090609	0.523808049	193.4821831	5.52E-44	2.48E-41	TGIF2LY
22829	3.38201299	4.668779667	1512.547435	0	0	NLGN4Y

90665	3.153789585	3.236741751	875.8263187	1.77E-192	2.18E-189	TBL1Y
8653	3.105972636	2.798508773	704.7855276	2.72E-155	2.83E-152	DDX3Y
6192	3.035641165	1.102056476	234.7863786	5.39E-53	2.73E-50	RPS4Y1
100287478	2.533618128	3.009630131	526.6254893	1.53E-116	1.26E-113	USP17L21
100287441	1.496175358	2.511148434	154.3909605	1.90E-35	4.42E-33	USP17L20
728379	1.37774766	3.960767559	236.5300094	2.25E-53	1.17E-50	USP17L26
100287327	1.185625173	3.772377393	167.6247894	2.44E-38	8.04E-36	USP17L17
728929	1.081991111	3.365601765	122.3911253	1.90E-28	1.71E-26	ELOA3B
100130302	-1.000113581	3.526709966	133.0415816	8.85E-31	1.05E-28	SUPT20HL1
2556	-1.001211864	3.667935399	140.9024608	1.69E-32	2.47E-30	GABRA3
63035	-1.001868929	4.785014653	175.0060789	5.97E-40	2.45E-37	BCORL1
1097291	-1.001872	3.2353	119.55	7.91E-2	6.73	FAM236C

26	965	88667	69744	8	E-26	
	-1.001966	3.1228	117.26	2.52E-2	2.01	
6853	044	44279	11195	7	E-25	SYN1
	-1.002635	3.6252	138.16	6.71E-3	8.95	
114824	583	47827	47442	2	E-30	PNMA5
	-1.002794	3.9348	151.63	7.62E-3	1.62	
778	297	2564	32849	5	E-32	CACNA1F
	-1.003528	4.6207	175.64	4.32E-4	1.81	
1288	216	8957	95642	0	E-37	COL4A6
	-1.004819	4.4171	171.07	4.32E-3	1.67	
9737	084	81251	01355	9	E-36	GPRASP1
	-1.007475	2.9585	109.29	1.40E-2	9.68	
9104	559	91518	63632	5	E-24	RGN
	-1.007827	3.1982	120.19	5.72E-2	4.93	
84968	212	14965	99733	8	E-26	PNMA6A
	-1.010423	3.0531	114.77	8.84E-2	6.76	
50943	913	373	05284	7	E-25	FOXP3
	-1.011605	3.9639	157.82	3.38E-3	8.67	
10013	964	77797	48104	6	E-34	HDAC6
1001320	-1.013214	3.8198	151.16	9.65E-3	2.03	
15	259	88545	47274	5	E-32	TEX13D
	-1.014452	3.5084	136.34	1.67E-3	2.14	
8852	073	59657	95191	1	E-29	AKAP4
1053732	-1.014758	3.4636	133.66	6.48E-3	7.85	
97	488	03639	04604	1	E-29	ERVFC1
1003291	-1.014913	3.3170	127.02	1.84E-2	1.80	TRPC5OS

35	822	426	20055	9	E-27	
	-1.016509	2.4680	89.205	3.56E-2	1.72	
10214	919	68299	49619	1	E-19	SSX3
	-1.019419	2.6141	98.027	4.13E-2	2.27	
29071	247	54583	14098	3	E-21	C1GALT1C1
	-1.019662	3.0350	117.57	2.15E-2	1.74	
8226	343	2704	17716	7	E-25	PUDP
	-1.019984	3.8594	159.14	1.74E-3	4.70	
55026	568	39477	66414	6	E-34	TMEM255A
	-1.020335	3.1859	123.54	1.06E-2	9.72	
4674	662	66626	61014	8	E-27	NAP1L2
	-1.023021	3.5412	137.38	9.96E-3	1.30	
8862	98	7479	00392	2	E-29	APLN
	-1.023387	3.2274	124.39	6.92E-2	6.44	
8273	561	52556	13191	9	E-27	SLC10A3
	-1.024484	3.8748	158.67	2.21E-3	5.80	
81887	982	96635	35391	6	E-34	LAS1L
	-1.026847	2.2485	78.873	6.62E-1	2.84	
4105	906	70992	8273	9	E-17	MAGEA6
	-1.027749	2.4894	92.257	7.61E-2	3.79	
8406	342	90352	08997	2	E-20	SRPX
	-1.028841	2.7757	107.71	3.10E-2	2.08	
5931	193	43001	66419	5	E-23	RBBP7
	-1.033555	3.4872	141.24	1.42E-3	2.11	
114928	397	51666	78785	2	E-30	GPRASP2
1053733	-1.036097	4.0466	168.71	1.41E-3	4.93	LOC1053733

81	879	72272	77031	8	E-36	81
	-1.036762	3.5494	143.41	4.77E-3	7.72	
827	165	38035	50504	3	E-31	CAPN6
	-1.044821	3.1843	130.87	2.63E-3	2.95	
369	346	20767	72233	0	E-28	ARAF
	-1.045766	2.2240	77.509	1.32E-1	5.54	
190	12	53923	84405	8	E-17	NR0B1
	-1.046147	3.2274	132.48	1.17E-3	1.37	
6839	672	84889	00398	0	E-28	SUV39H1
	-1.046415	2.9814	119.74	7.21E-2	6.16	
340578	656	16513	06423	8	E-26	DCAF12L2
	-1.047350	3.1697	130.93	2.56E-3	2.88	
203430	535	26847	76447	0	E-28	RTL3
	-1.048545	2.7602	110.35	8.18E-2	5.83	
392517	993	52149	76589	6	E-24	NCBP2L
	-1.055860	2.4326	90.423	1.92E-2	9.37	
139604	789	99006	09069	1	E-20	MAGEB16
	-1.056145	3.9554	169.61	8.99E-3	3.29	
10046	523	97048	2601	9	E-36	MAMLD1
	-1.057988	2.5272	99.938	1.57E-2	9.00	
24140	954	43425	34887	3	E-22	FTSJ1
1001295	-1.059734	4.0023	179.29	6.91E-4	2.96	
15	968	86288	51576	1	E-38	ETDB
	-1.067305	2.4056	92.146	8.05E-2	4.00	
139741	03	18957	54039	2	E-20	ACTRT1
254158	-1.068426	2.2996	86.028	1.77E-2	8.20	CXorf58

	688	34562	46361	0	E-19	
	-1.069101	3.1981	135.56	2.49E-3	3.13	
6247	847	42339	09278	1	E-29	RS1
1027236	-1.077103	2.0838	77.631	1.24E-1	5.25	
31	218	10468	35002	8	E-17	CT45A10
	-1.080222	2.1059	79.982	3.78E-1	1.64	
8544	57	41012	46335	9	E-17	PIR
	-1.098258	3.2292	146.12	1.22E-3	2.24	
695	705	72941	86975	3	E-31	BTK
	-1.099307	2.2416	86.100	1.71E-2	7.92	
728239	453	84417	97807	0	E-19	MAGED4
1005071	-1.106566	3.5953	162.93	2.58E-3	7.84	
70	702	98784	67708	7	E-35	CT47A12
1009966	-1.123701	3.5186	168.51	1.56E-3	5.33	
48	159	09439	10023	8	E-36	TCP11X2
	-1.136459	2.5013	110.32	8.30E-2	5.89	
541466	307	25261	88341	6	E-24	CT45A1
	-1.149813	2.8006	134.12	5.12E-3	6.24	
548313	811	336	84477	1	E-29	SSX4B
1027236	-1.159101	3.2540	155.04	1.37E-3	3.37	
80	002	77947	78219	5	E-33	CT45A9
	-1.167470	3.0835	150.31	1.48E-3	3.05	
4101	7	10978	15371	4	E-32	MAGEA2
1001323	-1.206796	2.3233	117.98	1.75E-2	1.43	
99	401	56017	67454	7	E-25	GAGE12D
728343	-1.208137	2.5774	111.91	3.72E-2	2.75	NXF2B

	851	17176	98196	6	E-24	
	-1.213418	2.3909	115.77	5.33E-2	4.16	
28952	277	75805	3793	7	E-25	CCDC22
	-1.222241	2.5615	137.76	8.22E-3	1.09	
727837	512	80627	18973	2	E-29	SSX2B
1000085	-1.230854	2.7008	145.61	1.57E-3	2.82	
86	448	74314	71621	3	E-31	GAGE12F
	-1.263257	2.4530	131.63	1.80E-3	2.05	
4108	58	04739	3762	0	E-28	MAGEA9
	-1.284167	2.0674	92.777	5.85E-2	2.95	
728096	732	44077	76791	2	E-20	CT47A1
	-1.321667	2.9516	189.24	4.65E-4	2.04	
8263	311	13417	50165	3	E-40	F8A1
1005061	-1.662126	2.3147	205.99	1.03E-4	4.71	
64	362	76827	76468	6	E-44	HSFX1
1019296	-1.695474	2.0522	86.168	1.65E-2	7.68	LOC1019296
27	883	36528	11818	0	E-19	27
	-1.730435	0.8958	90.627	1.73E-2	8.49	
541465	165	44472	73477	1	E-20	CT45A6
	-1.775691	1.5887	130.30	3.51E-3	3.89	
728036	969	28583	99085	0	E-28	CT47A10
	-2.215769	0.6552	115.57	5.88E-2	4.53	
30014	192	68622	9074	7	E-25	SPANXA1
1001330	-2.412236	0.1880	90.134	2.23E-2	1.08	
53	628	07087	03367	1	E-19	CXorf51B
728082	-2.462558	1.8350	277.11	3.19E-6	1.97	CT47A3

								771	06561	76062	2	E-59	

Table S10.1. Human genes differing in expression between severe and mild disease, or between males and females in Faeces

Symptom							Gender						
GeneID	log2FC(Severe/Moderate)	logCPM	LR	*p* value	FDR	symbol	GeneID	log2FC(Male/Female)	logCPM	LR	*p* value	FDR	symbol
Positive													
728689	2.592879531	0.674252621	38.53150253	5.39E-10	3.93E-07	EIF3CL	728689	1.678879173	0.67416504	21.53763527	3.47E-06	0.00010887	EIF3CL
100463289	2.349447744	8.398867601	134.5723802	4.10E-31	4.03E-27	MTRNR2L5	100463289	1.301190198	8.398851346	81.3494523	1.89E-19	6.21E-17	MTRNR2L5
203611	2.252869487	0.207369081	19.24570074	1.15E-05	0.00263196	CDY2B	203611	2.867335474	0.207312218	35.0623849	3.19E-09	1.46E-07	CDY2B
9085	2.096476183	0.918420915	13.29461461	0.00026617	0.037638574	CDY1	9085	6.397595319	0.918489458	104.9294938	1.27E-24	5.67E-22	CDY1
100289087	2.090350377	1.905086365	28.48453306	9.44E-08	4.23E-05	TSPY10	100289087	7.933974175	1.905009822	306.4526894	1.29E-68	1.59E-65	TSPY10
100293534	1.630815792	3.181002953	67.30953791	2.32E-16	3.81E-13	C4B_2	100293534	1.073002219	3.180977016	47.08015781	6.81E-12	4.99E-10	C4B_2
1004634	1.445636	9.91946	51.638	6.67E-1	8.77E	MTRNR2L	1004634	1.8972971	9.9194	151.74	7.20E-3	5.26	MTRNR2L6

82	855		23827	3	-10	6	82	89	54889	58218	5	E-32	
1617	1.218911<br>501	2.25908<br>8341	15.347<br>28984	8.94E-0<br>5	0.015<br>54057<br>4	DAZ1	1617	3.5073344<br>09	2.2594<br>33251	216.55<br>78489	5.10E-4<br>9	4.37<br>E-46	DAZ1
1004634<br>88	1.193981<br>752	10.6458<br>3246	34.615<br>701	4.02E-0<br>9	2.20E<br>-06	MTRNR2L<br>10	1004634<br>88	1.8583540<br>38	10.645<br>83105	150.75<br>74552	1.18E-3<br>4	8.33<br>E-32	MTRNR2L1<br>0
1004629<br>81	1.014807<br>909	14.2844<br>2467	22.334<br>62113	2.29E-0<br>6	0.000<br>73991<br>1	MTRNR2L<br>2	1004629<br>81	1.8332636<br>67	14.284<br>42463	141.19<br>07234	1.46E-3<br>2	9.60<br>E-30	MTRNR2L2
221786	-1.19520<br>3524	6.16160<br>1734	33.972<br>64743	5.59E-0<br>9	2.98E<br>-06	FAM200A	221786	-1.134623<br>131	6.1616<br>48509	60.670<br>31075	6.75E-1<br>5	9.71<br>E-13	FAM200A
26168	-1.39911<br>1527	7.36238<br>7647	33.007<br>60625	9.18E-0<br>9	4.52E<br>-06	SENP3	26168	-1.123262<br>176	7.3619<br>03973	44.908<br>00377	2.07E-1<br>1	1.32<br>E-09	SENP3
4108	-1.77876<br>1299	2.47150<br>7643	26.350<br>03929	2.85E-0<br>7	0.000<br>11005	MAGEA9	4108	-1.827070<br>684	2.4715<br>61247	97.556<br>65463	5.23E-2<br>3	2.10<br>E-20	MAGEA9
147199	-2.03868<br>8963	1.92205<br>0162	23.697<br>27148	1.13E-0<br>6	0.000<br>37655<br>9	SCGB1C1	147199	-1.062882<br>905	1.9221<br>62443	25.236<br>78632	5.07E-0<br>7	1.77<br>E-05	SCGB1C1
414060	-2.48583<br>4094	1.80704<br>0814	30.252<br>21276	3.79E-0<br>8	1.78E<br>-05	TBC1D3C	414060	-1.492094<br>855	1.8070<br>58284	45.207<br>28409	1.77E-1<br>1	1.15<br>E-09	TBC1D3C
728405	-3.19976<br>0199	2.92826<br>7127	27.113<br>39613	1.92E-0<br>7	7.88E<br>-05	USP17L29	728405	-1.643587<br>033	2.9289<br>8869	77.249<br>77677	1.51E-1<br>8	4.43<br>E-16	USP17L29
284428	-4.22406<br>9183	0.76251<br>8334	27.873<br>5692	1.30E-0<br>7	5.55E<br>-05	MBD3L5	284428	-1.048809<br>415	0.7625<br>16784	11.043<br>62337	0.00088<br>9928	0.020<br>2738<br>88	MBD3L5
1001294	-4.23553	0.41201	12.990	0.0003	0.042	FAM236A	1001294	-1.750257	0.4120	21.352	3.82E-0	0.000	FAM236A

07	3824	4878	00839	13158	854743		07	761	55322	18865	6	119545	
105180390	-4.570901385	0.079396735	14.91306014	0.000112581	0.018643017	SPDYE13P	105180390	-2.956194513	0.079460509	39.39900909	3.45E-10	1.84E-08	SPDYE13P
728090	-4.740519262	2.406246603	70.24144726	5.25E-17	9.40E-14	CT47A2	728090	-1.349549254	2.40637097	53.27804716	2.90E-13	2.75E-11	CT47A2
100287441	-5.955340997	2.480439533	36.54553461	1.49E-09	9.48E-07	USP17L20	100287441	-1.046957856	2.480560739	24.34217902	8.07E-07	2.75E-05	USP17L20
Negative													
728419	1.564322033	3.415222685	31.98361708	1.55E-08	7.47E-06	USP17L30	728419	-1.533659345	3.41560296	98.3657709	3.48E-23	1.43E-20	USP17L30
728137	-1.624516231	2.350640594	21.4203136	3.69E-06	0.001084838	TSPY3	728137	3.42104731	2.350401537	224.1072834	1.15E-50	1.08E-47	TSPY3
728712	-3.276689562	0.331771375	26.0655189	3.30E-07	0.000122707	SPANXA2	728712	2.59245709	0.331702178	37.81793659	7.77E-10	3.91E-08	SPANXA2
105180391	-6.10554192	0.78183056	34.85289483	3.56E-09	2.06E-06	SPDYE15P	105180391	3.042695801	0.781810241	65.5784534	5.58E-16	1.05E-13	SPDYE15P
102724127	-8.600429903	2.287544846	129.5902565	5.04E-30	3.31E-26	TP53TG3F	102724127	1.427984261	2.287449855	49.56782811	1.92E-12	1.56E-10	TP53TG3F

Table S10.2. Human genes differing in expression between severe and mild disease, or between males and females in NPs

Symptom							Gender						
GeneID	log2FC(Severe/Moderate)	logCPM	LR	*p* value	FDR	symbol	GeneID	log2FC(Male/Female)	logCPM	LR	*p* value	FDR	symbol
Positive													
102724101	3.409239401	1.682985962	132.170397	1.37E-30	2.46E-27	TP53TG3E	102724101	1.181033418	1.68283341	14.62183552	0.000131383	0.003759586	TP53TG3E
728373	2.224125563	3.684657515	201.5811913	9.44E-46	3.72E-42	USP17L25	728373	1.574177527	3.684776084	52.48246419	4.34E-13	1.06E-10	USP17L25
100289087	1.686255215	2.125891291	31.51780435	1.98E-08	1.11E-05	TSPY10	100289087	7.898338411	2.125734744	226.1540036	4.11E-51	4.05E-48	TSPY10
728369	1.597117792	3.628514672	60.23645652	8.41E-15	8.73E-12	USP17L24	728369	3.669783361	3.62858538	319.5694083	1.80E-71	2.53E-68	USP17L24
57135	1.561208539	2.734137985	53.44948184	2.65E-13	2.18E-10	DAZ4	57135	6.733443198	2.734029034	352.3301369	1.32E-78	2.00E-75	DAZ4
9085	1.459351383	0.814352166	15.96033873	6.47E-05	0.011808338	CDY1	9085	8.268130416	0.814179515	175.9534307	3.71E-40	2.52E-37	CDY1
7730	1.220579868	1.522767399	13.24459447	0.000273367	0.036172514	ZNF177	7730	1.091117924	1.52312757	10.45482006	0.001223298	0.02888449	ZNF177
1001300	-1.15826	2.7794	26.138	3.18E-	0.000	HSFX2	1001300	-1.0864258	2.7794	31.022	2.55E-0	1.48E	HSFX2

86	2304	30678	90742	07	120464		86	43	15401	04259	8	-06	
7101	-1.503044673	5.264104717	78.57051954	7.72E-19	9.51E-16	NR2E1	7101	-1.052392193	5.264122681	45.27718964	1.71E-11	2.36E-09	NR2E1
728082	-1.568590962	2.056207457	26.73548151	2.33E-07	9.39E-05	CT47A3	728082	-1.617422771	2.056290665	45.7174961	1.37E-11	1.97E-09	CT47A3
728036	-1.870669377	1.438842214	20.29441836	6.64E-06	0.001656992	CT47A10	728036	-3.44964741	1.43909974	60.60996267	6.96E-15	2.54E-12	CT47A10
728419	-1.972777166	3.696194944	92.10531545	8.22E-22	1.08E-18	USP17L30	728419	-1.312751727	3.696197011	48.13664958	3.98E-12	7.26E-10	USP17L30
110599583	-2.163352004	0.649395032	24.37689175	7.92E-07	0.000256027	EEF1AKMT4-ECE2	110599583	-2.308285942	0.649272051	28.91647913	7.56E-08	4.02E-06	EEF1AKMT4-ECE2
643909	-4.424654449	1.379045311	30.8739265	2.75E-08	1.39E-05	SPDYE9P	643909	-2.525618756	1.379307976	42.86546744	5.86E-11	7.04E-09	SPDYE9P
102723737	-4.871219232	-0.155926384	21.00651639	4.58E-06	0.001219525	CT45A8	102723737	-1.650459207	-0.155961606	13.00702931	0.000310324	0.008358395	CT45A8
728379	-7.434326	3.246330348	118.3463545	1.46E-27	2.39E-24	USP17L26	728379	-1.571916168	3.246270324	90.34068819	2.00E-21	1.04E-18	USP17L26
Negative													
653275	2.227807013	0.102512592	30.17762909	3.94E-08	1.85E-05	CFC1B	653275	-1.404276093	0.102365065	9.379554994	0.002194188	0.048882048	CFC1B
1005280	1.770981	-0.0753	14.486	0.0001	0.022	FAM187A	1005280	-1.6830639	-0.075	14.429	0.00014	0.004	FAM187A

20	217	38684	55403	41164	105604		20	77	500844	91826	5473	126821	
3188	1.358728451	1.735721595	19.9480142	7.96E-06	0.001961164	HNRNPH2	3188	-1.23978746	1.735833388	21.65633153	3.26E-06	0.000124132	HNRNPH2
728137	-1.548839121	2.06808542	30.9406436	2.66E-08	1.38E-05	TSPY3	728137	7.842518611	2.0679143	209.4707189	1.79E-47	1.54E-44	TSPY3
728929	-1.716350656	3.424124923	59.96613962	9.65E-15	9.51E-12	ELOA3B	728929	2.570516532	3.424047658	178.6974819	9.33E-41	6.57E-38	ELOA3B
100529144	-1.934332679	0.02424053	12.61236806	0.000383203	0.047664357	CORO7-PAM16	100529144	1.694778864	0.024076889	13.4292944	0.000247725	0.006774135	CORO7-PAM16
1617	-2.106823241	1.814129975	46.24418125	1.04E-11	7.62E-09	DAZ1	1617	7.522436421	1.813925803	236.1125435	2.77E-53	3.03E-50	DAZ1
728403	-2.116278775	1.278719041	24.1821383	8.76E-07	0.000278699	TSPY8	728403	2.742610447	1.278653216	27.0715673	1.96E-07	9.43E-06	TSPY8
100287327	-2.351678554	4.417893773	144.2633292	3.11E-33	6.14E-30	USP17L17	100287327	3.141595906	4.417932179	425.5997085	1.47E-94	2.91E-91	USP17L17
728405	-2.749968049	4.015390002	182.052874	1.73E-41	4.86E-38	USP17L29	728405	1.201196682	4.015427338	70.53020063	4.53E-17	2.03E-14	USP17L29
353144	-3.004351359	0.070709382	23.55166858	1.22E-06	0.000368853	LCE3C	353144	1.544784321	0.070664643	10.88463696	0.000969652	0.023628558	LCE3C
57054	-3.189427387	0.727194508	37.0623381	1.14E-09	6.63E-07	DAZ3	57054	7.146424807	0.727009648	100.2698498	1.33E-23	7.49E-21	DAZ3

8363	-4.427593796	-0.792222864	13.85978925	0.000196968	0.028346139	H4C11	8363	3.487458974	-0.792106244	12.28204084	0.000457338	0.011974602	H4C11
100287478	-5.33252213	3.137622243	266.8209711	5.59E-60	5.52E-56	USP17L21	100287478	4.649320546	3.137698402	460.4728272	3.79E-102	9.35E-99	USP17L21

Table S10.3. Human genes differing in expression between severe and mild disease, or between males and females in OPs

Symptom							Gender						
GeneID	log2FC(Severe/Moderate)	logCPM	LR	*p* value	FDR	symbol	GeneID	log2FC(Male/Female)	logCPM	LR	*p* value	FDR	symbol
Positive													
8363	3.325194996	-0.597776934	27.02073115	2.01E-07	3.97E-05	H4C11	8363	2.472769301	-0.597775879	37.62394313	8.58E-10	2.35E-08	H4C11
100287327	3.137835874	3.772466468	449.4252884	9.62E-100	1.90E-95	USP17L17	100287327	1.185625173	3.772377393	167.6247894	2.44E-38	8.04E-36	USP17L17
9085	3.094275683	1.379218954	111.0909813	5.65E-26	9.30E-23	CDY1	9085	7.440324573	1.378946026	622.1366708	2.56E-137	2.20E-134	CDY1
100287478	1.297553282	3.009613706	79.21788458	5.56E-19	5.78E-16	USP17L21	100287478	2.533618128	3.009630131	526.6254893	1.53E-116	1.26E-113	USP17L21
728929	1.145393276	3.365576137	82.25164934	1.20E-19	1.39E-16	ELOA3B	728929	1.081991111	3.365601765	122.3911253	1.90E-28	1.71E-26	ELOA3B

102723737	-1.117359805	0.349760932	18.1997527	1.99E-05	0.002269698	CT45A8	102723737	-1.340925519	0.349778621	35.82138614	2.16E-09	5.83E-08	CT45A8
101929627	-1.882859061	2.051662685	79.03948756	6.09E-19	6.01E-16	LOC101929627	101929627	-1.695474883	2.052236528	86.16811818	1.65E-20	7.68E-19	LOC101929627
Negative													
255313	4.815450045	-0.039488243	74.40236402	6.37E-18	5.72E-15	CT47A11	255313	-1.179394515	-0.039380531	19.85478678	8.36E-06	0.000193594	CT47A11
100287441	-1.114589605	2.510977226	74.70217965	5.47E-18	5.15E-15	USP17L20	100287441	1.496175358	2.511148434	154.3909605	1.90E-35	4.42E-33	USP17L20

Table S11.1. Twenty genes differentially expressed between patients with mild or severe disease in Faeces

Gene ID	Relationship	Symbol	Function	Chromosome
NM_001018081.1	negative	KIR2DL5B	killer cell immunoglobulin like receptor%2C two Ig domains and long cytoplasmic tail 5B	19
NM_001198793.1	negative	ARPC4-TTLL3	ARPC4-TTLL3 readthrough	3
NM_001277397.2	negative	RFPL4AL1	ret finger protein like 4A like 1	19
NM_032621.4	negative	BEX2	brain expressed X-linked 2%2C transcript variant 3	X
XM_005277431.4	negative	POLR3GL	RNA polymerase III subunit GL%2C transcript variant X1	1
XM_017023956.1	negative	TP53TG3F	TP53 target 3 family member F%2C transcript variant X2	16
XM_017029724.2	negative	BGN	biglycan%2C transcript variant X1	X
XM_024450242.1	negative	CLEC18C	C-type lectin domain family 18 member C%2C transcript variant X5	16
XM_024453278.1	negative	LOC102724965	uncharacterized LOC102724965%2C transcript variant X2	2
NM_001001722.1	positive	CDY2B	chromodomain Y-linked 2B	Y
NM_001005241.3	positive	OR4N4	olfactory receptor family 4 subfamily N member 4	15
NM_001242823.2	positive	C4B_2	complement component 4B (Chido blood group)%2C copy 2	6
NM_001256686.2	positive	CSNK2A3	casein kinase 2 alpha 3	11
NM_145208.2	positive	MBD3L1	methyl-CpG binding domain protein 3 like 1	19
NM_199250.2	positive	C19orf48	chromosome 19 open reading frame 48%2C transcript variant 2	19
XM_011531383.2	positive	CT47A8	cancer/testis antigen family 47 member A8%2C transcript variant X1	X
XM_011531385.2	positive	CT47A4	cancer/testis antigen family 47 member A4%2C transcript variant X1	X
XM_011541262.1	positive	AMY1B	amylase alpha 1B%2C transcript variant X1	1
XM_017025781.1	positive	MC2R	melanocortin 2 receptor%2C transcript variant X1	18
XM_024449717.1	positive	RPS6KL1	ribosomal protein S6 kinase like 1%2C transcript variant X5	14

Table S11.2. Twenty genes differentially expressed between patients with mild or severe disease in OPs

Gene ID	Relationship	Symbol	Function	Chromosome
NM_001078171.2	negative	RTL8C	retrotransposon Gag like 8C	X
NM_001256852.1	negative	USP17L10	ubiquitin specific peptidase 17 like family member 10	4
NM_001277423.1	negative	TCP11X2	t-complex 11 family%2C X-linked 2	X
NM_001351372.1	negative	NBPF26	NBPF member 26	1
NM_001354422.1	negative	GAGE12G	G antigen 12G%2C transcript variant 2	X
NM_001355277.1	negative	CENPVL1	centromere protein V like 1	X
NM_001355278.1	negative	CENPVL2	centromere protein V like 2	X
NM_003512.4	negative	H2AC6	H2A clustered histone 6	6
NM_003520.4	negative	H2BC15	H2B clustered histone 15	6
NM_018476.4	negative	BEX1	brain expressed X-linked 1	X
NM_175744.5	negative	RHOC	ras homolog family member C%2C transcript variant 1	1
NM_176783.2	negative	PSME1	proteasome activator subunit 1%2C transcript variant 2	14
NM_201222.3	negative	MAGED2	MAGE family member D2%2C transcript variant 3	X
XM_005262410.4	negative	CCDC160	coiled-coil domain containing 160%2C transcript variant X1	X
XM_017029301.1	negative	USP51	ubiquitin specific peptidase 51%2C transcript variant X3	X
XM_017029451.2	negative	YIPF6	Yip1 domain family member 6%2C transcript variant X4	X
XM_017029472.1	negative	HCFC1	host cell factor C1%2C transcript variant X6	X
XM_017029613.1	negative	SSX4B	SSX family member 4B%2C transcript variant X1	X
XM_017029727.2	negative	TSPYL2	TSPY like 2%2C transcript variant X2	X
XM_024452366.1	negative	GLRA2	glycine receptor alpha 2%2C transcript variant X7	X

Table S11.3. Twenty genes differentially expressed between patients with mild or severe disease in NPs

Gene ID	Relationship	Symbol	Function	Chromosome
NM_001362475.2	negative	CDKN1C	cyclin dependent kinase inhibitor 1C%2C transcript variant 5	11
NM_003122.5	negative	SPINK1	serine peptidase inhibitor Kazal type 1%2C transcript variant 2	5
NM_004894.3	negative	ATP5MPL	ATP synthase membrane subunit 6.8PL%2C transcript variant 1	14
NM_005947.3	negative	MT1B	metallothionein 1B	16
NM_006087.4	negative	TUBB4A	tubulin beta 4A class IVa%2C transcript variant 3	19
NM_017807.4	negative	OSGEP	O-sialoglycoprotein endopeptidase	14
NM_021066.3	negative	H2AC14	H2A clustered histone 14	6
NM_024003.3	negative	L1CAM	L1 cell adhesion molecule%2C transcript variant 2	X
NM_024299.4	negative	PPDPF	pancreatic progenitor cell differentiation and proliferation factor%2C transcript variant 1	20
NM_080670.3	negative	SLC35A4	solute carrier family 35 member A4	5
NM_206873.2	negative	PPP1CA	protein phosphatase 1 catalytic subunit alpha%2C transcript variant 2	11
XM_005255318.1	negative	NOMO3	NODAL modulator 3%2C transcript variant X1	16
XM_011527241.2	negative	SNRNP70	small nuclear ribonucleoprotein U1 subunit 70%2C transcript variant X2	19
XM_011527694.1	negative	C2CD4C	C2 calcium dependent domain containing 4C%2C transcript variant X1	19
XM_011530976.2	negative	ARMCX6	armadillo repeat containing X-linked 6%2C transcript variant X1	X
XM_011531062.3	negative	ZMYM3	zinc finger MYM-type containing 3%2C transcript variant X2	X
XM_017012615.1	negative	C7orf26	chromosome 7 open reading frame 26%2C transcript variant X5	7
XM_017024403.2	negative	KCNH4	potassium voltage-gated channel subfamily H member 4%2C transcript variant X4	17
XM_017029748.1	negative	XAGE1A	X antigen family member 1A%2C transcript variant X1	X
NM_001282171.1	positive	KIR3DS1	killer cell immunoglobulin like receptor%2C three Ig domains and short cytoplasmic tail 1%2C transcript variant 3	19

Table S11.4. Twenty genes differentially expressed between patients with mild or severe disease in all three types of swabs

Gene ID	Relationship	Symbol	Function	Chromosome
NM_000805.5	negative	GAST	gastrin	17
NM_001078171.2	negative	RTL8C	retrotransposon Gag like 8C	X
NM_001098405.2	negative	GAGE12F	G antigen 12F	X
NM_001256852.1	negative	USP17L10	ubiquitin specific peptidase 17 like family member 10	4
NM_001256857.1	negative	USP17L17	ubiquitin specific peptidase 17 like family member 17	4
NM_001277423.1	negative	TCP11X2	t-complex 11 family%2C X-linked 2	X
NM_001351372.1	negative	NBPF26	NBPF member 26	1
NM_001355277.1	negative	CENPVL1	centromere protein V like 1	X
NM_001355278.1	negative	CENPVL2	centromere protein V like 2	X
NM_003512.4	negative	H2AC6	H2A clustered histone 6	6
NM_005354.6	negative	JUND	JunD proto-oncogene%2C AP-1 transcription factor subunit%2C transcript variant 1	19
NM_022144.3	negative	TNMD	tenomodulin	X
NM_175868.2	negative	MAGEA6	MAGE family member A6%2C transcript variant 2	X
NM_199294.3	negative	CENPS	centromere protein S%2C transcript variant A	1
NM_201222.3	negative	MAGED2	MAGE family member D2%2C transcript variant 3	X
NM_213725.2	negative	RPLP1	ribosomal protein lateral stalk subunit P1%2C transcript variant 2	15
XM_017000273.2	negative	OR2T10	olfactory receptor family 2 subfamily T member 10%2C transcript variant X1	1
XM_017011662.2	negative	RASA4	RAS p21 protein activator 4%2C transcript variant X2	7
XM_017029727.2	negative	TSPYL2	TSPY like 2%2C transcript variant X2	X
NM_001364814.1	positive	AMELY	amelogenin Y-linked%2C transcript variant 2	Y

SUPPLEMENTAL FIGURES

Supplementary Figure 1

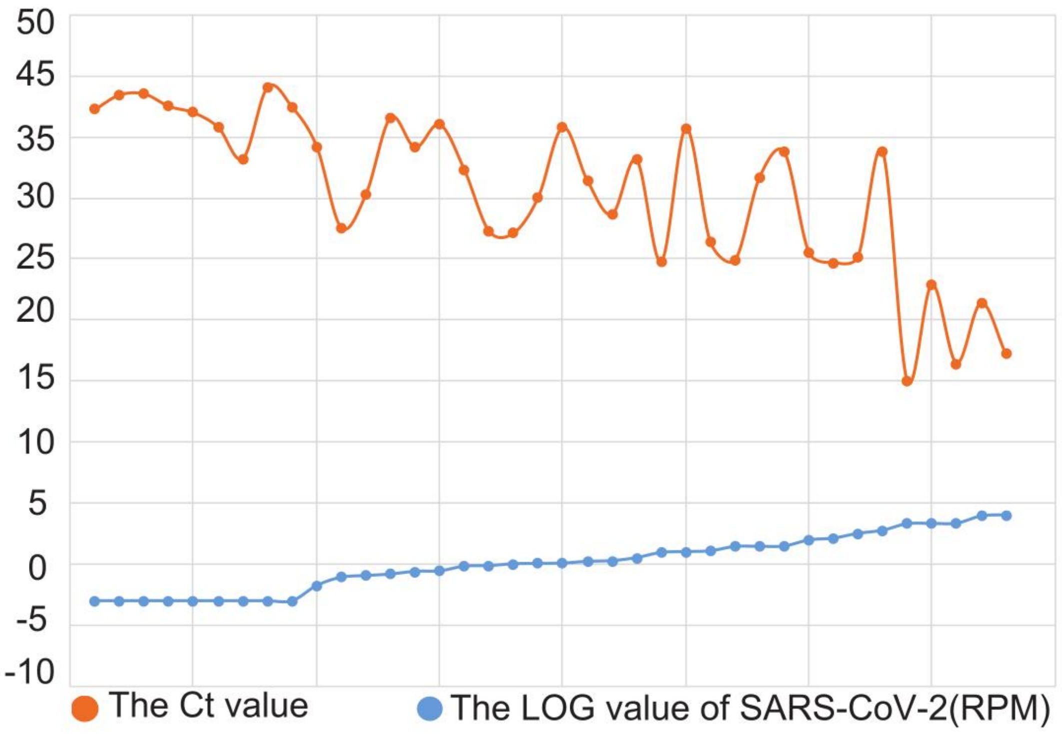

Supplementary Figure 1. Correlation between the cycle threshold (Ct) value (dark yellow) in quantitative RT-PCR and Reads detected (RPM) (blue) for SARS-CoV-2 virus DNA. Data were combined for all three types of swabs: anal, nasopharyngeal and oropharyngeal.

Supplementary Figure 2

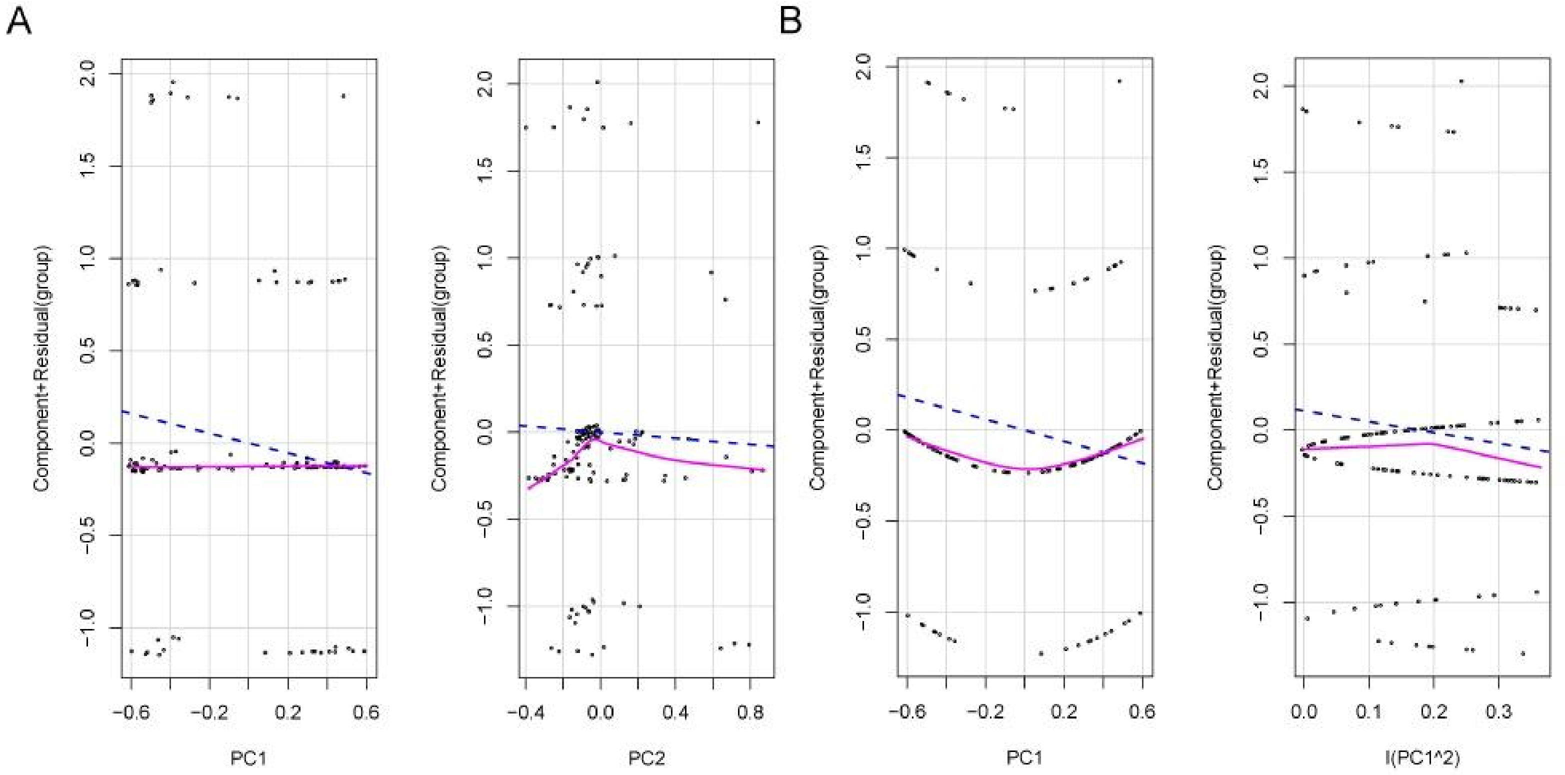

Supplementary Figure 2. The longitudinal analysis of PCA. (A) The relationship between PC1/PC2 factors and symptoms of mild, moderate, severe and critical was analyzed by simple linear regression model, p value of PC1 was 0.0833 and p value of PC2 was 0.7315. (B) The relationship between PC1 and symptoms of mild, moderate, severe and critical was analyzed by polynomial regression model, and p value of PC1 was 0.0564.

Supplementary Figure 3

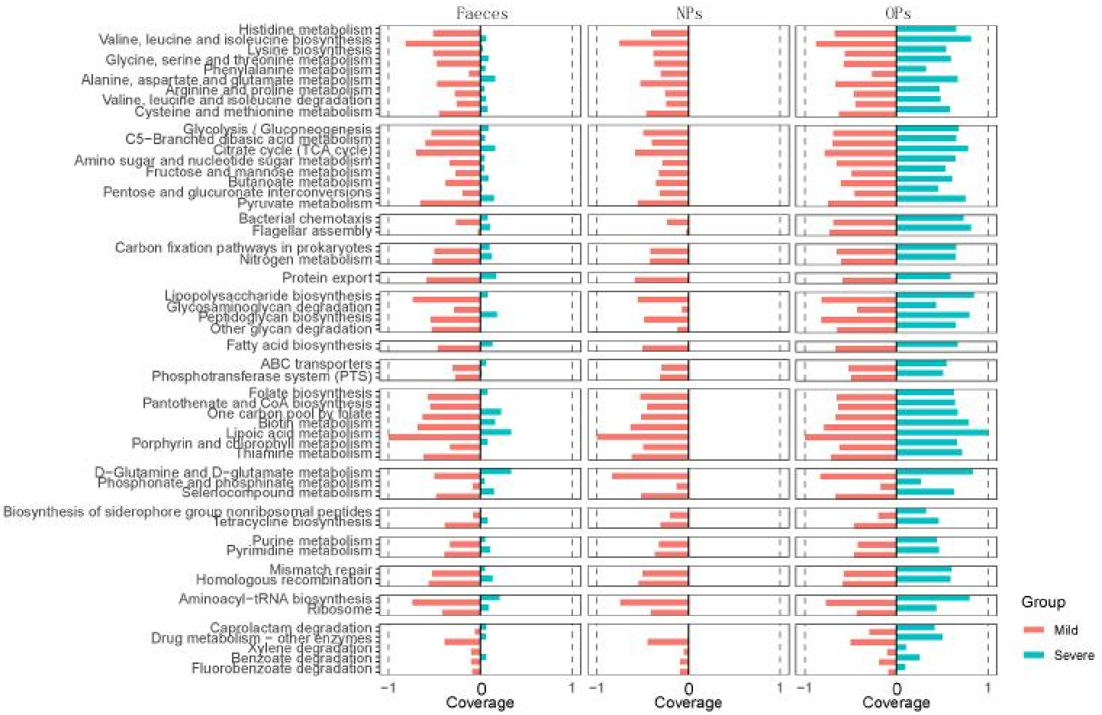

Supplementary Figure 3. The coverage of functional enrichment of microbial genes differentially expressed between patients with mild (red) or severe disease (cyan), based on Kyoto Encyclopedia of Genes and Genomes pathways. Results are shown separately for the three swab types.

Supplementary Figure 4

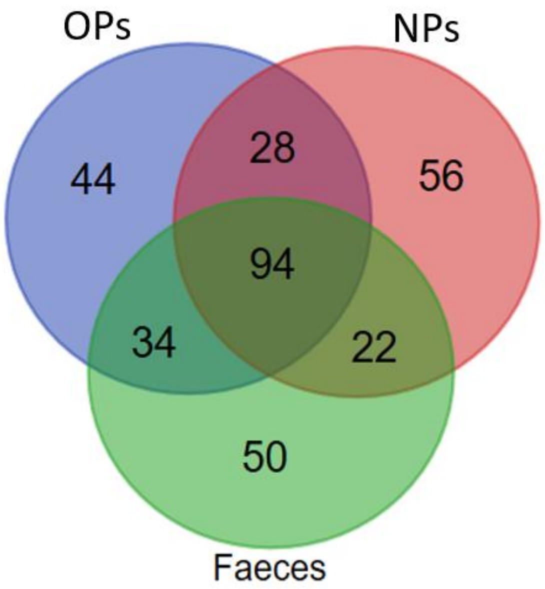

Supplementary Figure 4. Venn diagram depicting overlap among the 200 genes with the greatest differential expression between males and females based on anal (Faeces), nasopharyngeal (NPs) or oropharyngeal (OPs) swabs.

Supplementary Figure 5

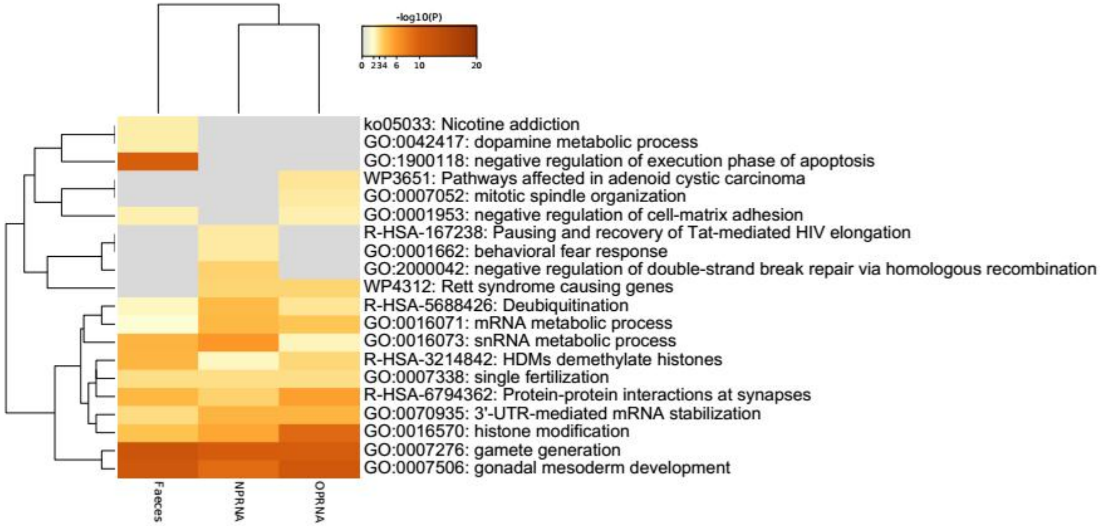

Supplementary Figure 5. Enrichment in Gene Ontology terms of the 200 genes with the greatest differential expression between patients with mild or severe COVID-19. Data are shown separately for anal (Faeces), nasopharyngeal (NPs), or oropharyngeal swabs (OPs).

Supplementary Figure 6

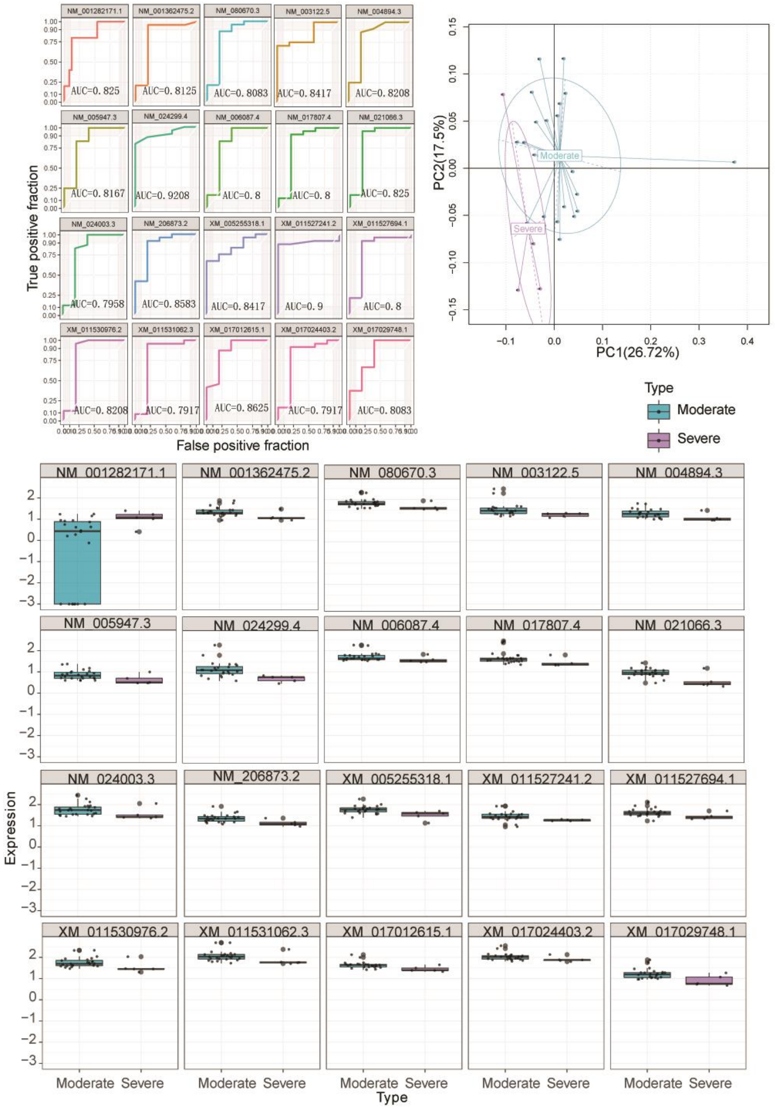

NM_001282171.1
NM_001362475.2
NM_080670.3
NM_003122.5
NM_004894.3
NM_005947.3
NM_024299.4
NM_006087.4
NM_017807.4
NM_021066.3
NM_024003.3
NM_206873.2
XM_005255318.1
XM_011527241.2
XM_011527694.1
XM_011530976.2
XM_011531062.3
XM_017012615.1
XM_017024403.2
XM_017029748.1
AUC=0. 825
AUC=0. 8125
AUC=0. 8083
AUC=0. 8417
AUC=0. 8208
AUC=0. 8167
AUC=0. 9208
AUC=0. 8
AUC=0. 8
AUC=0. 825
AUC=0. 7958
AUC=0. 8583
AUC=0. 8417
AUC=0. 9
AUC=0. 8
AUC=0. 8208
AUC=0. 7917
AUC=0. 8625
AUC=0. 7917
AUC=0. 8083
True positive fraction
False positive fraction
PC2(17.5%)
PC1(26.72%)
Moderate
Severe
Type
Moderate
Severe
Expression
Moderate Severe
Type

Supplementary Figure 6. Biomarker signatures predict COVID-19 severity in nasopharyngeal swabs. (A) ROC curve analysis for the predictive power of selected 20 genes by random forest for distinguishing severe from mild groups. (B) Principle component analysis for the mild (blue) and sever (purple) COVID-19 patients based on selected 20 genes. (C) Selected 20 genes expression values for mild (blue) or severe (purple) COVID-19 patients.

Supplementary Figure 7

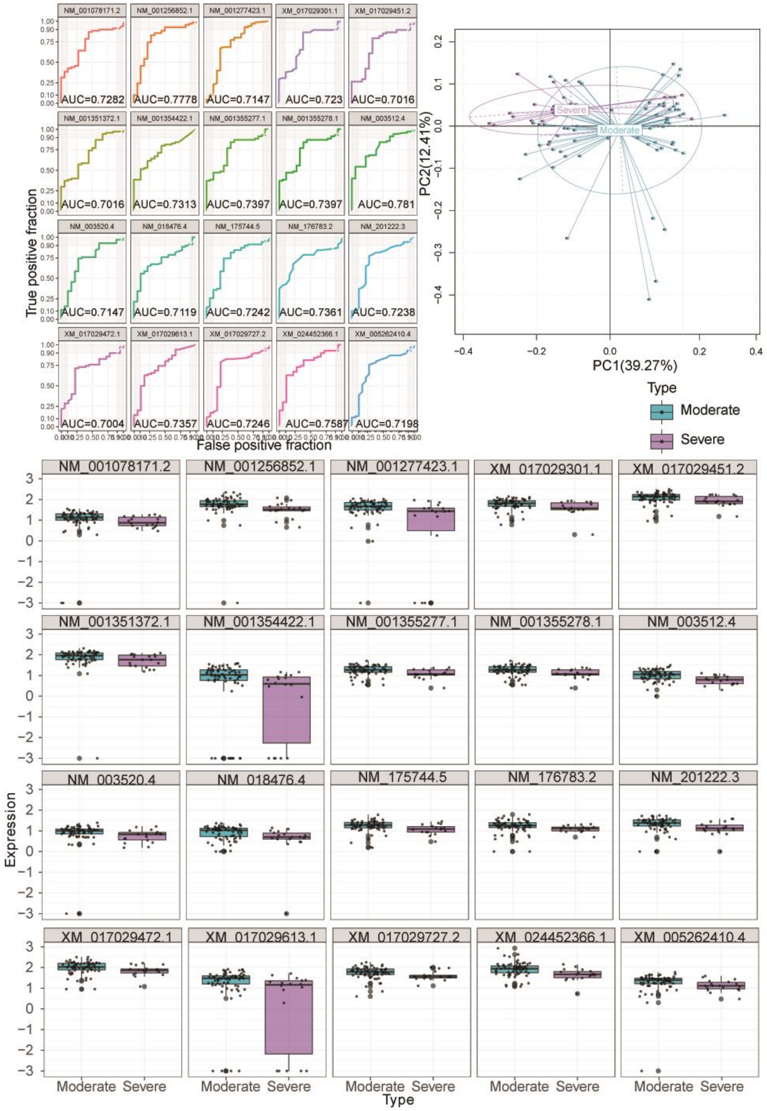
True positive fraction
False positive fraction
AUC=0.7282
AUC=0.7778
AUC=0.7147
AUC=0.723
AUC=0.7016
AUC=0.7016
AUC=0.7313
AUC=0.7397
AUC=0.7397
AUC=0.781
AUC=0.7147
AUC=0.7119
AUC=0.7242
AUC=0.7361
AUC=0.7238
AUC=0.7004
AUC=0.7357
AUC=0.7246
AUC=0.7587
AUC=0.7198
PC2(12.41%)
PC1(39.27%)
Severe
Moderate
Type
Moderate
Severe
NM_001078171.2
NM_001256852.1
NM_001277423.1
XM_017029301.1
XM_017029451.2
NM_001351372.1
NM_001354422.1
NM_001355277.1
NM_001355278.1
NM_003512.4
NM_003520.4
NM_018476.4
NM_175744.5
NM_176783.2
NM_201222.3
XM_017029472.1
XM_017029613.1
XM_017029727.2
XM_024452366.1
XM_005262410.4
Expression
Moderate Severe
Type

Supplementary Figure 7. Biomarker signatures predict COVID-19 severity in oropharyngeal swabs. (A) ROC curve analysis for the predictive power of selected 20 genes by random forest for distinguishing severe from mild groups. (B) Principle component analysis for the mild (blue) and sever (purple) COVID-19 patients based on selected 20 genes. (C) Selected 20 genes expression values for mild (blue) or severe (purple) COVID-19 patients.

Supplementary Figure 8

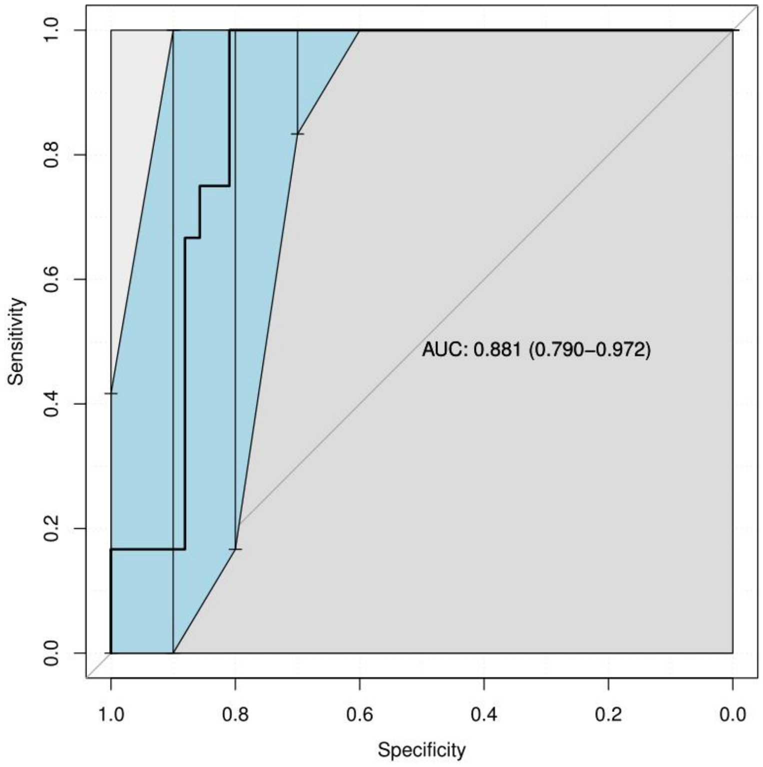

Supplementary Figure 8. Receiver operating characteristic curve analysis to assess the ability of a D-dimer concentration determined by random forest modeling to distinguish patients with mild or severe COVID-19. AUC, area under the curve.
